# Geodynamic evolution of southwestern North America since the Late Eocene

**DOI:** 10.1038/s41467-019-12950-8

**Published:** 2019-11-18

**Authors:** Alireza Bahadori, William E. Holt

**Affiliations:** Department of Geosciences, Stony Brook University, Stony Brook, NY USA

**Keywords:** Geodynamics, Tectonics

## Abstract

Slab rollback, lithospheric body forces, or evolution of plate boundary conditions are strongly debated as possible lithospheric driving mechanisms for Cenozoic extension in southwestern North America. By incorporating paleo-topography, lithospheric structure, and paleo-boundary conditions, we develop a complete geodynamic model that quantifies lithospheric deviatoric stresses and predicts extension and shear history since Late Eocene. We show that lithospheric body forces together with influence of change-over from subduction to transtensional boundary conditions from Late Eocene to Early Miocene were the primary driving factors controlling direction and magnitude of extensional deviatoric stresses that produced topographic collapse. After paleo-highlands collapsed, influence of Pacific-North America plate motion and associated deformation style along the plate boundary became increasingly important from Middle Miocene to present. Smaller-scale convection stress effects from slab rollback and associated mantle flow played only a minor role. However, slab rollback guided deformation rate through introduction of melts and fluids that impacted rheology.

## Abstract (web summary)

The tectonic evolution of southwestern North America remains debated. Here, the authors present a complete time-dependent geodynamic model of the tectonic evolution of southwestern North America, which can explain the extensional collapse of the Basin and Range Province since the Late Eocene.

## Introduction

**Fig. 1:**
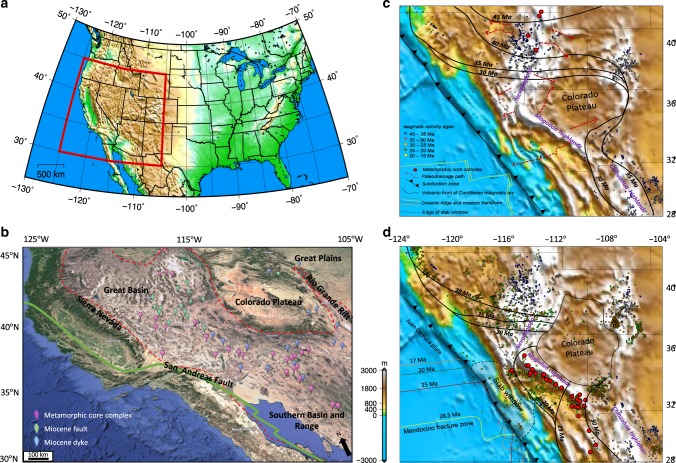
Northeast Pacific and southwestern North American plate tectonic evolution since the Oligocene. **a** Map of surface elevation in southwestern North America and the location of Basin and Range Province. **b** Satellite image of southwestern North America showing the location of the Basin and Range Province, Colorado Plateau, Rio Grande Rift, San Andreas Fault System, along with present-day positions of MCCs, and Miocene faults and dykes. **c** Map of paleo-elevation model of southwestern North America at 31 Ma from Bahadori et al.^8^ showing boundary condition changes, slab rollback development (black contours), MCCs, and ignimbrite flare up from Dickinson^1^ as well as paleo-drainage patterns (dashed red arrows) from Henry et al.^26^; **d** same as “**c**” but at 27 Ma. Panels “**c**” and “**d**” show the reconstructed positions of magmatism and MCCs at 31 and 27 Ma, respectively

The topographic evolution of mountain belts and continental interiors reflects directly upon interactions among continental dynamics, mantle convection, and climatic and erosional processes. The Basin and Range Province of southwestern North America is a unique modern intracontinental extensional province following Sevier–Laramide phases of crustal shortening and mountain building (~155–60 Ma) that initiated in Late Eocene (~40 Ma)^1^. Slab rollback, wide-spread volcanism, and the development of the San Andreas Fault System (SAFS)^1–5^ followed the earlier shallow- to flat-subduction of the east dipping Farallon slab. During this plate margin transition^6,7^ high elevations of orogenic plateaus underwent profound extension and shear that resulted in the present-day Basin and Range Province^1,6,8–12^ (Fig. 1a, b). Although significant progress has been made toward quantifying the dynamics of the plate boundary zone in southwestern North America, a complete description of the time-dependent driving forces behind this complex strain history has remained contentious. Competing hypotheses for the driving mechanisms of this extension include the response to slab rollback, mantle flow and lithosphere delamination^5,13–17^, lithospheric body forces generated by high topography, crustal thickness, and upper mantle density variations^2,3,9,18–22^, or the evolution of plate boundary conditions along the westernmost margin^6^. There is controversy about how much of the paleo-highland (e.g., Nevadaplano) was associated with dynamic topography^15,20^ versus static support (crustal root)^10,23–25^. Another controversy is whether the collapse was controlled by boundary conditions changing along the plate margin or whether it also required significant lithospheric weakening. These driving mechanisms are not mutually exclusive and likely operated together, with a variable weighting of their respective contributions over time.

Slab rollback under the Basin and Range Province triggered volcanism and initiated an ignimbrite flare up by influx of hot asthenosphere underneath the overriding North American plate^1^. The distribution of Cenozoic ash-flow tuffs within the Basin and Range Province implies that west and east-trending rivers drained off a north–south trending paleo-highland through eastern Nevada^26^ (Fig. 1c). Bahadori et al.^8^ have used displacement histories on land in southwestern North America from the model of McQuarrie and Wernicke^11^ to develop a model of finite strain history, crustal thicknesses, and paleo-elevations since Late Eocene. Their paleo-elevation model suggests that as much as 75% of the high paleo-elevations were associated with a thick crustal root (~60 km), with the remainder supported by low density upper mantle. This is in agreement with recent paleo-altimetry results supporting the presence of a high Nevadaplano (~3500 m) in southwestern North America during Late Cretaceous prior to the onset of slab rollback, and that following slab rollback there was an additional uplift of less than 600 m^25,27,28^. Crustal stretching and topographic collapse within areas of highest extension resulted in significant exhumation of deep crustal rocks in metamorphic core complexes (MCCs)^9,29,30^ of southwestern North America (Fig. 1b–d). In northern Basin and Range Province, the slab rollback progressed during Eocene and Oligocene (~55–20 Ma) from northeast to southwest and resulted in MCC ages that young towards the south^30,31^ (Fig. 1c). In the south (southern Arizona and New Mexico, Northern Mexico) the slab rollback progressed from east to west during Late Eocene to Early Miocene (~35–17 Ma), resulting in MCCs that young toward the west^31^ (Fig. 1d). The youngest MCCs, developed during Miocene (~17–5 Ma), coincide with the western edges of the southwestern Great Basin within southern Nevada and eastern California^1^ (Fig. 1b).

Here, we present a complete geodynamic model that incorporates time-dependent estimates of paleo-crustal thickness and paleo-topography from Bahadori et al.^8^, together with accurately determined paleo-boundary conditions^6,7^ and interior surface kinematics from geological constraints^11^, to explain the full extension and shear history in southwestern North America since Late Eocene. Our geodynamic approach provides the opportunity to rigorously quantify the magnitudes of lithospheric body forces over time, enabling a test of the relative contributions of lithospheric driving mechanisms responsible for the history of extension and topographic collapse that led to the present-day Basin and Range Province. By separating out the effects of internal body forces from the influences of the accommodation of relative plate motions we provide important new insights into the dynamics of the plate boundary zone evolution in southwestern North America and show for the first time the actual sources and primary driving factors for deformational features over time.

## Results

### An optimal geodynamic solution

**Fig. 2:**
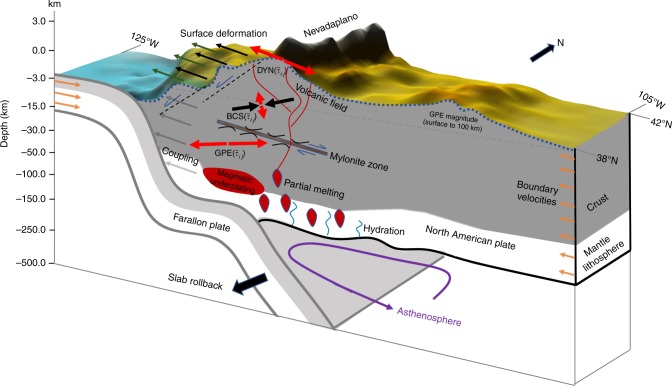
Primary driving forces for deformation of the continental lithosphere. A schematic of inputs and outputs for estimating primary driving forces in our geodynamic calculations. GPE $(\bar \tau _{ij})$ represents deviatoric stress associated with gravitational potential energy gradients. BCS $(\bar \tau _{ij})$ represents boundary condition stress associated with plate motion. DYN $(\bar \tau _{ij})$ represents dynamic deviatoric stresses from our iterative dynamic model

**Fig. 3:**
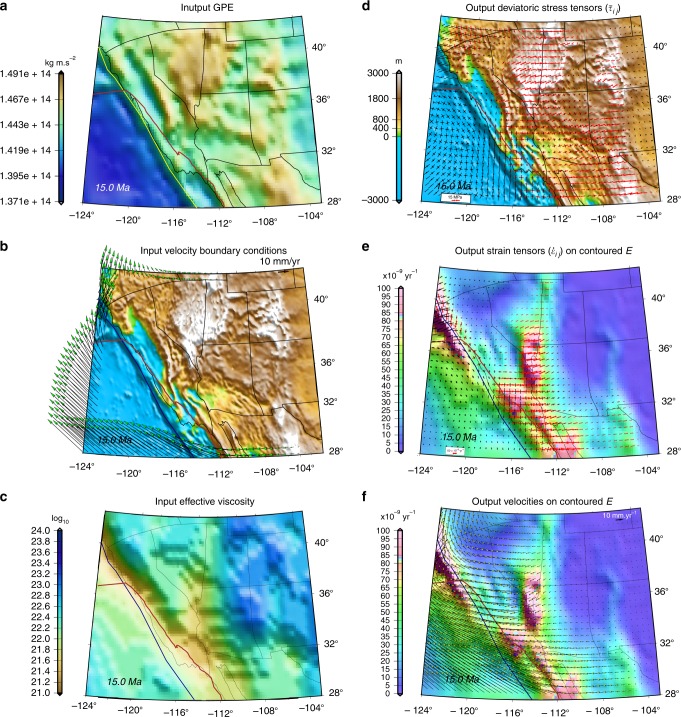
Inputs and outputs of geodynamic calculations for benchmarking at Middle Miocene in southwestern North America. **a** Map of contoured GPE estimates. **b** Boundary condition velocity estimates based on accommodation of Farallon- and Pacific-North America relative plate motions^6,7^ superimposed on paleo-elevation model of Bahadori et al.^8^. **c** Map of lithospheric effective viscosity that must be recovered in benchmarking test. **d** Map of principal axes of lithospheric deviatoric stress tensors obtained in solution to force-balance using inputs in “**a**–**c**” superimposed on paleo-elevation model of Bahadori et al.^8^ (see Methods). Red arrows represent tensional, and black arrows represent compressional principal axes of deviatoric stress tensors that must be recovered in benchmarking test. **e** Map of contoured second invariant of strain rates (*E*) and principal axes of strain rate tensors obtained from force-balance solution with inputs from “**a**–**c**”. Red arrows represent tensional, and black arrows represent compressional principal axes of strain tensors. **f** Map of contoured second invariant of strain rates (*E*) and model velocities relative to the North American frame achieved from force-balance solution from inputs in “**a**–**c**”. Red line in all panels represents reconstructed position of the East Pacific Rise and SAFS. Blue and yellow lines in all panels represent reconstructed position of continental edge

Dynamic calculations require accurate velocity boundary conditions, along with accurate interior surface kinematics (Fig. 2). We have produced a time-dependent kinematic solution that preserves the land-based observations^11^, but that also includes the accommodation of the Farallon- and Pacific-North America relative plate motions^6,7^ from Late Eocene to present, with the influence of the change-over from subduction boundary conditions to the progressive development of the SAFS with northward migration of the Mendocino Triple Junction (MTJ) (Supplementary Movie 1 and Supplementary Data 1). We use the lithospheric structure, paleo-topography, and upper mantle density models of Bahadori et al.^8^ to define depth-integrated vertical stresses, or gravitational potential energy per unit area (hereby called GPE), along with their uncertainties. With a defined distribution of GPE (Fig. 3a), velocity boundary conditions relative to the North American plate^6,7^ (Fig. 3b), and lateral variations in depth-integrated effective viscosity of the lithosphere in southwestern North America (Fig. 3c), we solve the depth-integrated 3-D spherical form of the force-balance equations^18,32,33^ (Methods) for estimates of depth-integrated (from surface to 100 km below sea level) horizontal deviatoric stresses, strain rates, and relative velocities (Fig. 3d–f) (Supplementary Note 1 and Supplementary Fig. 1).

### Recovery of true effective viscosity and deviatoric stresses

**Fig. 4:**
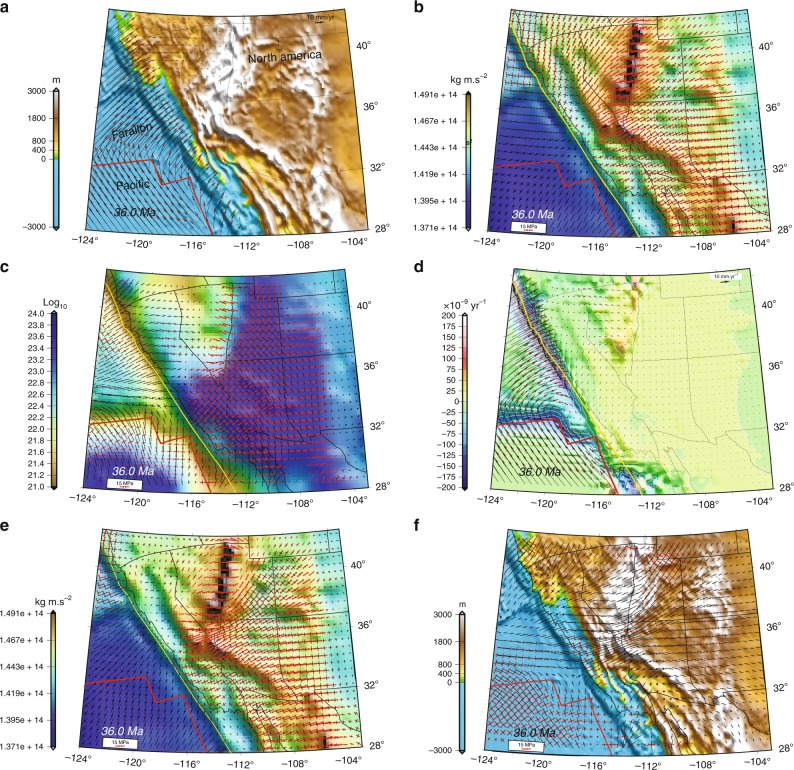
Kinematics and dynamics of lithosphere in southwestern North America at Late Eocene. **a** Kinematic estimates based on land-based geological observations and also accommodation of Farallon- and Pacific-North America relative plate motions^6,7^ in southwestern North America superimposed on paleo-elevation model of Bahadori et al.^8^. Red vectors represent model velocities relative to the North American frame. **b** Map of contoured GPE estimates. Arrows represent principal axes of vertically averaged horizontal deviatoric stresses associated with GPE gradients based on an initially constant viscosity value for the lithosphere. **c** Map of lithospheric effective viscosity and principal axes of deviatoric stresses obtained in final iterative dynamic model. **d** Map of dilatational strain rates and model velocities relative to the North American frame achieved from iterative dynamic model. **e** Map of principal axes of deviatoric stress associated with GPE (contoured) gradients, obtained using final estimates of laterally varying effective viscosity values in “**c**”. **f** Map of the stress field boundary conditions obtained by subtracting stress values in “**e**” from stress values in “**c**” superimposed on paleo-elevation model of Bahadori et al.^8^. For all panels red arrows represent tensional, and black arrows represent compressional principal axes of deviatoric stress. Red line in all panels represents reconstructed position of the East Pacific Rise and SAFS. Yellow line in all panels represents reconstructed position of continental edge

**Fig. 5:**
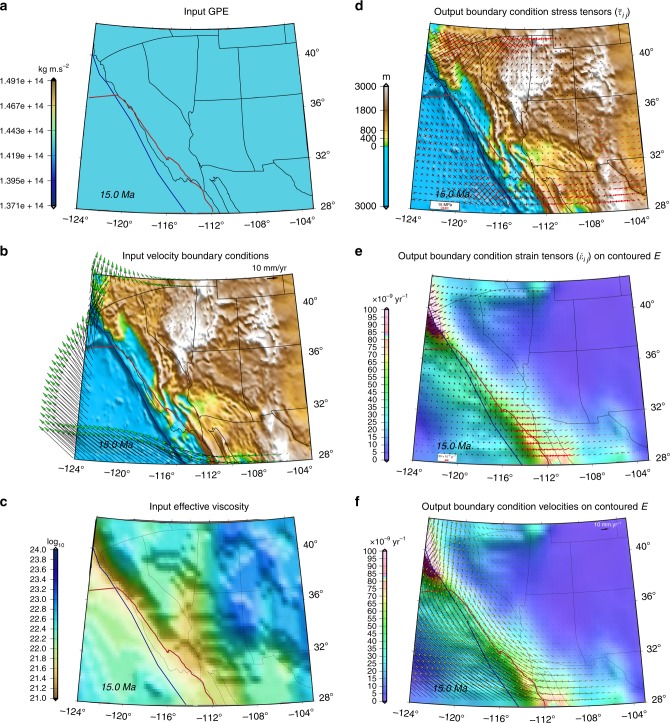
Inputs and outputs for geodynamically constrained boundary conditions at Middle Miocene in southwestern North America. **a** Map of an initially constant GPE value for the lithosphere. **b** Boundary condition velocity estimates based on accommodation of Farallon- and Pacific-North America relative plate motions in southwestern North America. **c** Map of lithospheric effective viscosity obtained in final iterative dynamic model. **d** Map of principal axes of lithospheric boundary condition stress tensors superimposed on paleo-elevation model of Bahadori et al.^8^. Red arrows represent tensional, and black arrows represent compressional principal axes of stress tensors. **e** Map of contoured second invariant of boundary condition strain rates (*E*) and principal axes of boundary condition strain tensors. Red arrows represent tensional, and black arrows represent compressional principal axes of strain tensors. **f** Map of contoured second invariant of boundary condition strain rates (*E*) and model boundary condition velocities relative to the North American frame. Red line in all panels represents reconstructed position of the East Pacific Rise and SAFS. Blue line in all panels represents reconstructed position of continental edge

Through blind test benchmarking we show that if paleo-topography and crustal structure (GPE), plate motions, and scalar values of strain rates (*E*) are known (Fig. 4a, b), then it is possible to recover the true depth-integrated effective viscosity and deviatoric stresses within the lithosphere (Fig. 4c) (Methods). We first solve force-balance equations for depth integrated horizontal deviatoric stresses associated with GPE distributions alone (*T*_GPE_), assuming constant viscosity everywhere and zero velocity boundary conditions along a rigid boundary (Fig. 4b) (Methods). This solution provides initial estimates of the distribution of deviatoric stresses that balance gradients of GPE. We then estimate an initial distribution of viscosity $\bar \eta = \frac{T}{E}$, where *E* is the second invariant of strain rates from our kinematic solution. Using this laterally varying first estimate of the distribution of $\bar \eta$, we seek the complete solution by solving the force-balance equations, using the distribution of GPE and velocity boundary conditions appropriate for the given time interval (Methods). This provides updated values of *T*, model values of strain rates $\dot \varepsilon _{\alpha \beta }$, and new $\bar \eta$. The procedure for the complete solution is repeated until there is convergence (no further changes in *T* and $\dot \varepsilon _{\alpha \beta }$) (Fig. 4c, d). This complete solution, which we term the iterative dynamic model solution, can be thought of as the combination of stresses associated with GPE gradients, achieved using final estimates of $\bar \eta$ (Fig. 4e), and stresses that accommodate the relative plate motion, which we term boundary condition stresses (BCS) (Fig. 4f). We determine the BCS in two separate ways. First, subtracting a solution of the stresses that balances GPE gradients alone, using final estimates of laterally varying effective viscosity and without any applied plate motion (Fig. 4e), from the complete solution that contains the effects of both velocity boundary conditions and GPE gradients together (Fig. 4c). Second, solving force-balance equations, using zero GPE gradients and the final viscosity structure, while applying the correct velocity boundary conditions (Fig. 5a–c). These two methods provide BCS solutions that agree. The BCS solutions yield important information on boundary condition strain tensors and velocities (Fig. 5d–f) (Methods, Supplementary Notes 2–4, and Supplementary Figs. 2–4).

### Check of the consistency between force-balance and rheology

**Fig. 6:**
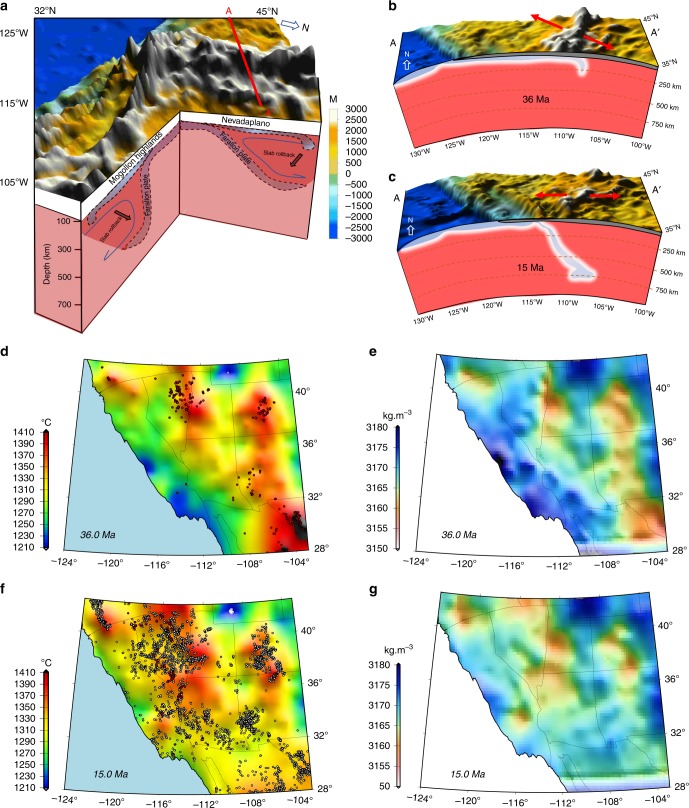
Role of Farallon slab rollback on ignimbrite flare up, upper mantle density variation, and landscape evolution. **a** 3-D perspective image of paleo-elevation of Nevadaplano and Mogollon Highlands at 36 Ma from model of Bahadori et al.^8^ along with development of slab rollback. **b**, **c** Progressive slab tearing at latitude 41°N at Late Eocene (36 Ma) and Middle Miocene (15 Ma) from Liu and Stegman^17^ underneath the Nevadaplano from model of Bahadori et al.^8^. Red arrows show general directions of deviatoric tension obtained in dynamic model that incorporates body forces (gradients in GPE) and velocity boundary conditions. **d**, **f** Maps of averaged upper mantle temperature from Moho boundary to 100 km depth. Red and grey dots represent reconstructed coordinates of present-day localities of paleo-magmatism through time. Red dots represent active magmatism and grey dots represent previously active magmatism. **e**, **g** Maps of upper mantle density changes inferred from models of lithospheric pressure, upper mantle temperature, and volumetric degree of melting for upper mantle

Using an upper mantle temperature model that relies on seismic shear wave velocities^34^ and a steady-state conductive heat distribution model, associated with positions and migrations of volcanism^35^ from Late Eocene to present (Fig. 6a–d, f) (Supplementary Movie 2), we separately investigate the causal mechanisms (the role of melts^36,37^ and fluids^38^) on lithospheric rheological variations obtained from the force-balance solutions (Figs. 3d, 4c, and 5c). With these causal mechanism solutions, lithospheric effective viscosity variations are attributed to either a molten upper mantle phase that includes wet or dry partial melting for upper mantle or a non-molten upper mantle phase that includes either hardening or wet partial melting of lower crust (Methods). We consider an initial wet upper mantle in Late Eocene for our causal mechanism models based on evidence supporting the hydration of the base of the North American lithosphere by de-watering of Farallon slab during its Laramide flat subduction^39–42^. The calculated histories in temperature, partial melting, and hardening are most likely linked to the slab rollback history (Fig. 6a–c).

Furthermore, given the constraints of lithospheric pressure, upper mantle temperature, and our causal mechanism solutions, we independently estimate the effective density^37^ for the upper mantle through time and compare this with the density model embedded in our dynamic solution (Methods). Our calculated densities for the upper mantle at Late Eocene show low densities beneath Nevadaplano, Rockies and westernmost portions of Texas (Fig. 6e, g) (Supplementary Movie 3). These low initial densities beneath Nevadaplano are consistent with the hypothesis that the corresponding thickened mantle lithosphere root, acquired during the shortening phases of deformation^14^, had likely already been removed prior to Late Eocene^16,43^, and is also consistent with observations that paleo-elevations of Nevadaplano were already high in latest Cretaceous and achieved little additional uplift in the Cenozoic^25,27,44^. Hence, the starting paleo-elevation model^8^ at Late Eocene (~36 Ma) postdates any uplift that was associated with possible loss of the lithospheric root.

### Role of body forces and plate motions in deformation history

**Fig. 7:**
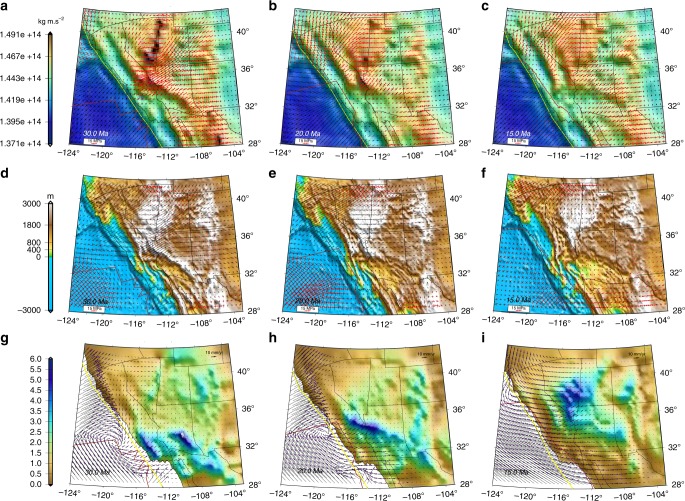
Depth integrated horizontal deviatoric stresses associated with GPE differences and boundary condition forces. **a**–**c** Time-slice maps of contoured GPE estimates in southwestern North America. Arrows represent vertically averaged horizontal deviatoric stress field associated with GPE variations within the lithosphere. **d**–**f** Time-slice maps of the stress field boundary condition superimposed on paleo-elevation model of Bahadori et al.^8^. Red arrows represent tensional principal axes of deviatoric stress, and black arrows represent compressional principal axes of deviatoric stress tensors. **g**–**i** Time-slice maps of ratio of *T*_GPE_/*T*_BCS_. Purple vectors are model dynamic velocities relative to North America. Red line in all panels represents reconstructed position of the East Pacific Rise and San Andreas Fault System. Yellow line in all panels represents reconstructed position of continental edge

**Fig. 8:**
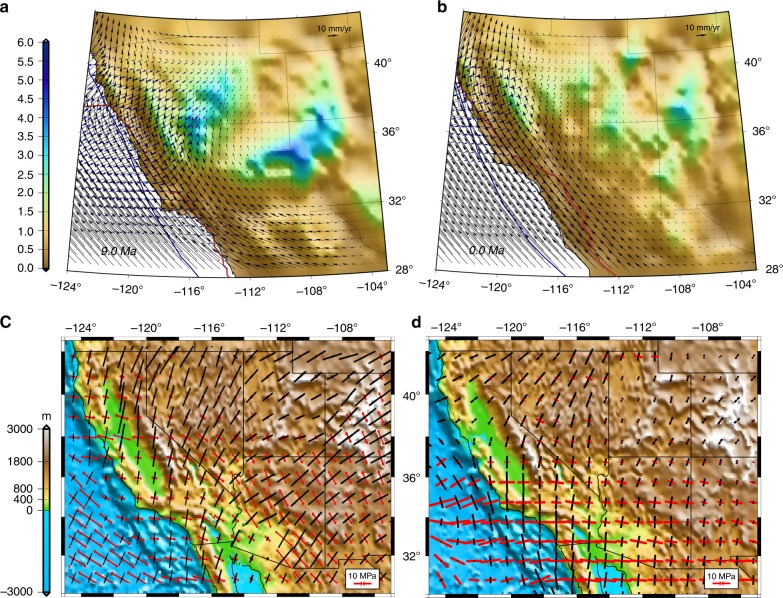
Long-wavelength effect of mantle flow within the Basin and Range Province. **a** Map of ratio of *T*_GPE_/*T*_BCS_ at 9 Ma. Blue vectors are velocity field estimates relative to North America associated with GPE. Black vectors are boundary condition velocity field estimates relative to North America; **b** same as “**a**” but at present-day. Red line represents reconstructed position of the East Pacific Rise and SAFS. Blue line represents reconstructed position of continental edge. **c** Deviatoric stresses from lithosphere coupling with global mantle flow S40RTS^46^. **d** Boundary condition solution of deviatoric stresses at present-day (comparison of the compressional directions and magnitudes in both models within the Basin and Range Province emphasizes the long-wavelength effect of mantle flow)

To quantify the relative role of GPE related stresses to BCS (Fig. 7a–f) we compute the ratio of the second invariants of stresses associated with GPE gradients (*T*_GPE_) and boundary conditions (*T*_BCS_) through time (Fig. 7g–i) (Supplementary Movies 4–8). A remarkable feature is that this ratio is dramatically increased just prior to extensional collapse of topography and the increasing values of the ratio that migrate northward as the MTJ migrates north. The ratio increase is associated with a dramatic drop in compressional stresses within land areas south of the MTJ. Thus, Arizona is impacted first as the EPR impinges on the trench during Oligocene (~30–20 Ma) and the ratio jumps from 1.5 to 5 (Fig. 7g, h). Note that prior to the impact of the EPR (Late Eocene), extension is limited only to a zone along the Nevadaplano in the highest paleo-topography, with stretching in a NW–SE direction (Figs. 4c and 6b). During Early Miocene (~20–15 Ma) that ratio increases within regions of western Arizona and southern Nevada (Fig. 7h, i). By 11 Ma the Nevadaplano topography has collapsed, ratio values decrease, and the plate boundary zone is increasingly influenced by Pacific-North America relative motion. From Middle Miocene (~11 Ma) to present the ratio is ~0.5–3, where the lower values (~0.5) are for regions closer to the plate boundary zone and high values (~3) are for central and eastern Great Basin and eastern portions of the southern Basin and Range Province (Fig. 8a, b). The ratios for present-day are consistent with previous findings, arguing that the western Great Basin is strongly influenced today by the Pacific Plate relative motions^3,18,22,45^ (reflecting the importance of boundary conditions), and that GPE and boundary condition contributions are near parity within central and eastern Great Basin^3,18^.

### Negligible influence of smaller scale convection on stress

The magnitude and direction of deviatoric stresses that arise from global calculations of mantle flow coupling with the lithosphere^46^ (Fig. 8c) appear similar to our model BCSs for present-day (Fig. 8d). This agreement suggests that it is the longer-wavelength component of the horizontal traction field (which integrates over thousands of kilometers to become important) associated with mantle convection^46^ that is reflected in boundary condition stress field solutions for southwestern North America. That is, the distances between plate margin edge and interior deformation zone are small enough such that tractions directly below deforming regions can be ignored without introducing large errors to our stress solutions (Methods, Supplementary Note 5, and Supplementary Fig. 3). Thus, tractions associated with smaller-scale convection, such as slab rollback before, during, and after the collapse of the Mogollon Highlands and Nevadaplano, are expected to play a much lesser role in their influence on the stress field than do the GPE gradients, which are modeled in this study.

### History and mechanisms for lithospheric geodynamic evolution

**Fig. 9:**
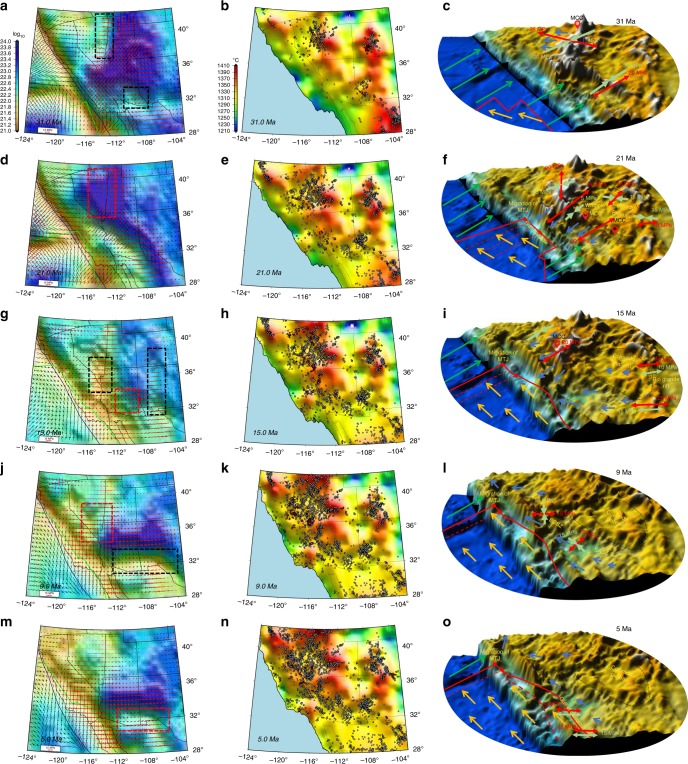
Dynamically constrained refined prediction of deviatoric stress and effective viscosity variations. **a**, **d**, **g**, **j**, **m** Time-slice maps of lithospheric effective viscosity obtained from deviatoric stresses of iterative dynamic model in southwestern North America. Red arrows represent tensional, and black arrows represent compressional principal axes of deviatoric stress tensors. Red dashed lines show the location of viscosity hardening and black dashed lines show the location of viscosity weakening. **b**, **e**, **h**, **k**, **n** Time-slice maps of averaged upper mantle temperature from Moho boundary to 100 km depth. Red and grey dots represent reconstructed coordinates of present-day localities of paleo-magmatism through time. Red dots represent active magmatism and grey dots represent previously active magmatism. **c**, **f**, **i**, **l**, **o** Time-slice 3-D perspective images of paleo-elevation in southwestern North America along with major tectonic events and driving mechanisms (direction and magnitude) responsible for extensional collapse of a distribution of high topography since Late Eocene. Light green arrows represent contractional stresses related to the accommodation of Pacific- and Farallon-North America relative motion. Red arrows represent tensional deviatoric stresses. Yellow vectors represent the motion of Pacific plate, green vectors represent the motion of Farallon plate, and blue vectors represent the motions of deforming lithosphere. All motions are relative to the North American plate

**Fig. 10:**
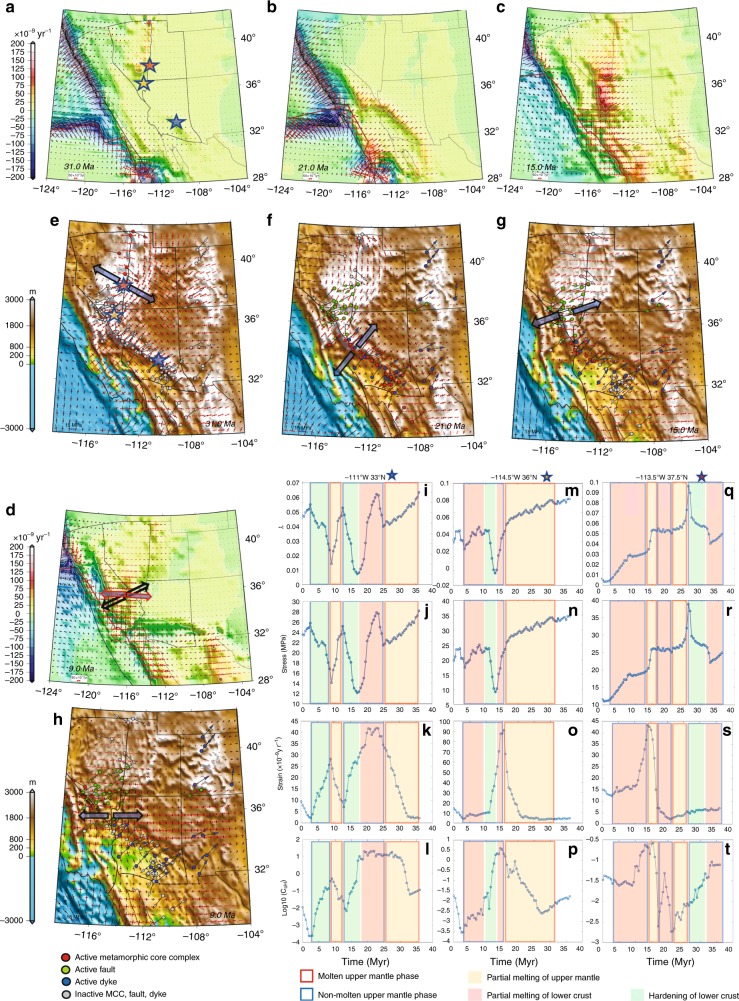
Causal mechanism for geodynamic evolution of southwestern North America and its correlation with geological observations. **a**–**d** Time-slice maps of contoured dilatation strain rates obtained from iterative dynamic model at 31, 21, 15, and 9 Ma. Red arrows represent tensional, and black arrows represent compressional principal axes of strain tensors. **e**–**h** Plots of correlations between stretch directions of MCCs (red dots), Miocene faults (green dots), and Miocene dykes (blue dots) and the orientation of the tensional deviatoric stresses from iterative dynamic model superimposed on paleo-elevation model of Bahadori et al.^8^. Red arrows represent tensional, and black arrows represent compressional principal axes of deviatoric stress tensors at 31, 21, 15, and 9 Ma. Vectors represent the rotation of principal axes of extensional strain rates. **i**–**t** Plots of melt and fluid pressure factor (*λ*), stress magnitudes (*T*_dyn_), strain rates (*E*), and *C*_OH_ estimates for coordinates −111°W 33°N, −114.5°W 36°N, and −113.5°W 37.5°N

Our time-dependent dynamic models of effective viscosity and horizontal deviatoric stresses of the lithosphere show early extensional collapse of only the highest elevations of the Nevadaplano during Late Eocene to Early Oligocene (36–31 Ma), with low effective viscosities ($\bar \eta$) of 10^22.2^ ± 10^21^ Pa s (Fig. 9a) and a gradual reduction of second invariant of deviatoric stresses (*T*_dyn_) from 25 ± 9 to 20 ± 8 MPa. The NW–SE extension, in agreement with MCC locations and orientations of stretch^1^, is at an angle of about ~45° to the contractional stresses associated with accommodation of Farallon-North America motion and is limited to highest GPE zones within the Nevadaplano region (*T*_GPE_/*T*_BCS_ = ~3) (Figs. 4c, f, 7g, and 9c) (Supplementary Notes 7–9, Supplementary Tables 1 and 2, and Supplementary Movies 9–12). This extension differs from the McQuarrie and Wernicke^11^ model, which shows a more region-wide extension during this time interval in what is now the present-day Great Basin. This misfit in our dynamic model is caused by the need to accommodate the convergent relative motion between Farallon and North America. This feature of the dynamic model is similar to the present-day setting in the Altiplano of South America, which shows extension directions at highest elevations at an acute angle of about ~45° to the contractional stresses^47^ associated with South America-Nazca plate motion boundary conditions. Our temperature model shows an increase from ~1310 to ~1370 °C (Fig. 9b) during this time interval (36–31 Ma). The calculated changes in melt pressure factor (*λ*_*m*_) and fluid pressure factor (*λ*_*f*_) suggest a nonmolten phase (where total extracted melt fraction, *M*_ext_, is greater than volumetric degree of melting, *M*_0_) for upper mantle. The reduction of *λ*_*f*_ from 0.05 to 0.035 and high estimates of distributed water in nominally anhydrous minerals (*C*_OH_) (log_10_*C*_OH_ = −1) demonstrate the increase of fluid pressure (*P*_*f*_) and wet partial melting of lower crust during 36–31 Ma, which we interpret to have played a critical role for reduction of $\bar \eta$ and is consistent with high-K, calc-alkaline volcanic rocks with low Fe/Mg ratios at Late Eocene^35,48,49^ in the area (Fig. 10a, e, q–t) (Supplementary Movies 13–16).

During Oligocene (31–22 Ma) the Nevadaplano region undergoes a viscosity hardening for the lithosphere ($\bar \eta$ increases from 10^22.2^ ± 10^21^ to 10^23.4^ ± 10^22^ Pa s), a shift to N–S extension, with *E* dropping from 40 ± 8 to 10 ± 3 × 10^−9^yr^−1^ (Supplementary Movies 17–19), and an increase in *T*_dyn_ from 25 ± 6 to 35 ± 7 MPa. Synextensional volcanic and sedimentary deposits within east-northeast, east-west, and west-northwest trending half-grabens of the Mina-Dyer region in Nevada indicate a N–S extension during Late Oligocene and Early Miocene^50^, consistent with our lithospheric deviatoric extension directions during this time interval (Figs. 9d, f, and 10f). The increase in $\bar \eta$ occurred during a time when there is less magmatic activity^8,35^ within eastern Nevada and the upper mantle temperature model there shows a decrease from ~1370 to ~1330 °C (Fig. 9d, e). Our calculations for a non-molten upper mantle phase (*M*_ext_ > *M*_0_), with increasing values of *λ*_f_ and decreasing values for *P*_f_, *C*_OH_, and strain rates, demonstrate a viscosity hardening, which we interpret to be associated with the cooling of underplated mafic intrusions^51^ at the base of the crust during 31–25 Ma. Following this, however, our model shows a transition to a molten phase for upper mantle (*M*_ext_ < *M*_0_), which is in agreement with andesitic magmatism during 25–22 Ma^48^, and this is followed by a period of high-strain rates and increased *C*_OH_ that begins at 21 Ma (Fig. 10q–t).

Garnet–pyroxenite xenoliths support the presence of a thick crust and high elevation in central Arizona at least until 25 Ma^52^. We show that compressive boundary stresses across Mogollon Highlands drop dramatically when the EPR contacts and evolves along the trench margin (during 31–22), resulting in crustal stretching and topographic collapse. As such, *T*_GPE_/*T*_BCS_ increases from 2.5 to 6, *E* increases from 5 ± 2 to 40 ± 10 × 10^−9^ yr^−1^, $\bar \eta$ decreases from 10^23.2^ ± 10^22.2^ to 10^22^ ± 10^21.2^ Pa s, and *T*_dyn_ decreases from 35 ± 5 during extension initiation to 15 ± 4 MPa at the conclusion of collapse (Figs. 7g, h and 9d). The NE–SW tensional oriented deviatoric stresses within Mogollon Highlands during topographic collapse is in good agreement with key geologic stretch directions obtained from vergence of Cordilleran MCCs^1^, Miocene fault trends^53^, and Miocene dyke trends^54^ (Fig. 10b, f). A naturally constrained stress profile through the middle crust for the footwall of the Whipple Mountains MCC^55^ indicates a minimum depth-averaged strength profile of >15 MPa for the entire lithosphere (100 km thickness) during Miocene extension there, in agreement with our model stress values of >15 MPa (Fig. 10f). We have tested the possibility of a much lower topography (lower GPE) in our model geodynamic estimates during the extensional collapse. In the absence of Mogollon Highlands at the time of extension initiation, the model fails to converge, the distribution of the strain rates fails to match expected stretch distributions, and final deviatoric stress magnitudes are too low (5 MPa or less) (Supplementary Note 6 and Supplementary Fig. 5). Our dynamic results, therefore, show that the Mogollon Highlands, supported by a thick crustal root, are required to explain these stress magnitudes^55^ and strain orientations along the belt of MCCs from southeast to northwest Arizona (Fig. 10f). While *T*_GPE_/*T*_BCS_ increases dramatically during the initial phase of collapse and MCC development, it is not enough to explain the initiation of high extensional strain rates, which also require an order of magnitude reduction in effective viscosity. Our results show that such a weakening is caused by a molten phase (*M*_ext_ < *M*_0_) and dry to wet partial melting of upper mantle during ~35–27 Ma^56^, associated with an increase for upper mantle temperature from ~1290 to ~1350 °C, decrease of *λ*_*m*_ from 0.07 to 0.04, and an increase in log_10_*C*_OH_ from −1 to 1. The viscosity reduction following 27 Ma is associated with a wet partial melting of the lower crust as the upper mantle is determined to be in a non-molten phase. Felsic and mafic volcanics within the belt of extensional collapse from southeast to northwest Arizona^35,42,56^ support these influences on viscosity reduction (Fig. 10i–l).

During Early Miocene (22–16 Ma), coinciding with the northwest migration of the MTJ and associated reduction of compressional BCSs, along with the expansion of the slab window, there is a reduction for $\bar \eta$ in eastern California, central and southern Nevada, western Utah, as well as western Arizona from 10^22.6^ ± 10^21.4^ to 10^21.6^ ± 10^21^ Pa s. This viscosity reduction is accompanied by an increase of *E* from 15 ± 4 to 45 ± 15 × 10^−9^ yr^−1^ in southern Nevada, which results in NE-SW topographic collapse of the northwestern portion of Mogollon Highlands. The decrease in GPE leads to a drop in *T*_dyn_ from 26 ± 5 to 16 ± 4 MPa (Figs. 9d, g, f, i and 10f, g). The change in orientation of extensional deviatoric stresses from N–S to NE–SW during Early Miocene in our geodynamic models is in agreement with evidence that show the Late Oligocene N–S fault slip lineations are cut by younger E–NE trending Miocene fault slip lineations in central-eastern Nevada^50,53^ (Figs. 9d, f, g, I and 10f, g). Based on our results, the reduction of $\bar \eta$ is associated with a molten phase (*M*_ext_ < *M*_0_) and dry to wet partial melting of upper mantle^48^ as temperature increases from 1330 to 1370 °C, *λ*_*m*_ decreases from 0.1 to 0.055, and log_10_*C*_OH_ increases from −2.5 to 0 (Fig. 10m–p).

During Middle Miocene (16–13 Ma), there is a renewed crustal thinning and topographic collapse of the entire Nevadaplano (second phase) and development of MCCs within western Arizona and southern Nevada (Fig. 9g, i) as the MTJ migrates from 36° to 37.5°N. The ENE–WSW extensional orientation of dynamic deviatoric stresses agrees with the stretch direction of MCCs^55^ and Miocene fault trends^53^ (Fig. 10c, g). This renewed collapse of the Nevadaplano results in a reduction of *T*_dyn_ from 25 ± 6 to 16 ± 4 MPa along with a gradual increase in *E* from 50 ± 15 to 80 ± 20 × 10^−9^ yr^−1^ and development of a zone of weakened effective viscosity of ~10^21.5^ ± 10^21^ Pa s (Fig. 9g). We interpret this viscosity weakening to be the result of a molten phase (*M*_ext_ > *M*_0_) and dry to wet partial melting for upper mantle^48^ inferred from increase of upper mantle temperature from 1320 to 1360 °C, decrease of *λ*_f_ from 0.06 to 0.03, and an increase of log_10_*C*_OH_ from −2.5 to −0.5 (Fig. 10q–t). In contrast to the Nevadaplano region, south-central Arizona undergoes effective viscosity increase from 10^22^ ± 10^21.2^ to 10^23^ ± 10^21.6^ Pa s, as *E* decreases from 50 ± 8 to 10 ± 2 × 10^−9^ yr^−1^, and *T*_dyn_ increases from 5 ± 2.5 to 15 ± 3 MPa. This viscosity hardening is associated with a non-molten phase (*M*_ext_ > *M*_0_) and cooling of underplated mafic intrusions as *λ*_*f*_ increases from 0.01 to 0.05 and log_10_*C*_OH_ decreases from 1 to −2 and is in direct relationship with reduction of magmatism and upper mantle temperature within south-central Arizona (Fig. 9g, h and 10i–l).

The dynamic modeling demonstrates that the opening of the Rio Grande Rift (RGR) is influenced by both the evolution of the boundary conditions and the weakening of the lithosphere. Prior to 20 Ma the MTJ lies south of 35°N and strong lithosphere in southern Nevada and the Colorado Plateau acts as stress guide, transmitting contractional stresses related to the accommodation of Farallon-North America relative motion across the RGR region, suppressing extension there (Figs. 7d and 9f). However, between 20 and 15 Ma, the MTJ migrates to ~37 °N, southern Nevada weakens, and subduction related stresses are no longer transmitted across the Colorado Plateau (Figs. 7e, f and 9i). This causes the compressional stresses to drop away across the RGR along its entire length, where *T*_GPE_/*T*_BCS_ jumps to ~3 along the rift zone (Fig. 7e, h), followed by a transition to extensional BCS along the entire RGR by ~15 Ma (Fig. 7f). This transition to extension is timed with the collision of the EPR at the trench along northern Mexico after 19 Ma (Fig. 7e, f, h, i). This change in BCS coincides with the initiation of E–W extension in highlands (high GPE) of northern Mexico, east of the Sierra Madre Occidental (Chihuahua Highlands). Therefore, the change in BCS, together with the existing high GPE, yields a simultaneous opening along the entire length of the RGR from southern Colorado down to Chihuahua Highlands, consistent with thermochronological results arguing for synchronous opening of the RGR^57^. This results in westward velocities of the southern Basin and Range Province that increase in magnitude towards the south, reaching values of ~10 mm yr^−1^ westward relative to the North America at 28°N in northern Mexico (Supplementary Movies 20 and 21). The westward driven collapse in northern Mexico continues between 19 and 4 Ma (Figs. 7e, f, h, i and 9i).

The Late Miocene time interval (13–9 Ma) is characterized by the development of two fundamentally new deformation zones. First, southern Arizona develops a broad right-lateral shear zone as Northern Mexico opens up E–W^11^. Second, there is a transition from NE–SW extension in southern Nevada and eastern California to a zone of transtensional shear in a through-going Eastern California Shear Zone (ECSZ)^58^ with E–W extension (Figs. 9i, l and 10d, g, h). The W–NW motion of northern Mexico generates a distributed right-lateral shear zone across southern Arizona. This shear zone, generated by body forces and plate motion boundary conditions, is a controversial feature in the kinematic reconstruction of McQuarrie and Wernicke^11^. We propose a mechanism that may suggest that the Southern Arizona Shear Zone is a likely outcome based on the evolution of the history of EPR interaction with the trench. That is, following the collapse of the Mogollon Highlands, central Arizona experienced a gradual viscosity hardening between 18 and 13 Ma. However, during the same time interval, terranes to the south within northern Mexico are extending rapidly, owing to E–W extensional influence of Pacific-North America boundary conditions and the high GPE distributions in the Chihuahua Highlands. As central Arizona became progressively more rigid and block-like, a shear zone would develop in southernmost Arizona to accommodate the relative motions between the edges of the central Arizona block and the rapidly moving regions in northern Mexico (Fig. 9j, l). The development of this shear zone coincides with the reduction of $\bar \eta$ in our geodynamic model, where *T*_dyn_ also decreases from 25 ± 3.5 to 13 ± 3 MPa and *E* increases from 5 ± 1 to 30 ± 5 × 10^−9^yr^−1^ (Fig. 9j). The causal mechanism for this viscosity variation is related to a molten phase (*M*_ext_ < *M*_0_) and dry to wet partial melting of upper mantle as *λ*_m_ decreases from 0.05 to 0.01 and log_10_*C*_OH_ increases from −2.2 to −0.2, which agrees with mafic (basaltic) and intermediate magmatism that occurred within central and southern Arizona during this time interval^35,56^ (Fig. 10i–l). The ~40–45° clockwise rotation of extensional stresses in eastern California and southern Nevada (17–13 Ma) is caused by the breakup of topography and collapse of the Nevadaplano (change in GPE related stresses) (Fig. 7b, c), and is evident by the change in Miocene fault trends within southern Nevada (Fig. 10c, d). From 13 to 9 Ma the change from pure extension to transtension in southern Nevada and ECSZ is associated with a change in Pacific Plate’s pole of rotation^6,59^. During this time interval, southern Nevada undergoes viscosity hardening as upper mantle transitions from molten to non-molten phase (increase of *λ*_f_ from −0.01 to 0.06 and reduction of log_10_*C*_OH_ from 0.5 to −2) (Fig. 10g, h, m–p), consistent with low water content in upper mantle lithosphere inferred from xenoliths^60^. With the migration of the MTJ, the shear zone in eastern California weakens and propagates further north. As such, the upper mantle temperature increases there from ~1300 to ~1400 °C, and upper mantle is consistently in a molten phase (*M*_ext_ < *M*_0_) with wet partial melting^48^, which explains the development of lower effective viscosities in the ECSZ (Fig. 9j, k).

Our dynamic models also show that the opening of the Gulf of California at ~6 Ma is caused by two primary developments during Late Miocene. First, excess GPE in the Chihuahua Highlands is dissipated by 5 Ma and extension there stops. Second, the southern Arizona lithosphere effective viscosity hardens (increase of $\bar \eta$ from 10^21.8^ ± 10^21.2^ to 10^23.2^ ± 10^21.8^ Pa s) (Fig. 9m–o). These two factors cause all transtensional relative motion between Pacific and North America to become focused adjacent to the margin within what is now the Gulf of California region, enabling the gradual transfer of the coastal fragment of California to the Pacific plate where extensional faulting continued during Pliocene to the present^61^.

## Discussion

This work presents for the first time a complete geodynamic model that pulls all of the key elements together to quantify the sources and primary driving factors for deformational features in southwestern North America since Late Eocene. We show through benchmarking, blind tests, that if body forces, plate motion boundary conditions, and surface strain rate magnitudes are known through time, then it is possible to accurately recover depth-integrated deviatoric stresses and effective viscosity (Figs. 2–5). Although we use the kinematic model of McQuarrie and Wernicke^11^ to reconstruct paleo-topography and paleo-crustal structure, the dynamic model that we investigate depends on the distributions of the internal body forces derived from these estimates of paleo-topography and paleo-crustal thicknesses, along with external velocity boundary conditions; there is no guarantee that such a dynamic model can reproduce the strain history of McQuarrie and Wernicke^11^. For example, we demonstrate the importance of Mogollon Highlands by arguing that even with the same velocity boundary conditions and the same scalar strain rates (*E*) from McQuarrie and Wernicke^11^, a different paleo-topography setting (different internal body force distribution) in Arizona fails to properly reproduce the strain history there and also fails to satisfy natural sample-based lithospheric strength estimates^55^ at the time of extension. Therefore, comparison of dynamic model output (strain orientations and internal relative motions) with the kinematic model (Fig. 10) is a viable means for evaluating the potential accuracy of the history of reconstructed topography and lithospheric effective viscosities.

The modeling performed here is based on the assumption that the scale of differential motion between upper and lower extending lithosphere in southwestern North America was limited to isolated regions^62^. This is justified by the great aspect ratio of orogen width to lithospheric thickness^63,64^, along with the need of matching far-field boundary conditions for plate motions. The latter condition leads to the model of free-boundary collapse of over-thickened crust^62^, which predicts limited differential motion between upper brittle and weak lower crust. Hence, while lower crustal flow was likely involved during the stretching process^65^, such a lower crustal flow must be limited to the regions containing crustal welts. Further evidence of the limited differential motion between upper and lower lithosphere is indicated by the strong correlation between the reconstructed stretching history and the distribution of upper mantle seismic wave speeds and reconstructed positions of volcanism^35^ (Fig. 6).

The similarity between our stress boundary conditions with global mantle flow coupling results for present-day^46,66^ within Basin and Range Province (Fig. 8) implies that, whereas basal tractions integrate over very long distances to become important in global models^46,66^, smaller-scale convection (slab rollback) is unlikely to have had a significant impact on stress field evolution in southwestern North America through its introduction of tractions to the base of the lithosphere. However, the temporal and spatial correlation between magmatism and lowering of lithosphere effective viscosity in our model suggests that slab rollback likely played a critical role in lithosphere weakening through the introduction of heat, melts, and fluids (Fig. 9). Our results show that rheological hardening within the Nevadaplano region (31–22 Ma), south-central Arizona (18–13 Ma), southern Nevada and ECSZ (13–9 Ma), and southern Arizona (8–3 Ma) is associated with the cooling of previously underplated mafic intrusions at the base of the crust^51^. This cooling occurs a few million years after active magmatism has migrated away from regions previously occupied by paleo-highlands (Figs. 9 and 10i–t). While slab rollback was likely critical in the strength evolution of the lithosphere in southwestern North America, our results show that body forces as well as the progressive change in boundary conditions were the major factors dictating the style of strain history and topographic collapse from Late Eocene to Early Miocene. Boundary conditions associated with the development of the SAFS were increasingly important from Middle Miocene to the present where GPE was greatly diminished following the collapse of the Nevadaplano and Mogollon Highlands (Fig. 7).

## Methods

### Estimating the depth integrals of deviatoric stresses

We investigate the mechanisms and forces responsible for driving the long-term deformation of the continental lithosphere of southwestern North America in space and time by assuming that the lithospheric mantle is involved in the crustal stretching process^67^ as opposed to a crust that is mechanically decoupled from mantle lithosphere^21,68^. We solve the depth-integrated 3-D spherical form of the force-balance equations^18,32,33^ for depth integrated horizontal deviatoric stresses. The cartesian form of these equations is

1 $$\frac{{\partial \sigma _{ij}}}{{\partial x_j}} + {\rho} g_i= 0,$$

where *σ*_*ij*_ is the stress tensor, *g* is the acceleration of gravity, and *ρ* is density, and with summation notation, *i* and *j* = *x*, *y*, and *z*. Vertical integration of the force-balance equations, using summation notation, results in the following

2 $$-\frac{{\partial \bar \sigma _{zz}}}{{\partial x_\alpha }} = \frac{\partial }{{\partial x_\beta }}\left( {\bar \tau _{\alpha \beta } + \delta _{\alpha \beta }\bar \tau _{\gamma \gamma }} \right),$$

where $\bar \sigma$_*zz*_ is the depth-integrated vertical stress or GPE per unit area, $\bar \tau _{\alpha \beta }$ is the depth-integrated horizontal deviatoric stress tensor, $\bar \tau _{\gamma \gamma } = \left( {\bar \tau _{xx} + \bar \tau _{yy}} \right) = - \bar \tau _{zz}$, and $\delta _{\alpha \beta }$ is the Kronecker delta. Eq. (2) assumes that horizontal gradients of the vertical integrals of shear stress (basal tractions), $\bar \tau _{{xz}}$ and $\bar \tau _{{yz}}$, are small in comparison with the product of acceleration of gravity and density

3 $$\frac{\partial }{{\partial x}}\mathop {\smallint }\nolimits_{\!\!\! - h}^z \tau _{xz}\left( z \right)dz + \frac{\partial }{{\partial y}}\mathop {\smallint }\nolimits_{\!\!\! - h}^z \tau _{yz}(z) dz \, \, < < \mathop {\smallint }\nolimits_{\!\!\! - h}^z \rho (z)gdz.$$

We also ignore the contribution of basal tractions ($\bar \tau _{{xz}}$ and $\bar \tau _{{yz}}$) applied at the base of the lithosphere below deforming regions, which, for example, can be associated with lithospheric coupling with mantle flow. Although these basal tractions integrate over very long distances to become important in global models^46,66^, the regional distances involved in our deforming areas are small enough such that the contribution of basal tractions associated with smaller-scale convection on the stress field are expected to be negligible. For example, we show that our boundary condition solutions for present-day strongly resemble the globally computed stress fields generated by basal tractions associated with global mantle flow coupling with the lithosphere^57^. This comparison suggests that the stress response to our applied velocity boundary conditions approximates this global influence of coupling over long distances, but we argue here that ignoring the influence of horizontal tractions directly below the deforming regions should have a minimal effect on the stress field (Supplementary Fig. 3).

From the approximation in Eq. (3), the vertical stress at depth *z*, *σ*_*zz*_(*z*), is equal to

4 $$\sigma _{zz} = \mathop {\int}\nolimits_{\!\!\!\!\! - h}^z \rho (z)gdz.$$

The depth integrated vertical stress within the lithosphere ($\bar \sigma _{zz}$), also known as GPE, is expressed as

5 $$\bar \sigma _{zz} = - {\mathrm{g}}\mathop {\int}\nolimits_{\!\!\!\!\! - h}^L {\left[ {\mathop {\int}\nolimits_{\!\!\! - h}^z \rho (z^{\prime} )dz^{\prime} } \right]} dz = \mathop {\int}\nolimits_{\!\!\!\! - h}^L {(L - z)} \rho (z)gdz,$$

where *h* is elevation above the sea level, *L* is the maximum depth of integration, representing the reference level and approximating the base of the lithosphere (100 km below sea level), and *z* is a variable of integration. The integral of this vertical stress includes the influence of changes in topography associated with density variations for lithospheric mantle and crustal thicknesses^8^ through time. The key effective body force term in Eq. (2) within the deforming region involves the spatial gradients in GPE ($\bar \sigma _{zz}$). We calculate GPE values for each 0.5 Myr time interval using a constant crustal density of 2700 kg m^−3^, and a variable time-dependent upper mantle density that accounts for an evolving temperature field^8^ and the evolving crustal thicknesses and paleo-elevations of Bahadori et al.^8^. GPE values are calculated at a resolution of 0.1° × 0.1° (latitude: *θ*, longitude: $\emptyset$), and averages of these are computed within each 0.5° × 0.5° area inside our region-wide finite element grid containing 2376 cells for southwestern North America for all 0.5 Myr time increments (*k* = 1–73, where 1 and 73 denote present-day and 36 Ma, respectively) (Supplementary Fig. 6 and Supplementary Movies 4 and 5)

6 $$\sigma _{zz}(z_i)_{k_{(\theta ,\emptyset )}} = P(z_i)_{k_{(\theta ,\emptyset )}} = \mathop {\sum }\limits_{i = 0}^{i = n} g\mathop {\smallint }\nolimits_{\!\!\! z_i}^{z_{i + 1}} \rho \left( {z_{i + 1}} \right)_{k_{(\theta ,\emptyset )}}(dz_{i + 1})_{k_{(\theta ,\emptyset )}},$$

where the subscript *i* = 0, 1, 2 respectively denotes sea level (*i* = 0, *dz*_*1*_ = *h*), Moho depth (*i* = 1, *dz*_*2*_ = *H* − *h*, where *H* is the crustal thickness, and *h* is the elevation) and 100 km depth (*i* = 2, *dz*_*3*_ = 100 − *H* + *h*).

The overburden pressure at 100 km depth below sea level, *P*(*z*_3_), is calculated by summation of pressure at Moho depth from crustal thickness

7 $$P(z_1)_{k_{(\theta ,\emptyset )}} = g\rho _c\left( {z_2} \right)_{k_{(\theta ,\emptyset )}}(dz_2)_{k_{(\theta ,\emptyset )}},$$

and pressure from lithospheric mantle thickness at 100 km depth

8 $$P(z_2)_{k_{(\theta ,\emptyset )}} = g\rho _{m^{\prime} }\left( {z_3} \right)_{k_{(\theta ,\emptyset )}}(dz_3)_{k_{(\theta ,\emptyset )}},$$

as

9 $$P(z_3)_{k_{(\theta ,\emptyset )}} = P(z_1)_{k_{(\theta ,\emptyset )}} + P(z_2)_{k_{(\theta ,\emptyset )}}.$$

Thus, the time-dependent GPE values ($\bar \sigma _{zz}$) involve the vertical integrals of pressure, $P(z_i)_{k_{(\theta ,\emptyset )}}$, calculated as

10 $$({\mathrm{GPE}})_{k_{(\theta ,\emptyset )}} = \frac{1}{2}[P(z_1)_{k_{(\theta ,\emptyset )}}.H_{k_{(\theta ,\emptyset )}}] + \frac{1}{2}[P(z_1)_{k_{(\theta ,\emptyset )}} \\ + \, P(z_2)_{k_{(\theta ,\emptyset )}}].(10000 + h_{k_{(\theta ,\emptyset )}} - H_{k_{(\theta ,\emptyset )}}),$$

where $H_{k_{(\theta ,\emptyset )}}$ and $h_{k_{(\theta ,\emptyset )}}$ are time-dependent crustal thicknesses and paleo-elevations through time (Supplementary Movies 4 and 5 and Supplementary Data 2–4). The GPE values computed for each time step are used in our force-balance solution treatment, described below.

### Parameterization of the iterative dynamic model

We use method of Flesch et al.^18,32,33^ to refine our geodynamic calculations from surface to 100 km below sea level in southwestern North America with a defined distribution of horizontal gradients of depth integrated vertical deviatoric stresses (GPE per unit area), lateral variations in depth integrated effective viscosity of the lithosphere, and velocity boundary conditions relative to the North American plate. We solve the force-balance equations in (2) through the optimization of the functional^69^

11 $$\Theta = \smallint \smallint _s[D + \dot \varepsilon _{\gamma \gamma }\bar \sigma _{zz}]dxdy - \smallint _{\partial S}v_\alpha \bar \sigma _{zz}n_\alpha dl,$$

where *D* is the dissipation potential, $\bar \sigma _{zz}$ is GPE, and *v*_*a*_ is the velocity boundary conditions of the relative motions of Pacific- and Farallon-North America plates applied around the grid area boundary $(\partial S)$ with normal vector *n*_*α*_. The dissipation potential (*D*) is a function of strain rates, rheological parameter (*B*), and the power law exponent (*n*)^33^

12 $$D = \frac{n}{{n + 1}}B(\dot \varepsilon _{\alpha \beta }\dot \varepsilon _{\alpha \beta } + \dot \varepsilon _{\gamma \gamma }\dot \varepsilon _{\gamma \gamma })^{\frac{{n + 1}}{{2n}}},$$

where $\dot \varepsilon _{\gamma \gamma } = \left( {\dot \varepsilon _{xx} + \dot \varepsilon _{yy}} \right) = - \dot \varepsilon _{zz}$. The effective viscosity $\bar \eta = BE^{1/n - 1}$, where *B* is the rheological parameter that is sensitive to temperature^63,64^, *n* is the power-law exponent, and *E* is the second invariant of strain rate. *E* and *T* are calculated as

13 $$E = \sqrt {\dot \varepsilon _{xx}^2 + \dot \varepsilon _{yy}^2 + \dot \varepsilon _{zz}^2 + \dot \varepsilon _{xy}^2 + \dot \varepsilon _{yx}^2} = \sqrt {2\dot \varepsilon _{xx}^2 + 2\dot \varepsilon _{yy}^2 + 2\dot \varepsilon _{xx}\dot \varepsilon _{yy} + 2\dot \varepsilon _{xy}^2} ,$$

14 $$T = \sqrt {\tau _{xx}^2 + \tau _{yy}^2 + \tau _{zz}^2 + \tau _{xy}^2 + \tau _{yx}^2} = \sqrt {2\tau _{xx}^2 + 2\tau _{yy}^2 + 2\tau _{xx}\tau _{yy} + 2\tau _{xy}^2}.$$

We arrive at the optimal solution, involving minimization of (11), iteratively. We first solve the force-balance equations associated with GPE distributions alone, assuming constant viscosity and zero velocity boundary conditions in (11) along a rigid boundary. The deviatoric stress solution, obtained through minimization of (11), depends only on the gradient in GPE and is invariant to the magnitude of the constant value for initial effective viscosity. This solution provides initial estimates of the distribution of deviatoric stresses that balance gradients of GPE (Supplementary Figs. 6 and 7). We then estimate an initial distribution of *B*, using *n* = 1 and $\bar \eta = B = \frac{T}{E}$, where *E* is the second invariant of strain rates (scalar values) from the kinematic solution. Using this laterally varying first estimate of the distribution of *B*, we seek the complete solution by solving the force-balance through minimization of (11) with respect to model strain rates $\dot \varepsilon _{\alpha \beta }$, using the distribution of GPE and velocity boundary conditions that are appropriate for the given time interval. Model values of strain rate, multiplied by the previous iteration’s viscosity, provide updated deviatoric stresses, $\bar \tau _{\alpha \beta }$. Using the updated deviatoric stresses we calculate a new effective viscosity field and a new force-balance solution. The procedure is repeated until there are no further changes in *T* and model values of strain rate $\dot \varepsilon _{\alpha \beta }$ (Supplementary Figs. 1, 8–11, Supplementary Movies 9, 10, 17–21, and Supplementary Data 5–13). Through benchmarking, blind tests, we have found that if far-field velocity boundary conditions, GPE values (paleo-topography and crustal structure), and scalar values of kinematic strain rates (*E*) are known through time, then the absolute magnitudes of vertically averaged deviatoric stresses and effective viscosities within the lithosphere can be uniquely recovered using this iterative procedure involving solutions to the force-balance equations (Supplementary Fig. 2). This complete solution (Supplementary Fig. 10 and Supplementary Movie 10) can be thought of as the combination of stresses associated with GPE gradients and stresses that accommodate the relative plate motion. To understand the dynamics better, it is important to separate contributions in stress associated with GPE gradients from those associated with the accommodation of plate motions, which we term BCS. We therefore produce the BCS solution along with boundary condition strain rate tensors and velocities by solving the force-balance equations in (2) through the optimization of (11), using the final viscosity structure from complete solution and zero GPE gradients, $0 = - \frac{{\partial \bar \sigma _{zz}}}{{\partial x_\alpha }} = \frac{\partial }{{\partial x_\beta }}(\bar \tau _{\alpha \beta } + \delta _{\alpha \beta }\bar \tau _{\gamma \gamma })$, while applying the correct velocity boundary conditions of the relative motions of Pacific- and Farallon-North America plates around the grid area boundary $(\partial S)$ (Supplementary Figs. 3, 12–15, and Supplementary Movies 7 and 22–26). We also show that it is possible to recover similar BCS solution by redetermining the deviatoric stresses associated with GPE alone $(\bar \tau _{\alpha \beta }^\prime )_{{\mathrm{GPE}}}$ by solving Eq. (2) again, but by applying the final laterally varying estimates of effective viscosity (rather than the constant value we start with) (Supplementary Fig. 6 and Supplementary Movie 5). We then subtract the components of these new deviatoric stresses associated with GPE gradients from the complete solution. The residual constitutes the contribution in stresses associated with the accommodation of plate motion alone (Supplementary Figs. 3 and 12 and Supplementary Movie 7).

15 $$(\bar \tau _{\alpha \beta })_{{\mathrm{BCS}}} = (\bar \tau _{\alpha \beta })_{{\mathrm{dyn}}} - (\bar \tau _{\alpha \beta }^\prime )_{{\mathrm{GPE}}}.$$

Bahadori et al.^8^ have determined formal estimates for uncertainties in surface velocities, strain rates, crustal thickness, and surface elevation since Late Eocene. Using those uncertainties, and by performing a bootstrapping method with 100 iterations (100 random number and 100 realized GPE values), we first calculate the estimates for standard errors of GPE. Then, by performing the iterative dynamic model 100 times, we calculate the standard errors for stress tensors, the second invariant of stress, as well as standard errors for the effective viscosity of the lithosphere through time (Supplementary Figs. 6, 7, 9, 11, 16, Supplementary Notes 10–13, Supplementary Movies 27–31, and Supplementary Data 14–22).

### Computation of causal mechanisms for viscosity variations

To investigate the role of melts and fluids on lithosphere weakening and hardening that our geodynamic models show, we use a simplified approach using the method of Gerya and Meilick^37^. The geodynamically constrained viscosity variations, as mentioned above, are derived from solutions to force-balance equations, which depend on time-dependent GPE, boundary condition velocities, and scalar values of kinematic strain rates (*E*). Our time-dependent estimate of lithospheric pressure variations and upper mantle temperature changes enable us to approximate volumetric degree of melting $(M_0)_{k_{(\theta ,\emptyset )}}$ (latitude: *θ*, longitude: ∅, *k* = 0–72, where 0 and 72 denote 36 Ma and present-day, respectively) and total amount of extracted melt $(M)_{k_{(\theta ,\emptyset )}}$ for each 0.5 Myr time step as well as total previously extracted melt fraction $(\mathop {\sum}\nolimits_{k = 0}^{k = n - 1} {M_{\rm{ext}}} )_{k_{(\theta ,\emptyset )}}$ for upper mantle^36,37^ (*n* is the total number of time steps of 0.5 Myr). With this method we can define two phases of molten and non-molten upper mantle through time. The molten phase includes wet partial melting (andesitic magmatism) or dry partial melting (basaltic magmatism) for upper mantle. The non-molten phase includes hardening of lower crust (magmatic underplating) or wet partial melting of lower crust (rhyolitic magmatism). We consider an initial wet upper mantle in Late Eocene for our causal mechanism models based on evidence supporting the hydration of the base of the North American lithosphere by de-watering of Farallon slab during its Laramide flat subduction^39–42^. Our causal mechanism models for interpreting weakening and hardening of the lithosphere serve as an internal check of the consistency between our changing force-balance solutions (changes in pressure, topography, densities, effective viscosities, strain rates, and stresses) and a temperature model that we independently derive from upper mantle temperature changes associated with the history of magmatism^8^, along with constraints form upper mantle seismic velocities^34^.

The standard volumetric degree of melting *M*_0_ (without melt extraction) (Supplementary Fig. 17, Supplementary Movie 13, and Supplementary Data 23 and 24) is calculated as

16 $$(M_0)_{k_{(\theta ,\emptyset )}} = 0\, \, {\mathrm{if}} \,(T)_{k_{(\theta ,\emptyset )}} < \, (T_{{\mathrm{solidus}}})_{k_{(\theta ,\emptyset )}},$$

17 $$(M_0)_{k_{(\theta ,\emptyset )}} = \frac{{(T)_{k_{(\theta ,\emptyset )}} - (T_{{\mathrm{solidus}}})_{k_{(\theta ,\emptyset )}}}}{{(T_{{\mathrm{liquidus}}})_{k_{(\theta ,\emptyset )}} - (T_{{\mathrm{solidus}}})_{k_{(\theta ,\emptyset )}}}} \, {\mathrm{if}} \,(T_{{\mathrm{solidus}}})_{k_{(\theta ,\emptyset )}} < \, \, (T)_{k_{(\theta ,\emptyset )}} < \, \, (T_{{\mathrm{liquidus}}})_{k_{(\theta ,\emptyset )}},$$

18 $$(M_0)_{k_{(\theta ,\emptyset )}} = 1\,{\mathrm{if}}\,(T)_{k_{(\theta ,\emptyset )}} > \, \, (T_{{\mathrm{liquidus}}})_{k_{(\theta ,\emptyset )}},$$

where *T*_solidus_ is solidus temperature and *T*_liquidus_ is dry liquidus temperature at a given pressure and composition for upper mantle calculated as

19 $$(T_{{\mathrm{solidus}}}) = [1240 + 49800/((P)_{k_{(\theta ,\emptyset )}} + 323)] \, \, {\mathrm{for}} \, (P)_{k_{(\theta ,\emptyset )}} < \, \, 2400\,{\mathrm{MPa}},$$

20 $$(T_{{\mathrm{solidus}}})_{k_{(\theta ,\emptyset )}} = [1266 - 0.0118(P)_{k_{(\theta ,\emptyset )}} + 0.0000035(P)_{k_{(\theta ,\emptyset )}}^2] \, \, {\mathrm{for}} \, (P)_{k_{(\theta ,\emptyset )}} > \, \, 2400\,{\mathrm{MPa}},$$

21 $$(T_{{\mathrm{liquidus}}})_{k_{(\theta ,\emptyset )}} = [2073 + 0.114(P)_{k_{(\theta ,\emptyset )}}],$$

To calculate the extracted amount of melt from a partially molten rock^36,37^, we consider a melt extraction threshold $(M_{{\mathrm{max}}})_{k_{(\theta ,\emptyset )}} = 4\%$ and a non-extractable amount of melt $(M_{\rm{min}})_{k_{(\theta ,\emptyset )}} = 2\%$ that will stay in the source. We use the same rule to update our estimates of total amount of extracted melt (*M*) and total extracted melt fraction $(\mathop {\sum}\nolimits_{k = 0}^{k = n - 1} {M_{\rm{ext}}} )_{(\theta ,\emptyset )}$ through time starting at 36 Ma. The extracted amount of melt for each time step is calculated as^37^

22 $$(M)_{k_{(\theta ,\emptyset )}} = (M_0)_{k_{(\theta ,\emptyset )}} - \left( {\mathop {\sum}\limits_{k = 0}^{k = n - 1} {M_{\rm{ext}}} } \right)_{(\theta ,\emptyset )}$$

where *n* is the total number of time steps of 0.5 Myr. At 36 Ma we assume zero previously extracted melt fraction, e.g., $(\mathop {\sum}\nolimits_{37\,{\rm{Ma}}}^{36\,{\rm{Ma}}} {M_{{\rm{ext}}}} )_{(\theta ,\emptyset )} = 0$, and thus all of calculated $(M_0)_{k = n_{(\theta ,\emptyset )}}$ at 36 Ma (*n* = 0) will be extracted from source^36,37^. For the next time step at 35.5 Ma (*n* = 1), $(\mathop {\sum}\nolimits_{k = 0}^{k = n - 1} {M_{\rm{ext}}} )_{(\theta ,\emptyset )}$ is equal to $(M)_{k = n - 1_{(\theta ,\emptyset )}}$ calculated at 36 Ma, and we calculate new estimate of $(M_0)_{k = n_{(\theta ,\emptyset )}}$ based on equations of (16) to (21) and hence $(M)_{k = n_{(\theta ,\emptyset )}}$ based on Eq. (22) at 35.5 Ma. This new estimate of $(M)_{k = n_{(\theta ,\emptyset )}}$ will be added to $(\mathop {\sum}\nolimits_{k = 0}^{k = n - 1} {M_{\rm{ext}}} )_{(\theta ,\emptyset )}$ to update the total amount of melt already produced and extracted through time before 35 Ma (*n* = 2), and we continue this procedure for the whole southwestern portion of North America up to the present-day.

To determine the phase changes through time (molten versus non-molten), we follow the method of Gerya and Meilick^37^. Based on that method, a rock is considered non-molten or refractory when $(\mathop {\sum}\nolimits_{k = 0}^{k = n - 1} {M_{\rm{ext}}} )_{(\theta ,\emptyset )}$ is greater than $(M_0)_{k = n_{(\theta ,\emptyset )}}$ (because all the melt fraction gets extracted from the rock for a given temperature), and it is considered molten when $(\mathop {\sum}\nolimits_{k = 0}^{k = n - 1} {M_{\rm{ext}}} )_{(\theta ,\emptyset )}$ is less than $(M_0)_{k = n_{(\theta ,\emptyset )}}$. In the case of $(M)_{k = n_{(\theta ,\emptyset )}} > (M_{\rm{max}})_{k = n_{(\theta ,\emptyset )}}$, the extracted melt fraction $(M_{\rm{ext}})_{k = n_{(\theta ,\emptyset )}}$ for that time step is calculated as: $(M_{\rm{ext}})_{k = n_{(\theta ,\emptyset )}} = (M)_{k = n_{(\theta ,\emptyset )}} - (M_{{\mathrm{min}}})_{k = n_{(\theta ,\emptyset )}}$ and $(M_{\rm{ext}})_{k = n_{(\theta ,\emptyset )}}$ is added to $(\mathop {\sum}\nolimits_{k = 0}^{k = n - 1} {M_{\rm{ext}}} )_{(\theta ,\emptyset )}$ to update $(\mathop {\sum}\nolimits_{k = 0}^{k = n} {M_{\rm{ext}}} )_{(\theta ,\emptyset )}$ (Supplementary Fig. 18 and Supplementary Movie 15).

Using total amount of extracted melt $(M)_{k_{(\theta ,\emptyset )}}$, lithospheric pressure $(P)_{k_{(\theta ,\emptyset )}}$, and upper mantle temperature variation $(T)_{k_{(\theta ,\emptyset )}}$ from 36 Ma to present^8^ for each 0.5 Myr, we calculate the effective density of the partially molten upper mantle^37^ (Supplementary Fig. 19, Supplementary Movie 3, and Supplementary Data 25) as

23 $$(\rho _{_{{\mathrm{eff}}}})_{k_{(\theta ,\emptyset )}} = (\rho _{{\mathrm{solid}}})_{k_{(\theta ,\emptyset )}}\left[ {1-(M)_{k_{(\theta ,\emptyset )}} + (M)_{k_{(\theta ,\emptyset )}}\left( {\frac{{\rho _{0{\mathrm{molten}}}}}{{\rho _{0{\mathrm{solid}}}}}} \right)_{k_{(\theta ,\emptyset )}}} \right],$$

where *ρ*_0solid_ and *ρ*_0molten_ are the standard densities of solid (3200 kg m^−3^) and molten (2900 kg m^−3^) lithospheric mantle, respectively^37^, and *ρ*_solid_ is the density of solid lithospheric mantle at given pressure (MPa) and temperature (K), which is calculated as^37^

24 $$(\rho _{{\mathrm{solid}}})_{k_{(\theta ,\emptyset )}} = (\rho _{{\mathrm{0solid}}})_{k_{(\theta ,\emptyset )}} \times \{ 1-\alpha [(T)_{k_{(\theta ,\emptyset )}}-298]\} \times \{ 1 + \beta [(P)_{k_{(\theta ,\emptyset )}}-0.1]\},$$

where *α* and *β* are thermal expansion (3 × 10^−5^ K^−1^) and compressibility (1 × 10^−5^ MPa^−1^)^37^.

Our geodynamically constrained rheological model enables us to calculate melt pressure factor (*λ*_*m*_)^37^ for a molten upper mantle phase and fluid pressure factor (*λ*_*f*_)^37^ for a non-molten upper mantle phase through time (Supplementary Fig. 17, Supplementary Movie 14, and Supplementary Data 26). Here, we assume that both fluid and melt propagation can affect the rheology of upper mantle based on a Mohr–Coulomb criterion that limits the effective viscosity ($\bar \eta$) as follows

25 $$(\bar \eta )_{k_{(\theta ,\emptyset )}} \le \frac{{(T_{yield})_{k_{(\theta ,\emptyset )}}}}{{(E)_{k_{(\theta ,\emptyset )}}}},$$

where $(T_{\rm{yield}})_{k_{(\theta ,\emptyset )}} = \sqrt 2 (T)_{k_{(\theta ,\emptyset )}}$, and $(T)_{k_{(\theta ,\emptyset )}}$ is the second invariant of deviatoric stresses from iterative geodynamic model.

26 $$\sin (\varphi )_{k_{(\theta ,\emptyset )}} = \frac{{(T_{\rm{yield}})_{k_{(\theta ,\emptyset )}}-c}}{{(P)_{k_{(\theta ,\emptyset )}}}},$$

where *c* = 10 MPa is the cohesion based on experimentally determined flow laws^37^, *P* is lithospheric pressure (at 100 km depth), and *φ* is the internal friction angle^37^. Melt and fluid pressure reduce the yield strength of upper mantle. In the case of a molten phase for upper mantle $(M_{{\mathrm{ext}}} \, < \, M_0)_{k_{(\theta ,\emptyset )}}$ the change in melt pressure (*P*_*m*_) will control the variation in *λ*_*m*_ as

27 $$(\lambda _{\mathrm{m}})_{k_{(\theta ,\emptyset )}} = \frac{{sin(\varphi )_{k_{(\theta ,\emptyset )}}}}{{{\mathrm{sin}}(\varphi _{{\mathrm{dry}}})}} = 1 - \frac{{(P_m)_{k_{(\theta ,\emptyset )}}}}{{(P)_{k_{(\theta ,\emptyset )}}}} \,{\mathrm{if}} \, (M_{{\mathrm{ext}}} \, < \, M_0)_{k_{(\theta ,\emptyset )}},$$

where *φ*_dry_ = 0.1 is the experimentally determined friction angle of wet olivine^37^.

However, in the case of a non-molten phase for upper mantle $(M_{{\mathrm{ext}}} \, > \, M_0)_{k_{(\theta ,\emptyset )}}$, the change in fluid pressure (*P*_*f*_) will control the variation in *λ*_*f*_ as

28 $$(\lambda _{f})_{k_{(\theta ,\emptyset )}} = \frac{{\rm{sin}(\varphi )_{k_{(\theta ,\emptyset )}}}}{{{\mathrm{sin}}(\varphi _{{\mathrm{dry}}})}} = 1 - \frac{{(P_f)_{k_{(\theta ,\emptyset )}}}}{{(P)_{k_{(\theta ,\emptyset )}}}}\,{\mathrm{if}}\;(M_{{\mathrm{ext}}} \, > \, M_0)_{k_{(\theta ,\emptyset )}}.$$

To further investigate the role of water on effective viscosity variation of upper mantle and lower crust (wet or dry partial melting), we use a power law creep rheological model based on method of Dixon et al.^38^ to solve for variation of distributed water in nominally anhydrous minerals like olivine (*C*_OH_) (Fig. 9, Supplementary Fig. 18, Supplementary Movie 16, and Supplementary Data 27)

29 $$(\bar \eta )_{k_{(\theta ,\emptyset )}} = (\dot \varepsilon )_{k_{(\theta ,\emptyset )}}^{\left( {\frac{{1 - n}}{n}} \right)}A^{\frac{{ - 1}}{n}}(C_{\rm{OH}})_{k_{(\theta ,\emptyset )}}^{\frac{{ - r}}{n}}\left[ {{\mathrm{exp}}\left( { - \frac{{H + (P)_{k_{(\theta ,\emptyset )}}V}}{{R(T)_{k_{(\theta ,\emptyset )}}}}} \right)} \right]^{\frac{{ - 1}}{n}}10^6,$$

30 $$(C_{\rm{OH}})_{k_{(\theta ,\emptyset )}}^{\frac{{ - r}}{n}} = \frac{{(\bar \eta )_{k_{(\theta ,\emptyset )}}}}{{(\dot \varepsilon )_{k_{(\theta ,\emptyset )}}^{\left( {\frac{{1-n}}{n}} \right)}A^{\frac{{-1}}{n}}\left[ {{\mathrm{exp}}\left( { - \frac{{H + (P)_{k_{(\theta ,\emptyset )}}V}}{{R(T)_{k_{(\theta ,\emptyset )}}}}} \right)} \right]^{\frac{{ - 1}}{n}}10^6}},$$

where $\dot \varepsilon$ is the dynamically constrained strain rate, $\bar \eta$ is the effective viscosity, *P* is pressure, *T* is temperature, *H* is the activation enthalpy, *V* is activation volume, *R* is gas constant, *r* is fugacity exponent, and *n* is stress exponent. We use experimentally determined *n* = 3.5, *r* = 1.2, *V* = 11 × 10^−6^ m^3^ mol^−1^, *H* = 4,80,000, *R* = 8.314, and *A* = 90 (MPa) for a wet dislocation creep^38^.

For the molten upper mantle phase, we use the time-dependent trend in *λ*_*m*_ and *C*_OH_ to interpret whether the upper mantle has undergone weakening by wet partial melting (increasing trend in *C*_OH_ and *P*_*m*_ as well as decreasing trend in *λ*_*m*_) or dry partial melting (decreasing trend in *C*_OH_ and *P*_*m*_ as well as increasing trend in *λ*_*m*_) (Fig. 10). For the non-molten upper mantle phase, we use the time-dependent trend in *λ*_*f*_ and *C*_OH_ to interpret whether the lower crust has undergone hardening by cooling of underplated mafic intrusions or weakening by wet partial melting. During the time intervals in which *λ*_*f*_ is decreasing and *C*_OH_ is increasing, our interpretation is that the lower crust has undergone weakening due to wet partial melting (Fig. 10). During the time intervals in which *λ*_*f*_ is increasing and *C*_OH_ is decreasing, our interpretation is that the lower crust has undergone hardening due to either a dehydration process or cooling of underplated dense mafic intrusions^51^ (Fig. 10).

## Supplementary information

Peer Review File

Supplementary Information

Description of Additional Supplementary Files

Supplementary Data 1

Supplementary Data 2

Supplementary Data 3

Supplementary Data 4

Supplementary Data 5

Supplementary Data 6

Supplementary Data 7

Supplementary Data 8

Supplementary Data 9

Supplementary Data 10

Supplementary Data 11

Supplementary Data 12

Supplementary Data 13

Supplementary Data 14

Supplementary Data 15

Supplementary Data 16

Supplementary Data 17

Supplementary Data 18

Supplementary Data 19

Supplementary Data 20

Supplementary Data 21

Supplementary Data 22

Supplementary Data 23

Supplementary Data 24

Supplementary Data 25

Supplementary Data 26

Supplementary Data 27

Supplementary Movie 31

Supplementary Movie 1

Supplementary Movie 2

Supplementary Movie 3

Supplementary Movie 4

Supplementary Movie 5

Supplementary Movie 6

Supplementary Movie 7

Supplementary Movie 8

Supplementary Movie 9

Supplementary Movie 10

Supplementary Movie 11

Supplementary Movie 12

Supplementary Movie 13

Supplementary Movie 14

Supplementary Movie 15

Supplementary Movie 16

Supplementary Movie 17

Supplementary Movie 18

Supplementary Movie 19

Supplementary Movie 20

Supplementary Movie 21

Supplementary Movie 22

Supplementary Movie 23

Supplementary Movie 24

Supplementary Movie 25

Supplementary Movie 26

Supplementary Movie 27

Supplementary Movie 28

Supplementary Movie 29

Supplementary Movie 30

## Data Availability

The authors declare that all data supporting the findings of this study (e.g. figures, tables, movies) are available within the manuscript and its Supplementary Information files. The full database for geodynamic modeling solutions (model parameter outputs) and their associated estimates of errors presented in Figs. 1–10, Supplementary Figs. 1–19, and Movies 1–31 are available in Supplementary Data files 1–27.

## Supplementary information

**Supplementary information** is avaliable for this paper at 10.1038/s41467-019-12950-8.

## Code availability

Geodynamic modeling codes for producing force-balance solutions, refined velocity fields, strain rates, deviatoric stress fields, and effective viscosity of the lithosphere are freely available from the corresponding author upon request.
