# Supplementary material for: Geodynamic evolution of southwestern North America since the Late Eocene
Source: Nat Commun. 2019 Nov 18;10:5213. doi: 10.1038/s41467-019-12950-8 (PMC6861285; doi:10.1038/s41467-019-12950-8)
Supplement: Supplementary file 3 — Description of Additional Supplementary Files [file 41467_2019_12950_MOESM3_ESM.pdf]

Description of Additional Supplementary Files

Supplementary Data 1: includes kinematic horizontal velocities.

Each cell includes 7 columns:

column 1: Longitude
column 2: Latitude
column 3: x component of velocity vector with unit of mm/yr
column 4: y component of velocity vector with unit of mm/yr
column 5: standard error for x component of velocity vector with unit of mm/yr
column 6: standard error for y component of velocity vector with unit of mm/yr
column 7: correlation coefficient between x and y components of velocity vector

---

Supplementary Data 2: includes crustal thicknesses and their associated standard errors from Bahadori et al. (2018).

Each cell includes 4 columns:

column 1: Longitude
column 2: Latitude
column 3: crustal thickness (km) from Bahadori et al. (2018)
column 4: standard error for crustal thickness (km) from Bahadori et al. (2018)

Supplementary Data 3: includes surface elevations and their associated standard errors from Bahadori et al. (2018).

Each cell includes 4 columns:

column 1: Longitude
column 2: Latitude
column 3: surface elevations (m) from Bahadori et al. (2018)
column 4: standard error for surface elevations (m) from Bahadori et al. (2018)

Supplementary Data 4: includes Gravitational Potential Energy (GPE) estimates for the lithosphere.

Each cell includes 3 columns:

column 1: Longitude
column 2: Latitude
column 3: gravitational potential energy (GPE) (kg.m/s^2) from surface to 100 km depth

Each cell includes include 3 columns:

column 1: Longitude
column 2: Latitude
column 3: standard error for gravitational potential energy (GPE) (kg.m/s^2) from surface to 100 km depth

---

Supplementary Data 5: includes deviatoric stress tensors associated with GPE gradients achieved by solving the force-balance equations.

Each cell includes 5 columns:

column 1: Longitude
column 2: Latitude
column 3: depth integrated horizontal deviatoric stress tensor (txx) associated with GPE differences in unit of Newton/meter
column 4: depth integrated horizontal deviatoric stress tensor (tyy) associated with GPE differences in unit of Newton/meter
column 5: depth integrated horizontal deviatoric stress tensor (txy) associated with GPE differences in unit of Newton/meter

---

Supplementary Data 6: includes the second invariant of stresses associated with GPE gradients and their associated standard errors.

Each cell includes 3 columns:

column 1: Longitude
column 2: Latitude
column 3: second invariant of deviatoric stresses associated with GPE differences (MPa)

Each cell includes 3 columns:

column 1: Longitude
column 2: Latitude
column 3: standard errors for the second invariant of deviatoric stresses associated with GPE differences (MPa)

Supplementary Data 7: includes model dynamic horizontal velocities.

Each cell includes 7 columns:

column 1: Longitude
column 2: Latitude
column 3: x component of dynamic velocity vector with unit of mm/yr
column 4: y component of dynamic velocity vector with unit of mm/yr
column 5: standard error for x component of dynamic velocity vector with unit of mm/yr
column 6: standard error for y component of dynamic velocity vector with unit of mm/yr
column 7: correlation coefficient between x and y components of dynamic velocity vector

---

Supplementary Data 8: includes dynamic strain tensors and their associated standard errors.

Each cell includes 5 columns:

column 1: Longitude
column 2: Latitude
column 3: dynamic strain tensor (exx) (need to multiply by 10^-9/yr)
column 4: dynamic strain tensor (eyy) (need to multiply by 10^-9/yr)

column 5: dynamic strain tensor (exy) (need to multiply by 10^-9/yr)

Each cell includes 5 columns:

column 1: Longitude
column 2: Latitude
column 3: standard error for exx (need to multiply by 10^-9/yr)
column 4: standard error for eyy (need to multiply by 10^-9/yr)
column 5: standard error for exy (need to multiply by 10^-9/yr)

Supplementary Data 9: includes dynamic dilatational strain rates (ezz).

Each cell includes 3 columns:

column 1: Longitude
column 2: Latitude
column 3: dilatational strain rate (ezz) (need to multiply by 10^-9/yr)

Supplementary Data 10: includes the second invariant of dynamic strain rates and their associated standard errors.

Each cell includes 3 columns:

column 1: Longitude
column 2: Latitude
column 3: second invariant of dynamic strain rate (need to multiply by 10^-9/yr)

Each cell includes 3 columns:

column 1: Longitude
column 2: Latitude
column 3: standard error for the second invariant of dynamic strain rate (need to multiply by 10^-9/yr)

Supplementary Data 11: includes iterative dynamic deviatoric stress tensors and their associated standard errors.

Each cell includes 5 columns:

column 1: Longitude
column 2: Latitude
column 3: depth integrated horizontal deviatoric stress tensor (txx) in unit of Newton/meter
column 4: depth integrated horizontal deviatoric stress tensor (tyy) in unit of Newton/meter
column 5: depth integrated horizontal deviatoric stress tensor (txy) in unit of Newton/meter

Each cell includes 5 columns:

column 1: Longitude
column 2: Latitude
column 3: standard error for txx in unit of Newton/meter
column 4: standard error for tyy in unit of Newton/meter

column 5: standard error for txy in unit of Newton/meter

Supplementary Data 12: includes the second invariant of iterative dynamic deviatoric stresses and their associated standard errors.

Each cell includes 3 columns:

column 1: Longitude
column 2: Latitude
column 3: second invariant of depth integrated horizontal deviatoric stresses (MPa)

Each cell includes 3 columns:

column 1: Longitude
column 2: Latitude
column 3: standard error for the second invariant of depth integrated horizontal deviatoric stresses (MPa)

Supplementary Data 13: includes the effective viscosity of the lithosphere and their associated standard errors.

Each cell includes 3 columns:

column 1: Longitude
column 2: Latitude
column 3: log 10 values of the lithospheric effective viscosity (pa.s)

Each cell includes 3 columns:

column 1: Longitude
column 2: Latitude
column 3: standard error for log 10 values of the lithospheric effective viscosity (pa.s)

Supplementary Data 14: includes the boundary condition stress tensors.

Each cell includes 5 columns:

column 1: Longitude
column 2: Latitude
column 3: stress field boundary condition tensor (txx) in unit of Newton/meter
column 4: stress field boundary condition tensor (tyy) in unit of Newton/meter
column 5: stress field boundary condition tensor (txy) in unit of Newton/meter

---

Supplementary Data 15: includes the second invariant of boundary condition stresses.

Each cell includes 3 columns:

column 1: Longitude
column 2: Latitude
column 3: second invariant of stress field boundary condition associated with plate motion (MPa)

Supplementary Data 16: includes the strain tensors associated with plate motions.

Each cell includes 5 columns:

column 1: Longitude
column 2: Latitude
column 3: strain rate tensors associated with plate motions (exx) (need to multiply by 10^-9/yr)
column 4: strain rate tensors associated with plate motions (eyy) (need to multiply by 10^-9/yr)
column 5: strain rate tensors associated with plate motions (exy) (need to multiply by 10^-9/yr)

Supplementary Data 17: includes the second invariant of strain tensors associated with plate motions.

Each cell includes 3 columns:

column 1: Longitude
column 2: Latitude
column 3: second invariant of strain rate tensor associated with plate motions (need to multiply by 10^-9/yr)

Supplementary Data 18: includes the horizontal velocities associated with plate motions.

Each cell includes 7 columns:

column 1: Longitude
column 2: Latitude
column 3: x component of velocity vector associated with plate motions with unit of mm/yr
column 4: y component of velocity vector associated with plate motions with unit of mm/yr
column 5: standard error for x component of velocity vector with unit of mm/yr
column 6: standard error for y component of velocity vector with unit of mm/yr
column 7: correlation coefficient between x and y components of velocity vector

Supplementary Data 19: includes the ratio of the second invariant of deviatoric stresses associated with GPE differences and the second invariant of stress field boundary condition.

Each cell includes 3 columns:

column 1: Longitude
column 2: Latitude
column 3: ratio of the second invariant of deviatoric stresses associated with GPE differences and the second invariant of stress field boundary condition

Supplementary Data 20: includes the horizontal velocities associated with GPE gradients.

Each cell includes 7 columns:

column 1: Longitude

column 2: Latitude
column 3: x component of velocity vector associated with GPE gradients with unit of mm/yr
column 4: y component of velocity vector associated with GPE gradients with unit of mm/yr
column 5: standard error for x component of velocity vector with unit of mm/yr
column 6: standard error for y component of velocity vector with unit of mm/yr
column 7: correlation coefficient between x and y components of velocity vector

---

Supplementary Data 21: includes the strain tensors associated with GPE gradients.

Each cell includes 5 columns:

column 1: Longitude
column 2: Latitude
column 3: strain rate tensors associated with GPE gradients (exx) (need to multiply by 10^-9/yr)
column 4: strain rate tensors associated with GPE gradients (eyy) (need to multiply by 10^-9/yr)
column 5: strain rate tensors associated with GPE gradients (exy) (need to multiply by 10^-9/yr)

Supplementary Data 22: includes the second invariant of strain tensors associated with GPE gradients.

Each cell includes 3 columns:

column 1: Longitude
column 2: Latitude
column 3: second invariant of strain tensor associated with GPE gradients (need to multiply by 10^-9/yr)

Supplementary Data 23: includes the upper mantle temperature.

Each cell includes 3 columns:

column 1: Longitude
column 2: Latitude
column 3: averaged upper mantle temperature in unit of degree C.

Supplementary Data 24: includes the volumetric degree of melting for the lithosphere.

Each cell includes 3 columns:

column 1: Longitude
column 2: Latitude
column 3: volumetric degree of melting for the lithosphere

Supplementary Data 25: includes the effective density for upper mantle.

Each cell includes 3 columns:

column 1: Longitude

column 2: Latitude
column 3: effective density for upper mantle in unit of kg/m^3

Supplementary Data 26: includes the estimates for melt and fluid pressure factor.

Each cell includes 3 columns:

column 1: Longitude
column 2: Latitude
column 3: melt and fluid pressure factor estimates

Supplementary Data 27: includes the C_{OH} estimates for the lithosphere.

Each cell includes 3 columns:

column 1: Longitude
column 2: Latitude
column 3: log10 of C_{OH} estimates for the lithosphere

File Name: Supplementary Movie 1
Description: This movie shows the evolution of the model velocity field estimates (red vectors) relative to the North American frame for Farallon, Pacific, and North American plates superimposed on paleo-elevation model of Bahadori et al. (2018).

File Name: Supplementary Movie 2
Description: This movie shows the variation of the averaged upper mantle temperature from Moho boundary to 100 km depth. The present-day temperature model is achieved from seismic shear wave velocities, lithospheric density, and pressure. The temperature variation through time is calculated using a steady-state conductive heat distribution model obtained from distribution of active magmatism in southwestern North America from Late Eocene to present-day. Red and grey dots represent reconstructed coordinates of present-day localities of paleo-magmatism in southwestern North America. Red dots represent active magmatism and grey dots represent previously active magmatism.

File Name: Supplementary Movie 3
Description: This movie shows the upper mantle density changes inferred from models of lithospheric pressure, upper mantle temperature and volumetric degree of melting in southwestern North America.

File Name: Supplementary Movie 4
Description: This movie shows the evolution of vertically averaged horizontal deviatoric stress field superimposed on associated gravitational potential energy variations within 3 the lithosphere. Red arrows represent tensional principal axes of deviatoric stress, and black arrows represent compressional principal axes of deviatoric stress in southwestern North America.

File Name: Supplementary Movie 5
Description: This movie shows the evolution of vertically averaged horizontal deviatoric stress field associated with gravitational potential energy variations within the lithosphere superimposed on paleo-elevation model of Bahadori et al. (2018). Red arrows represent tensional principal axes of deviatoric stress, and black arrows represent compressional principal axes of deviatoric stress in southwestern North America.

File Name: Supplementary Movie 6
Description: This movie shows the variation of the second invariant of vertically averaged horizontal deviatoric stress field associated with GPE differences (TGPE) in southwestern North America.

File Name: Supplementary Movie 7
Description: This movie shows the variation of the stress field boundary conditions in southwestern North America. Red arrows represent tensional principal axes of deviatoric stress, and black arrows represent compressional principal axes of deviatoric stress.

File Name: Supplementary Movie 8
Description: This movie shows the variation of the ratio of the second invariant of vertically averaged deviatoric stresses from GPE differences and the second invariant of stress field boundary conditions (TGPE/TBCS) in southwestern North America. Red vectors are predicted velocity field from iterative dynamic model with 95% confidence error ellipse.

File Name: Supplementary Movie 9
Description: This movie shows the evolution of the effective viscosity of the lithosphere in southwestern North America obtained from deviatoric stress field of iterative dynamic model and kinematic strain rate. Arrows represent deviatoric stress fields using GPE values from topography and crustal thickness models of Bahadori et al. (2018). Red arrows represent tensional principal axes of deviatoric stress, and black arrows represent compressional principal axes of deviatoric stress.

File Name: Supplementary Movie 10
Description: This movie shows the evolution of the vertically averaged deviatoric stress field obtained from iterative dynamic model superimposed on paleo-elevation model of Bahadori et al. (2018) in southwestern North America. Red arrows represent tensional principal axes of deviatoric stress, and black arrows represent compressional principal axes of deviatoric stress.

File Name: Supplementary Movie 11
Description: This movie shows the evolution of the second invariant of vertically averaged deviatoric stress fields (Tdyn) obtained from iterative dynamic model in southwestern North America.

File Name: Supplementary Movie 12
Description: This movie shows the correlation between stretch directions of Metamorphic Core Complexes (red dots), Miocene fault (green dots), Miocene dyke (blue) trends and the orientation of the tensional deviatoric stress field obtained from iterative dynamic model superimposed on paleo-elevation model of Bahadori et al. (2018). Red arrows represent tensional principal axes of deviatoric stress, and black arrows represent compressional principal axes of deviatoric stress. Gray dots represent previously active Metamorphic Core Complexes, faults, and dykes.

File Name: Supplementary Movie 13
Description: This movie shows the variation of volumetric degree of melting (M0) of the lithosphere in southwestern North America.

File Name: Supplementary Movie 14
Description: This movie shows the variation of melt and fluid pressure factor (λ) for a partially molten wet upper mantle (wet olivine).

File Name: Supplementary Movie 15
Description: This movie shows the variation of molten (red areas) and non-molten (blue areas) phases of the lithosphere in southwestern North America. Grey dots represent the reconstructed potion of present-day coordinates of paleo-magmatism in Southwestern North America.

File Name: Supplementary Movie 16
Description: This movie shows the variation of COH for distributed water in nominally anhydrous minerals like olivine.

File Name: Supplementary Movie 17
Description: This movie shows the evolution of the dilatational strain rates obtained from iterative dynamic model in southwestern North America. Red arrows represent tensional principal axes of strain, and black arrows represent compressional principal axes of strain.

File Name: Supplementary Movie 18
Description: This movie shows the evolution of the dilatational strain rates obtained from iterative dynamic model in southwestern North America which includes the combination of GPE gradients and the relative plate motion. Red vectors are predicted velocity fields from iterative dynamic model relative to the North American frame.

File Name: Supplementary Movie 19
Description: This movie shows the variation of the second invariant of strain rates from iterative dynamic model in southwestern North America. Red arrows represent tensional principal axes of strain, and black arrows represent compressional principal axes of strain.

File Name: Supplementary Movie 20
Description: This movie shows the evolution of predicted velocity fields (green vectors) from iterative dynamic model relative to the North American frame which includes the combination of GPE gradients and the relative plate motion superimposed on contoured second invariant strain rates (E).

File Name: Supplementary Movie 21
Description: This movie shows the evolution of the velocity field estimates relative to the North American frame superimposed on paleo-elevation model of Bahadori et al. (2018). Green vectors are model velocities from the kinematic model. Red vectors are predicted velocity field from iterative dynamic model.

File Name: Supplementary Movie 22
Description: This movie shows the evolution of the model velocity field estimates (green vectors) associated with GPE gradients only relative to the North American frame superimposed on their associated second invariant of strain rates (E).

File Name: Supplementary Movie 23
Description: This movie shows the variation of the principal axes of strain tensors and contoured second invariant of strain rates (E) associated with GPE gradients only in southwestern North America. Red arrows represent tensional and black arrows represent compressional principal axes of strain rates.

File Name: Supplementary Movie 24
Description: This movie shows the variation of the principal axes of boundary condition strain tensors and their associated contoured second invariant of strain rates (E) in southwestern North America. Red arrows represent tensional and black arrows represent compressional principal axes of strain rates.

File Name: Supplementary Movie 25
Description: This movie shows the evolution of the model boundary condition velocity field estimates (green vectors) relative to the North American frame for Farallon, Pacific, and North American plates superimposed on contoured second invariant of boundary condition strain rates (E).

File Name: Supplementary Movie 26
Description: This movie shows the evolution of boundary condition velocity field estimates (green vectors) relative to the North American frame for Farallon, Pacific, and North American plates, and the evolution of velocity field estimates associated with GPE gradients only relative to the North American frame (red vectors).

File Name: Supplementary Movie 27
Description: This movie shows the variation of standard errors for GPE in southwestern North America through time.

File Name: Supplementary Movie 28
Description: This movie shows the variation of standard errors for the second invariant of vertically averaged deviatoric stresses associated with GPE differences in southwestern North America through time.

File Name: Supplementary Movie 29
Description: This movie shows the variation of standard errors for lithospheric effective viscosity obtained by running iterative dynamic model 100 times using the 100 realized GPE fields in southwestern North America.

File Name: Supplementary Movie 30
Description: This movie shows the variation of standard errors for the second invariant of vertically averaged deviatoric stress fields form iterative dynamic model in southwestern North America.

File Name: Supplementary Movie 31
Description: This movie shows the variation of standard errors for the second invariant of strain rates from iterative dynamic model in southwestern North America.
