# Supplementary material for: Geodynamic evolution of southwestern North America since the Late Eocene
Source: Nat Commun. 2019 Nov 18;10:5213. doi: 10.1038/s41467-019-12950-8 (PMC6861285; doi:10.1038/s41467-019-12950-8)
Supplement: Supplementary file 2 — Supplementary Information [file 41467_2019_12950_MOESM2_ESM.pdf]

Supplementary Information for

Geodynamic evolution of southwestern North America since the Late Eocene

Alireza Bahadori and William E. Holt

Department of Geosciences, Stony Brook University, Stony Brook, NY, USA

Supplementary Note 1

Discussion for Supplementary Figure 1: With a defined distribution of horizontal gradients of depth integrated vertical deviatoric stresses (GPE per unit area) based on paleo-topography and paleo-crustal thickness model of Bahadori et al.[1] (Supplementary Fig. 1a), velocity boundary conditions relative to the North American plate (Supplementary Fig. 1b), and lateral variations in depth integrated effective viscosity of the lithosphere (Supplementary Fig. 1c), we minimize (11) in Methods (or (5) below) to obtain force-balance solutions with output of depth-integrated horizontal deviatoric stresses, strain rates and surface velocities from surface to 100 km below sea level in southwestern North America (Supplementary Fig. 1d-f). For the benchmarking, we take the GPE distribution, the velocity boundary conditions, and the scalar values of strain rates (E) as input for the iterative dynamic model solution. Our goal is to determine if we can recover the true deviatoric stress field, the true output strain tensor field, and the true effective viscosity field by first guessing a uniform viscosity value everywhere and then iterating until we achieve convergence. We call this a blind test because we start the iteration procedure without knowing the viscosity field by choosing a constant value.

We solve the depth-integrated 3-D spherical form of the force-balance equations[2–4] for depth integrated horizontal deviatoric stresses. The cartesian form of these equations is:

$$\frac{\partial \sigma_{ij}}{\partial x_j} + \rho g_i = 0, \qquad (1)$$

where σ_{ij} is the stress tensor, g is the acceleration of gravity, and ρ is density, and with summation notation, i and $j = x, y$ and z. Vertical integration of the force-balance equations, using summation notation, results in the following:

$$-\frac{\partial \bar{\sigma}_{zz}}{\partial x_\alpha} = \frac{\partial}{\partial x_\beta}(\bar{\tau}_{\alpha\beta} + \delta_{\alpha\beta}\bar{\tau}_{\gamma\gamma}), \qquad (2)$$

where $\bar{\sigma}_{zz}$ is the depth-integrated vertical stress or GPE per unit area, $\bar{\tau}_{\alpha\beta}$ is the depth-integrated horizontal deviatoric stress tensor, $\bar{\tau}_{\gamma\gamma} = (\bar{\tau}_{xx} + \bar{\tau}_{yy}) = -\bar{\tau}_{zz}$, and $\delta_{\alpha\beta}$ is the Kronecker delta. Equation (2) assumes that horizontal gradients of the vertical integrals of shear stress (basal tractions), $\bar{\tau}_{xz}$ and $\bar{\tau}_{yz}$, are small in comparison with the product of acceleration of gravity and density,

$$\frac{\partial}{\partial x}\int_{-h}^{z} \tau_{xz}(z)\, dz + \frac{\partial}{\partial y}\int_{-h}^{z} \tau_{yz}(z)\, dz << \int_{-h}^{z} \rho(z) g dz. \qquad (3)$$

We solve the force-balance equations

$$-\frac{\partial \bar{\sigma}_{zz}}{\partial x_\alpha} = \frac{\partial}{\partial x_\beta}(\bar{\tau}_{\alpha\beta} + \delta_{\alpha\beta}\bar{\tau}_{\gamma\gamma}), \qquad (4)$$

through the optimization of the functional[5]:

$$\Theta = \iint_s [D + \dot{\varepsilon}_{\gamma\gamma}\bar{\sigma}_{zz}]dxdy - \int_{\partial S} v_\alpha \bar{\sigma}_{zz} n_\alpha dl, \qquad (5)$$

where D is the dissipation potential, $\bar{\sigma}_{zz}$ is GPE, and v_α is the velocity boundary conditions of the relative motions of Pacific- and Farallon-North America plates applied around the grid area boundary (∂S) with normal vector n_α. The dissipation potential (D) is a function of strain rates, rheological parameter (B), and the power law exponent (n)[4]:

$$D = \frac{n}{n+1} B(\dot{\varepsilon}_{\alpha\beta}\dot{\varepsilon}_{\alpha\beta} + \dot{\varepsilon}_{\gamma\gamma}\dot{\varepsilon}_{\gamma\gamma})^{\frac{n+1}{2n}}, \quad (6)$$

where $\dot{\varepsilon}_{\gamma\gamma} = (\dot{\varepsilon}_{xx} + \dot{\varepsilon}_{yy}) = -\dot{\varepsilon}_{zz}$. The effective viscosity $\bar{\eta} = BE^{1/n-1}$, where B is the rheological parameter that is sensitive to temperature[6,7], n is the power-law exponent, and E is the second invariant of strain rate. E and T are calculated as:

$$E = \sqrt{\dot{\varepsilon}_{xx}^2 + \dot{\varepsilon}_{yy}^2 + \dot{\varepsilon}_{zz}^2 + \dot{\varepsilon}_{xy}^2 + \dot{\varepsilon}_{yx}^2} = \sqrt{2\dot{\varepsilon}_{xx}^2 + 2\dot{\varepsilon}_{yy}^2 + 2\dot{\varepsilon}_{xx}\dot{\varepsilon}_{yy} + 2\dot{\varepsilon}_{xy}^2},$$

$$T = \sqrt{\tau_{xx}^2 + \tau_{yy}^2 + \tau_{zz}^2 + \tau_{xy}^2 + \tau_{yx}^2} = \sqrt{2\tau_{xx}^2 + 2\tau_{yy}^2 + 2\tau_{xx}\tau_{yy} + 2\tau_{xy}^2}.$$

Supplementary Note 2

Discussion for Supplementary Figure 2: Through benchmarking tests, we have found that the true dynamic solution for effective viscosity and lithospheric deviatoric stresses can be recovered, blind, using an iterative method. We show in supplementary Figures 1 and 2 that if far-field velocity boundary conditions, GPE values (paleo-topography and crustal structure), and scalar values of kinematic strain rates (E) are known through time, then it is possible to ***uniquely*** recover the absolute magnitudes of vertically averaged deviatoric stresses and effective viscosities within the lithosphere using this iterative procedure involving solutions to the force-balance equations, given topography, crustal thicknesses, densities, and their errors. This complete solution involves the combination of stresses associated with GPE gradients and stresses that accommodate the relative plate motion. We arrive at the optimal solution, involving minimization of (5).

iteratively (Supplementary Fig. 2). We first solve the force-balance equations associated with GPE distributions alone

$$-\frac{\partial \bar{\sigma}_{zz}}{\partial x_\alpha} = \frac{\partial}{\partial x_\beta}(\bar{\tau}_{\alpha\beta} + \delta_{\alpha\beta}\bar{\tau}_{\gamma\gamma}),$$

assuming constant viscosity and zero velocity boundary conditions in Θ along a rigid boundary. The deviatoric stress solution, obtained through minimization of Θ, depends only on the gradient in GPE and is invariant to the magnitude of the constant value for initial effective viscosity (Supplementary Fig. 2a). This solution provides initial estimates of the distribution of deviatoric stresses that balance gradients of GPE. We then estimate an initial distribution of B, using $n = 1$ and $\bar{\eta} = B = \frac{T}{E}$, where E is the second invariant of strain rates (scalar values) from the kinematic solution (Supplementary Fig. 2b, c). Using this laterally varying first estimate of the distribution of B, we seek the complete solution by solving the force balance through minimization of Θ with respect to model strain rates $\dot{\varepsilon}_{\alpha\beta}$, using the distribution of GPE and velocity boundary conditions that are appropriate for the given time interval. Model values of strain rate, multiplied by the previous iteration's viscosity, provide updated deviatoric stresses, $\bar{\tau}_{\alpha\beta}$ (Supplementary Fig. 2d). Using the updated deviatoric stresses we calculate a new effective viscosity field and a new force balance solution (Supplementary Fig. 2d, g, j, m). The procedure is repeated until there are no further changes in T and model values of strain rate $\dot{\varepsilon}_{\alpha\beta}$ (Supplementary Fig. 2m, n, o). Supplementary Table 1, and examination of Supplementary Figure 2, indicates that by 12 iterations the model solution has nearly converged and differences between the "blind" test and the true solution are small (2% error in viscosity, correlation between tensor fields of 0.997, RMS misfit of velocities of 0.4 mm/yr).

Supplementary Note 3

Discussion for Supplementary Figure 3a-f: Using the final viscosity structure from complete solution and zero GPE gradients,

$$0 = -\frac{\partial \bar{\sigma}_{zz}}{\partial x_\alpha} = \frac{\partial}{\partial x_\beta}(\bar{\tau}_{\alpha\beta} + \delta_{\alpha\beta}\bar{\tau}_{\gamma\gamma}),$$

while applying the correct velocity boundary conditions of the relative motions of Pacific- and Farallon-North America plates around the grid area boundary (∂S) (Supplementary Fig. 3.1a-c), we recover boundary condition stress, strain rate and velocity solutions (Supplementary Fig. 3.1d-f) by solving the force-balance equations

$$-\frac{\partial \bar{\sigma}_{zz}}{\partial x_\alpha} = \frac{\partial}{\partial x_\beta}(\bar{\tau}_{\alpha\beta} + \delta_{\alpha\beta}\bar{\tau}_{\gamma\gamma}),$$

through the optimization of (5).

Supplementary Note 4

Discussion for Supplementary Figure 3g-l: Given the constraints of GPE magnitudes (Supplementary Fig. 3.2a) and final estimates of laterally varying effective viscosity for the lithosphere (Supplementary Fig. 3.2b), we redetermine the deviatoric stresses associated with GPE alone $(\bar{\tau}'_{\alpha\beta})_{\mathrm{GPE}}$ (Supplementary Fig. 3.2c) by solving

$$-\frac{\partial \bar{\sigma}_{zz}}{\partial x_\alpha} = \frac{\partial}{\partial x_\beta}(\bar{\tau}'_{\alpha\beta} + \delta_{\alpha\beta}\bar{\tau}_{\gamma\gamma}).$$

We then subtract the components of these new deviatoric stresses associated with GPE gradients (Supplementary Fig. 3.2e) from the complete solution (Supplementary Fig. 3.2d).

$$(\bar{\tau}_{\alpha\beta})_{\mathrm{BCS}} = (\bar{\tau}_{\alpha\beta})_{\mathrm{dyn}} - (\bar{\tau}'_{\alpha\beta})_{\mathrm{GPE}}$$

The residual constitutes the contribution in stresses associated with the accommodation of plate motion alone (Supplementary Fig. 3.2f). Comparison of the boundary conditions using the two methods shows that they are nearly identical.

Supplementary Note 5

Discussion for Supplementary Figure 3m, n: Our model boundary conditions stresses (Supplementary Fig. 3a) for present-day appear similar in magnitude and direction to deviatoric stresses that arise from global calculations of mantle flow coupling (Supplementary Fig. 3b) with the lithosphere[46]. This agreement suggests that it is the longer wavelength component of the horizontal traction field (which integrates over thousands of kilometers to become important) associated with mantle convection[46] that is reflected in boundary condition stress field solutions for southwestern North America. That is, the distances between plate margin edge and interior deformation zone are small enough such that tractions directly below deforming regions can be ignored without introducing large errors. Thus, tractions associated with smaller-scale convection, such as slab rollback before, during, and after the collapse of Mogollon Highlands and Nevadaplano, are expected to play a much lesser role in their influence on the stress field than do the GPE gradients, which are modeled in this study. The history of slab rollback, on the other hand, is important to

explain the deformation style by introducing fluids and melts, and through the influence on mantle temperature changes, all of which directly impact rheology.

Supplementary Note 6

Discussion for Supplementary Figure 5: Behr and Platt[8] determined a naturally constrained stress profile through the middle crust for the footwall of the Whipple Mountains MCC. Their results constrain a minimum depth-averaged strength profile of ≥15 MPa for the entire lithosphere (100 km thickness) during Miocene extension, in agreement with our model stress values of >15 MPa. We have tested the possibility of a much lower topography (lower GPE) in our model geodynamic estimates during the extensional collapse. In the absence of high paleotopography at the time of extension initiation, the model fails to converge, the distribution of the strain rates fails to match expected stretch distributions, and final deviatoric stress magnitudes are too low (5 MPa or less) (Supplementary Fig. 5). This suggests that a Mogollon Highlands, supported by a thick crustal root, are required to explain stress magnitudes[8] and strain along the belt of MCCs from southeast to northwest Arizona. This further suggests that if far-field velocity boundary conditions, GPE values (paleo-topography and crustal structure), and scalar values of kinematic strain rates (*E*) are known through time, then the absolute magnitudes of vertically averaged deviatoric stresses and effective viscosities within the lithosphere can be uniquely recovered through solutions to the force-balance equations (Supplementary Figs. 5). This test also suggests that comparisons of model output (strain tensor orientations, relative velocities) with the kinematic model is a viable means of testing the validity of paleo-topographic reconstructions. That is, these exercises demonstrate that having the wrong internal body force distribution leads to problems in predicting correct stretch directions and relative motions within the plate boundary zone, even when relative plate velocity boundary conditions and scalar values of strain rate, *E*, are known.

Supplementary Note 7

Discussion for Supplementary Table 1: The number of areas in the second column denotes total number of cells on our southwestern North America grid that the second invariant of strain rate (*E*) is more than zero.

The average misfit to the functional[32,33] (M_f) is calculated as:

$$M_f = \frac{\sum_{areas}(T - \frac{e \cdot \tau}{E})\, \Delta S^*}{\sum_{areas} \Delta S},$$

where e is the strain rate tensor, E is the second invariant of strain rate, τ is the deviatoric stress tensor, T is the second invariant of deviatoric stress, and $e \cdot \tau$ is the inner product of the tensor components.

The average correlation between the unit tensors[2–4] (C_{ut}) is calculated as:

$$C_{ut} = \frac{\sum_{areas}(\frac{e \cdot \tau}{ET})\, \Delta S}{\sum_{areas} \Delta S}.$$

The average misfit between the unit tensors[2–4] (M_{ut}) is calculated as:

$$M_{ut} = \frac{\sum_{areas}\ 0.5*(1 - \frac{e \cdot \tau}{ET})\, \Delta S}{\sum_{areas} \Delta S}.$$

V_{rms} misfit[11,12] in units of mm/yr is calculated as:

$V_{rms} = \sqrt{\frac{1}{2N}\sum\{(\Delta v_x)^2 + (\Delta v_y)^2\}}.$

The average of the second invariant of strain rate (E_{avg}) in units of 10^{-9} . yr^{-1} is calculated as:

$E_{avg} = \frac{\sqrt{\sum_{areas}(E)^2 \Delta S}}{\sum_{areas} \Delta S}.$

[*] The regional area normalized by the square of the radius of the Earth is denoted as: ΔS.

Supplementary Note 8

Discussion for Supplementary Table 2: We first consider fault and dyke trends and metamorphic core complex stretch directions as elongated objects. We then use the method of Holt and Haines[13] to calculate the rotation magnitudes and orientation updates for those line elements of a crustal block during each half a million-year time interval (k=1-73, where k=1 is present-day and k=73 is 36 Ma). That method argues that inside a zone of distributed deformation containing crustal blocks, with length dimensions much greater than their widths, the rotation rate is similar to the rotation of a line element connecting two points within a straining region. We treat metamorphic core complex stretch directions, and Miocene fault and dyke orientations as line elements embedded within a time-dependent velocity gradient tensor field. Our goal is to take the present-day orientations and account for the rotational part of the velocity gradient tensor field to determine their orientations at the time of their formation. The instantaneous rotation rate $(\frac{d\theta}{dt})$ of an elongated element (e.g. fault, dyke, etc.) located within a deforming region for each time increment (k = 1-73) is strongly dependent on the orientation of the line element, the geodetic rotation rate $(\dot{\omega})$, and the strain rate tensor field $(\dot{\epsilon}_{xx}, \dot{\epsilon}_{yy}, \dot{\epsilon}_{xy})$[13],

$$(\frac{d\theta}{dt})_{k+1} = (\dot{\omega})_{k+1} + (\dot{\epsilon}_{xy})_{k+1} \cos 2(\theta)_k + \frac{1}{2}[(\dot{\epsilon}_{yy})_{k+1} - (\dot{\epsilon}_{xx})_{k+1}] \sin(\theta)_k,$$

where $\dot{\omega}$ is the geodetic rotation rates, θ is the azimuth of fault, dyke or stretch direction of metamorphic core complexes, and $\dot{\epsilon}_{xx}$, $\dot{\epsilon}_{yy}$, and $\dot{\epsilon}_{xy}$ are the strain rate tensors achieved from kinematic solution.

To calculate the orientation at the time of formation we sum the azimuthal changes using time increments of 0.5 Myr, $(\Delta\theta)_{k+1}$, and because rotation rate units are in radians per year, we multiply $(\frac{d\theta}{dt})_{k+1}$ by 0.5×10^6 yr, $(\frac{d\theta}{dt})_{k+1} \times 0.5 \times 10^6$ yrs $= (\Delta\theta)_{k+1}$.

The rotational part of the field within the Great Basin and southern Basin and Range is generally considerably less than, for example, the Transverse Ranges, and we find total finite rotations to be generally less than 10 degrees.

Adding the values of $(\Delta\theta)_{k+1}$ to $(\theta)_k$ we calculate the new azimuths for fault and dyke trends, and metamorphic core complex stretch directions at times k+1:

$$(\theta)_k + [(\Delta\theta)_{k+1} \times \frac{180}{\pi}] = (\theta)_{k+1}$$

We calculate the misfits between principal axes of tensional deviatoric stresses from our iterative dynamic models and the time-dependent estimates for azimuth of the Miocene faults and dykes as well as the stretch directions of the metamorphic core complexes in southwestern North America (Supplementary Table S1). It has been argued by Haines and Holt[13] and McKenzie and Jackson[14] that in the case where crustal blocks are

equidimensional, then they will rotate at the geodetic rate ($\dot{\omega}$). That is, the geodetic rotation rate ($\dot{\omega}$) will be equal to the average of ($\frac{d\theta}{dt}$) for all azimuths, θ, of the line elements[13]. We have tried both methods and obtained similar results, but misfits were slightly improved assuming the line element approach.

Supplementary Note 9

Although our model geodynamic results are generally consistent with the observational constraints (Supplementary Tables 1, 2), our thin viscous sheet modeling approach is only capable of capturing the long wavelength (~150-250 km) features for viscous processes that relax topography and smooth lithosphere-scale continuous responses to tectonic forcing[13]. Our modeling approach, on the other hand, does not capture short characteristic wavelength (~10-50 km) features of active strain associated with faulting in the elastic layer. As such, misfits between our model geodynamic results and geological observations (Supplementary Tables 1, 2) can be potentially improved by future work that includes a 3-D coupled thermal-mechanical approach to produce a refined paleo-crustal structure and its associated paleo-elevation for Nevadaplano and Mogollon Highlands. Refined estimates of paleo-elevations may also lead to improved thin viscous sheet models. However, a 3-D approach is vital for modeling deformation over length scales much less than we model here, on the order of lithospheric thickness. Such a 3-D approach will be important for modeling the complete field at all characteristic wavelengths[13], including the modeling of lower crustal flow in the vicinity of individual core complexes.

Supplementary Note 10

Discussion for Supplementary Figure 6: To calculate the standard error for depth integrated vertical stress within the lithosphere, σ_{zz}, also known as gravitational potential energy per unit area, GPE, ($\bar{\sigma}_{zz}$ = -g$\int_{-h}^{L}[\int_{-h}^{z}\rho(z')dz']dz$), we use the standard errors of Bahadori et al.[1] for paleo-crustal thickness (H), and paleo-elevation (h) variations from 36 Ma to present-day. We consider a 10% standard error for the time-dependent effective upper mantle density model ($\rho_{m'}$) from Bahadori et al.[1] by comparing the upper mantle density model of Bahadori et al.[1] with the density model we have produced from an upper mantle temperature model that relies on seismic shear wave velocities[14] and a steady-state conductive heat distribution model, associated with positions and migrations of volcanism[15] from 36 Ma to present.

Since crustal density ($\rho_c = 2700\ \mathrm{kg/m^3}$)[1] and acceleration of gravity (g = 9.8 m/s^2) are both constant, then the variance of crustal pressure at Moho depth for each 0.5 myr time interval (k = 1-73) inside each cell, latitude: θ, and longitude: $\emptyset$, of our grid area, $P(z_1)_{k(\theta,\emptyset)}$ = g . $\rho_c . H_{k(\theta,\emptyset)}$, is:
var[$P(z_1)_{k(\theta,\emptyset)}$] = $(\rho_c.\mathrm{g})^2$. var$(H)_{k(\theta,\emptyset)}$.

Having a variable upper mantle density ($\rho_{m'}$)[1], then the variance of lithospheric pressure at 100 km depth, $P(z_3) = P(z_1) + P(z_2)$, is the sum of variances for lithospheric mantle and crustal pressures as:
var[$P(z_3)_{k(\theta,\emptyset)}$] = var[$P(z_1)_{k(\theta,\emptyset)}$+ $P(z_2)_{k(\theta,\emptyset)}$].

The variance for lithospheric mantle pressure, $P(z_2)_{k(\theta,\emptyset)}$, is:
Var[$P(z_2)_{k(\theta,\emptyset)}$] = var[$\rho_{m'_{k(\theta,\emptyset)}}$. g . (100000+$\mathrm{h}_{k(\theta,\emptyset)}$-$\mathrm{H}_{k(\theta,\emptyset)}$)],
where $\rho_{m'_{k(\theta,\emptyset)}}$. g . 100000 = A, and $\rho_{m'_{k(\theta,\emptyset)}}$. g . ($\mathrm{h}_{k(\theta,\emptyset)}$-$\mathrm{H}_{k(\theta,\emptyset)}$) = B. Hence, the variances of A and B are:

var(A) = $(\mathrm{g}.100000)^2$ var($\rho_{m'_{k(\theta,\emptyset)}}$),

var(B) = (g)^2. var($\rho_{m'_{k(\theta,\emptyset)}}$. ($h_{k(\theta,\emptyset)}$-$H_{k(\theta,\emptyset)}$)) = (g)^2. [($\bar{\rho}_{m'_{k(\theta,\emptyset)}}$)^2 . var($h_{k(\theta,\emptyset)}$-$H_{k(\theta,\emptyset)}$)+(($\overline{h_{k(\theta,\emptyset)} - H_{k(\theta,\emptyset)}}$)^2. var($\rho_{m'_{k(\theta,\emptyset)}}$))+2(($\bar{\rho}_{m'_{k(\theta,\emptyset)}}$) . ($\overline{h_{k(\theta,\emptyset)} - H_{k(\theta,\emptyset)}}$) . cov(($\rho_{m'_{k(\theta,\emptyset)}}$),($h_{k(\theta,\emptyset)}$-$H_{k(\theta,\emptyset)}$))+var($\rho_{m'_{k(\theta,\emptyset)}}$) . var($h_{k(\theta,\emptyset)}$-$H_{k(\theta,\emptyset)}$)+cov^2(($\rho_{m'_{k(\theta,\emptyset)}}$),($h_{k(\theta,\emptyset)}$-$H_{k(\theta,\emptyset)}$))].

Variance of $h_{k(\theta,\emptyset)}$-$H_{k(\theta,\emptyset)}$ and covariance of $\rho_{m'_{k(\theta,\emptyset)}}$ and $h_{k(\theta,\emptyset)}$-$H_{k(\theta,\emptyset)}$ are calculated as:
var($h_{k(\theta,\emptyset)}$-$H_{k(\theta,\emptyset)}$) = var($h_{k(\theta,\emptyset)}$) + var($H_{k(\theta,\emptyset)}$) – 2cov($h_{k(\theta,\emptyset)}$, $H_{k(\theta,\emptyset)}$),
cov(($\rho_{m'_{k(\theta,\emptyset)}}$), ($h_{k(\theta,\emptyset)}$-$H_{k(\theta,\emptyset)}$)) = $\frac{1}{N-1}\sum_{i=1}^{N}(\rho_{m'}{}_{i_{k(\theta,\emptyset)}} - \bar{\rho}_{m'}{}_{k(\theta,\emptyset)})$. [($h_{i_{k(\theta,\emptyset)}}$-$H_{i_{k(\theta,\emptyset)}}$) – ($\overline{h_{k(\theta,\emptyset)} - H_{k(\theta,\emptyset)}}$)],
where cov($h_{k(\theta,\emptyset)}$, $H_{k(\theta,\emptyset)}$) = $\frac{1}{N-1}\sum_{i=1}^{N}(h_{i_{k(\theta,\emptyset)}} - \bar{h}_{k(\theta,\emptyset)}) . (H_{i_{k(\theta,\emptyset)}} - \bar{H}_{k(\theta,\emptyset)})$, and N = 25.

Thus, variance of pressure for lithospheric mantle is:
Var[$P(z_2)_{k(\theta,\emptyset)}$] = var(A) + var(B) + 2cov(A,B).

Finally, the variance of depth integrated vertical deviatoric stresses or gravitational potential energy per unit area is:
$\mathrm{Var(GPE)}_{k(\theta,\emptyset)}$ = var {$\frac{1}{2}$ [$P(z_1)_{k(\theta,\emptyset)}$.$H_{k(\theta,\emptyset)}$] + $\frac{1}{2}$ [$P(z_1)_{k(\theta,\emptyset)}$ + $P(z_2)_{k(\theta,\emptyset)}$] . (100000+ $h_{k(\theta,\emptyset)}$- $H_{k(\theta,\emptyset)}$)}.

Omitting details, we consider P$(z_1)_{k(\theta,\emptyset)}$. (100000+$h_{k(\theta,\emptyset)}$) = C, and P$(z_2)_{k(\theta,\emptyset)}$. (100000+ $h_{k(\theta,\emptyset)}$- $H_{k(\theta,\emptyset)}$) = D. Hence, the variance of GPE can be written as:
$\mathrm{Var(GPE)}_{k(\theta,\emptyset)}$= ($\frac{1}{2}$)^2 . var(C+D).

The variances of C and D are:
var(C) = $\bar{P}(z_1)^2_{k(\theta,\emptyset)}$. var($h_{k(\theta,\emptyset)}$)+(($\overline{100000 + h_{k(\theta,\emptyset)}}$)^2 . var(P$(z_1)_{k(\theta,\emptyset)}$)+2($\bar{P}(z_1)_{k(\theta,\emptyset)}$) . ($\overline{100000 + h_{k(\theta,\emptyset)}}$). cov(P$(z_1)_{k(\theta,\emptyset)}$,(100000+$h_{k(\theta,\emptyset)}$)]+var(P$(z_1)_{k(\theta,\emptyset)}$) . var($h_{k(\theta,\emptyset)}$)+cov^2(P$(z_1)_{k(\theta,\emptyset)}$,$(100000 + h_{k(\theta,\emptyset)}$).
var(D) = var [P$(z_2)_{k(\theta,\emptyset)}$] . (100000+ $h_{k(\theta,\emptyset)}$- $H_{k(\theta,\emptyset)}$).

By having 100000+ $h_{k(\theta,\emptyset)}$- $H_{k(\theta,\emptyset)}$ = Z_2, then the variance of D is:
var(D) = var[P$(z_2)_{k(\theta,\emptyset)}$. $(Z_2)_{k(\theta,\emptyset)}$] = $\bar{P}(z_2)^2_{k(\theta,\emptyset)}$. var($(Z_2)_{k(\theta,\emptyset)}$)+(($\bar{Z}_2)^2_{k(\theta,\emptyset)}$. var(P$(z_2)_{k(\theta,\emptyset)}$)+2($\bar{P}(z_2)_{k(\theta,\emptyset)}$) . $(\bar{Z}_2)_{k(\theta,\emptyset)}$. cov[P$(z_2)_{k(\theta,\emptyset)}$, $(Z_2)_{k(\theta,\emptyset)}$]+var(P$(z_2)_{k(\theta,\emptyset)}$).var($(Z_2)_{k(\theta,\emptyset)}$)+cov^2(P$(z_2)_{k(\theta,\emptyset)}$, $(Z_2)_{k(\theta,\emptyset)}$).

Finally, variance and standard error of GPE are:
$\mathrm{var(GPE)}_{k(\theta,\emptyset)}$ = ($\frac{1}{2}$)^2 . [var(C)+var(D)+2cov(C, D)],
$\mathrm{se(GPE)}_{k(\theta,\emptyset)}$= $\sqrt{\mathrm{var(GPE)}_{k(\theta,\emptyset)}}$.

Supplementary Note 11

Discussion for Supplementary Figure 7: To calculate the standard error for the second invariant of deviatoric stresses associated with GPE differences for a given time slice (k), we used a bootstrapping method to generate 100 realizations of deviatoric stress fields associated with 100 GPE fields. Each realized GPE ($R\mathrm{GPE}_{i_{k(\theta,\emptyset)}}$) value for the ith individual area (i = 1-2376) within a single realized field of GPE was obtained using the expected value of GPE ($E\mathrm{GPE}_{i_{k(\theta,\emptyset)}}$) plus the associated standard error of GPE

($se\mathrm{GPE}_{i_{k(\theta,\emptyset)}}$) for that area, multiplied by a random number ($RN_{i_{k(\theta,\emptyset)}}$) that yields a normalized Gaussian distribution with a mean value of zero, where $R\mathrm{GPE}_{i_{k(\theta,\emptyset)}} = E\mathrm{GPE}_{i_{k(\theta,\emptyset)}} + (se\mathrm{GPE}_{i_{k(\theta,\emptyset)}} \times RN_{i_{k(\theta,\emptyset)}})$.

We then apply a smoothing of realized GPE estimates for the whole southwestern North America for each half a million year. This smoothing is performed in order to approximate the correlation of GPE errors between adjacent cells. The smoothing is applied such that the standard error for each individual area only influences the eight areas surrounding it; in the case of an edge location it is the five areas surrounding it, and in the case of a corner location, it is the three areas surrounding it.

Once 100 estimates of realized GPE values are achieved, we solve the force balance equations 100 times to calculate the depth-integrated horizontal deviatoric stresses associated with GPE differences. This gives us 100 values for τ_{xx}, τ_{yy} and τ_{xy}. We then calculate the second invariant of GPE for each area of the grid 100 times, $(T_{GPE})_{\mathrm{k}(\theta,\emptyset)}$. This allows us to calculate the variance and standard error of the second invariant of deviatoric stresses associated with GPE differences inside each cell of grid $(\theta, \emptyset)$ for each 0.5 myr time interval (k=1-73):

$$\mathrm{var}(T_{GPE})_{\mathrm{k}(\theta,\emptyset)} = \frac{1}{N-1} \sum_{i=1}^{N=100} (T_{GPE} - \bar{T}_{GPE})^2_{k(\theta,\emptyset)}$$

$$\mathrm{se}(T_{GPE})_{\mathrm{k}(\theta,\emptyset)} = \sqrt{var(T_{GPE})_{\mathrm{k}(\theta,\emptyset)}}$$

Supplementary Note 12

Discussion for Supplementary Figure 11: To calculate the standard error for iterative dynamic model stress and strain tensors we use 100 realized estimates of GPE fields in southwestern North America and perform the iterative dynamic modeling 100 times. This gives us 100 final estimates of τ_{xx}, τ_{yy} and τ_{xy} for deviatoric stresses, and 100 final estimates of $\dot{\epsilon}_{xx}$, $\dot{\epsilon}_{yy}$ and $\dot{\epsilon}_{xy}$ for strain rates produced in the iterative dynamic model. We then calculate the variance of τ_{xx}, τ_{yy}, τ_{xy}, $\dot{\epsilon}_{xx}$, $\dot{\epsilon}_{yy}$ and $\dot{\epsilon}_{xy}$ inside each cell of our grid $(\theta, \emptyset)$ at each time increment (k=1-73) as:

$$\mathrm{var}(\tau_{xx})_{\mathrm{k}(\theta,\emptyset)} = \frac{1}{N-1} \sum_{i=1}^{N=100} (\tau_{xx} - \bar{\tau}_{xx})^2_{k(\theta,\emptyset)}$$

$$\mathrm{var}(\tau_{yy})_{\mathrm{k}(\theta,\emptyset)} = \frac{1}{N-1} \sum_{i=1}^{N=100} (\tau_{yy} - \bar{\tau}_{yy})^2_{k(\theta,\emptyset)}$$

$$\mathrm{var}(\tau_{xy})_{\mathrm{k}(\theta,\emptyset)} = \frac{1}{N-1} \sum_{i=1}^{N=100} (\tau_{xy} - \bar{\tau}_{xy})^2_{k(\theta,\emptyset)}$$

$$\mathrm{var}(\dot{\epsilon}_{xx})_{\mathrm{k}(\theta,\emptyset)} = \frac{1}{N-1} \sum_{i=1}^{N=100} (\dot{\epsilon}_{xx} - \bar{\dot{\epsilon}}_{xx})^2_{k(\theta,\emptyset)}$$

$$\mathrm{var}(\dot{\epsilon}_{yy})_{\mathrm{k}(\theta,\emptyset)} = \frac{1}{N-1} \sum_{i=1}^{N=100} (\dot{\epsilon}_{yy} - \bar{\dot{\epsilon}}_{yy})^2_{k(\theta,\emptyset)}$$

$$\mathrm{var}(\dot{\epsilon}_{xy})_{\mathrm{k}(\theta,\emptyset)} = \frac{1}{N-1} \sum_{i=1}^{N=100} (\dot{\epsilon}_{xy} - \bar{\dot{\epsilon}}_{xy})^2_{k(\theta,\emptyset)}$$

Here, the bar over the symbols represents the average over 100 values. For the next step, we calculate the second invariant of dynamic model stress and strain rates for each area of the grid 100 times. This allows us

to calculate the variance and standard error of the second invariant of stress (T_{dyn}) and the variance and standard error of the second invariant of strain rates (E_{dyn}) as:

$$\text{var}(T_{dyn})_{k(\theta,\emptyset)} = \frac{1}{N-1}\sum_{i=1}^{N=100}(T_{dyn} - \bar{T}_{dyn})^2_{k(\theta,\emptyset)}$$

$$\text{se}(T_{dyn})_{k(\theta,\emptyset)} = \sqrt{var(T_{dyn})_{k(\theta,\emptyset)}}$$

$$\text{var}(E_{dyn})_{k(\theta,\emptyset)} = \frac{1}{N-1}\sum_{i=1}^{N=100}(E_{dyn} - \bar{E}_{dyn})^2_{k(\theta,\emptyset)}$$

$$\text{se}(E_{dyn})_{k(\theta,\emptyset)} = \sqrt{var(E_{dyn})_{k(\theta,\emptyset)}}$$

Supplementary Note 13

Discussion for Supplementary Figure 16: Once we have the variances for the second invariant of deviatoric stresses (T) and the second invariant of strain rates (E) from iterative dynamic model calculated inside each cell of our southwestern North America grid $(\theta, \emptyset)$ for each 0.5 myr time increment (k=1-73), we calculate the variance and standard error of effective viscosity of the lithosphere as:

$$\text{Var}(\eta)_{k(\theta,\emptyset)} = \text{var}(T.\tfrac{1}{E})_{k_{(\theta,\emptyset)}} =$$
$$[\boldsymbol{E}(T)_{k(\theta,\emptyset)}]^2\text{var}(\tfrac{1}{E})_{k_{(\theta,\emptyset)}} + [\boldsymbol{E}(\tfrac{1}{E})_{k_{(\theta,\emptyset)}}]^2.\text{var}(T)_{k(\theta,\emptyset)} + 2\boldsymbol{E}(T)_{k(\theta,\emptyset)}.\boldsymbol{E}(\tfrac{1}{E})_{k_{(\theta,\emptyset)}}.\text{cov}(T,\tfrac{1}{E})_{k_{(\theta,\emptyset)}} + \text{var}(T)_{k(\theta,\emptyset)}.\text{var}(\tfrac{1}{E})_{k_{(\theta,\emptyset)}} + \text{cov}^2(T,\tfrac{1}{E})_{k_{(\theta,\emptyset)}},$$

$$\text{se}(\eta)_{k(\theta,\emptyset)} = \sqrt{var(\eta)_{k(\theta,\emptyset)}}$$

where $\boldsymbol{E}$ denotes the expected values, $\text{var}\,(T)_{k(\theta,\emptyset)} = \frac{1}{N-1}\sum_{i=1}^{N=100}(T - \bar{T})^2_{k(\theta,\emptyset)}$, $\text{var}\,(\frac{1}{E})_{k_{(\theta,\emptyset)}} = \frac{1}{N-1}\sum_{i=1}^{N=100}(\frac{1}{E} - \overline{\frac{1}{E}})^2_{k(\theta,\emptyset)}$, and $\text{cov}(T,\frac{1}{E})_{k_{(\theta,\emptyset)}} = \frac{1}{N-1}\sum_{i=1}^{N=100}(T - \bar{T})_{k(\theta,\emptyset)} \cdot (\frac{1}{E} - \overline{\frac{1}{E}})_{k_{(\theta,\emptyset)}}$.

Supplementary Figures

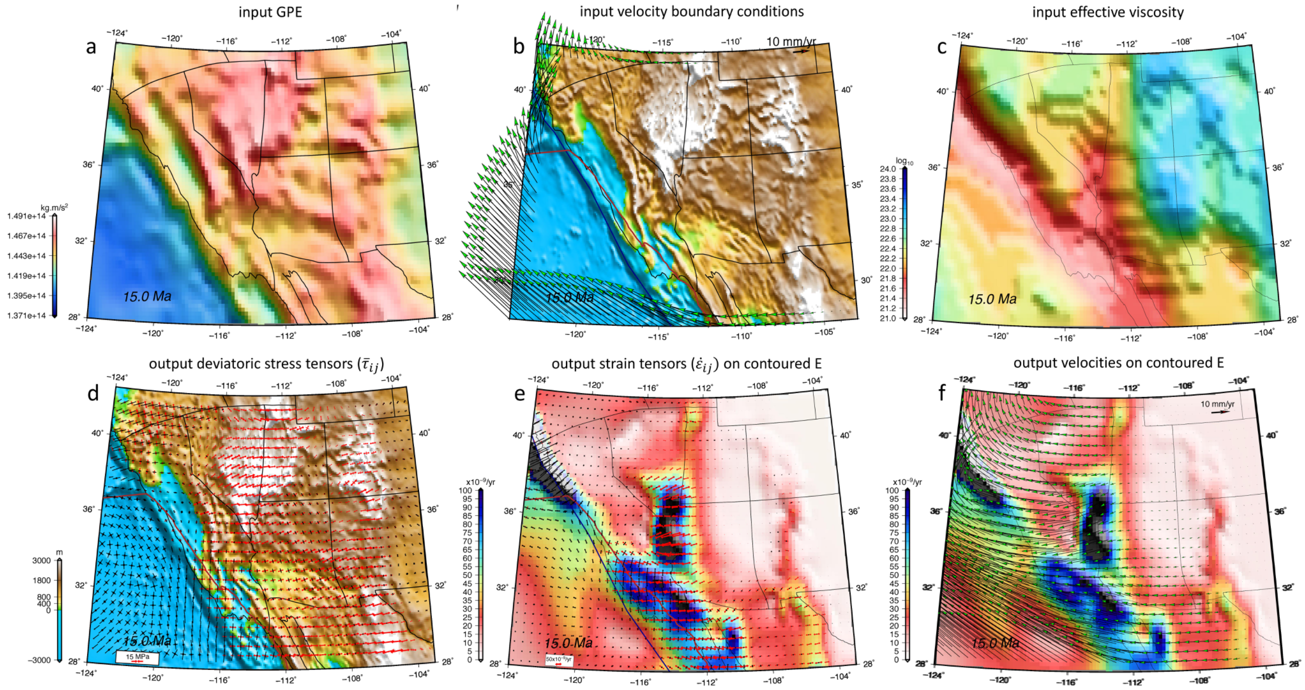

Supplementary Fig. 1 | Inputs (a-c) and outputs (d-f) of geodynamic calculations for benchmarking at 15 Ma. a. Map of contoured GPE estimates; **b.** Boundary condition velocity estimates based on accommodation of Farallon- and Pacific-North America relative plate motions in southwestern North America; **c.** Map of lithospheric effective viscosity that must be recovered in benchmarking; **d.** Map of principal axes of lithospheric deviatoric stress tensors obtained in solution to force balance using inputs in **a-c**, achieved through minimization of (11) in Methods or (5) below. Red arrows represent tensional, and black arrows represent compressional principal axes of deviatoric stresses; **e.** Map of contoured second invariant of strain rates (E) and principal axes of strain tensors obtained from force-balance solution with inputs from **a-c**. Red arrows represent tensional, and black arrows represent compressional principal axes of strain rates; **f.** Map of contoured second invariant of strain rates (E) and model velocities relative to the North American frame achieved from force balance solution from inputs in **a-c**.

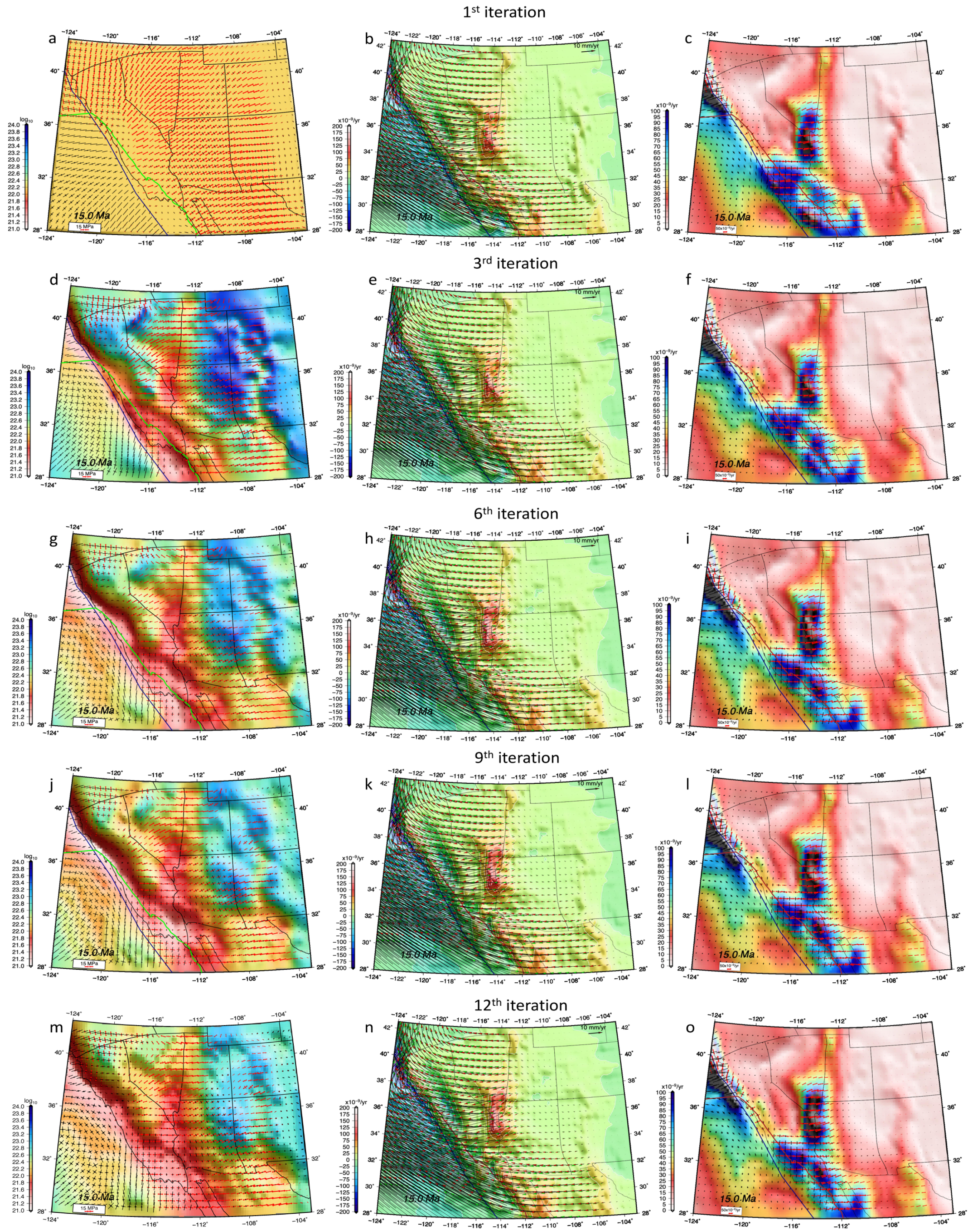

Supplementary Fig. 2 | Blind test benchmarking. a-c. Dynamically constrained approximation of principal axes of vertically averaged horizontal deviatoric stresses associated with GPE gradients based on an initially constant viscosity value for the lithosphere (a), model dilatational strain rates and model velocities relative to the North American frame (b) and contoured second invariant of strain rates (*E*) and principal axes of strain tensors (c) achieved from **1st** iteration; **d-f.** Dynamically constrained approximation of lithospheric effective viscosity and principal axes of lithospheric deviatoric stress tensors (d), model dilatational strain rates and model velocities relative to the North American frame (e) and contoured second invariant of strain rates (*E*) and principal axes of strain tensors (f) achieved from **3rd** iteration; **g-i.** Same as "**d-f**" but for **6th** iteration; **j-l.** Same as "**d-f**" but for **9th** iteration; **m-o.** Same as "**d-f**" but for **12th** iteration. Red arrows represent tensional, and black arrows represent compressional principal axes of strain rate.

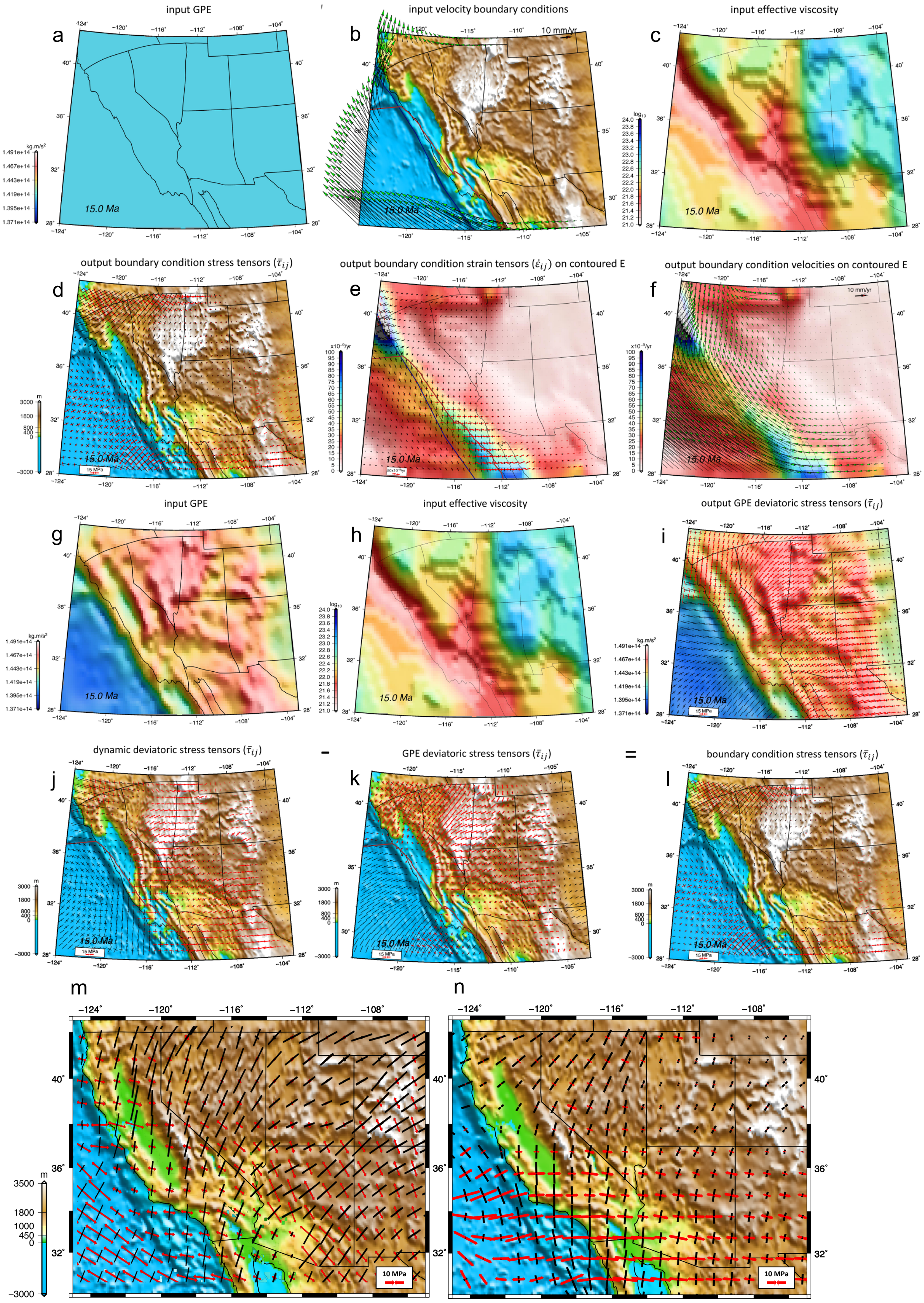
input GPE
input velocity boundary conditions
input effective viscosity
output boundary condition stress tensors ($\bar{\tau}_{ij}$)
output boundary condition strain tensors ($\dot{\varepsilon}_{ij}$) on contoured E
output boundary condition velocities on contoured E
input GPE
input effective viscosity
output GPE deviatoric stress tensors ($\bar{\tau}_{ij}$)
dynamic deviatoric stress tensors ($\bar{\tau}_{ij}$)
GPE deviatoric stress tensors ($\bar{\tau}_{ij}$)
boundary condition stress tensors ($\bar{\tau}_{ij}$)
15.0 Ma
10 mm/yr
15 MPa
10 MPa

Supplementary Fig. 3 | Inputs (a-c) and outputs for (d-f) geodynamically constrained boundary conditions at 15 Ma. a. Map of an initially constant GPE value for the lithosphere; **b.** Boundary condition velocity estimates based on accommodation of Farallon- and Pacific-North America relative plate motions in southwestern North America; **c.** Map of lithospheric effective viscosity obtained in final iterative dynamic model; **d.** Map of principal axes of lithospheric boundary condition stress tensors. Red arrows represent tensional, and black arrows represent compressional principal axes of stresses; **e.** Map of contoured second invariant of boundary condition strain rates (E) and principal axes of boundary condition strain tensors. Red arrows represent tensional, and black arrows represent compressional principal axes of strain rates; **f.** Map of contoured second invariant of boundary condition strain rates (E) and model boundary condition velocities relative to the North American frame; **g.** Map of contoured GPE estimates; **h.** Map of lithospheric effective viscosity obtained in final iterative dynamic model; **i.** Map of principal axes of deviatoric stress tensors associated with GPE (contoured) gradients, obtained using estimates of laterally varying effective viscosity from final iterative dynamic model; **j.** Map of principal axes of lithospheric deviatoric stress tensors obtained in final iterative dynamic model. Red arrows represent tensional, and black arrows represent compressional principal axes of deviatoric stresses; **k.** Map of principal axes of deviatoric stress tensors associated with GPE gradients, obtained using estimates of laterally varying effective viscosity from final iterative dynamic model; **l.** Map of principal axes of lithospheric boundary condition stress tensors obtained by subtracting stress values in "e" from stress values in "d". Red arrows represent tensional, and black arrows represent compressional principal axes of deviatoric stresses. Comparison of solutions using the two methods shows that they are nearly identical; **m.** Deviatoric stresses from lithosphere coupling with global mantle flow S40RTS[16,17]; **n.** Boundary condition solution of deviatoric stresses at present-day (Matching the compressional directions and magnitudes in both models within the Basin and Range Province can emphasize the long-wavelength effect of mantle flow).

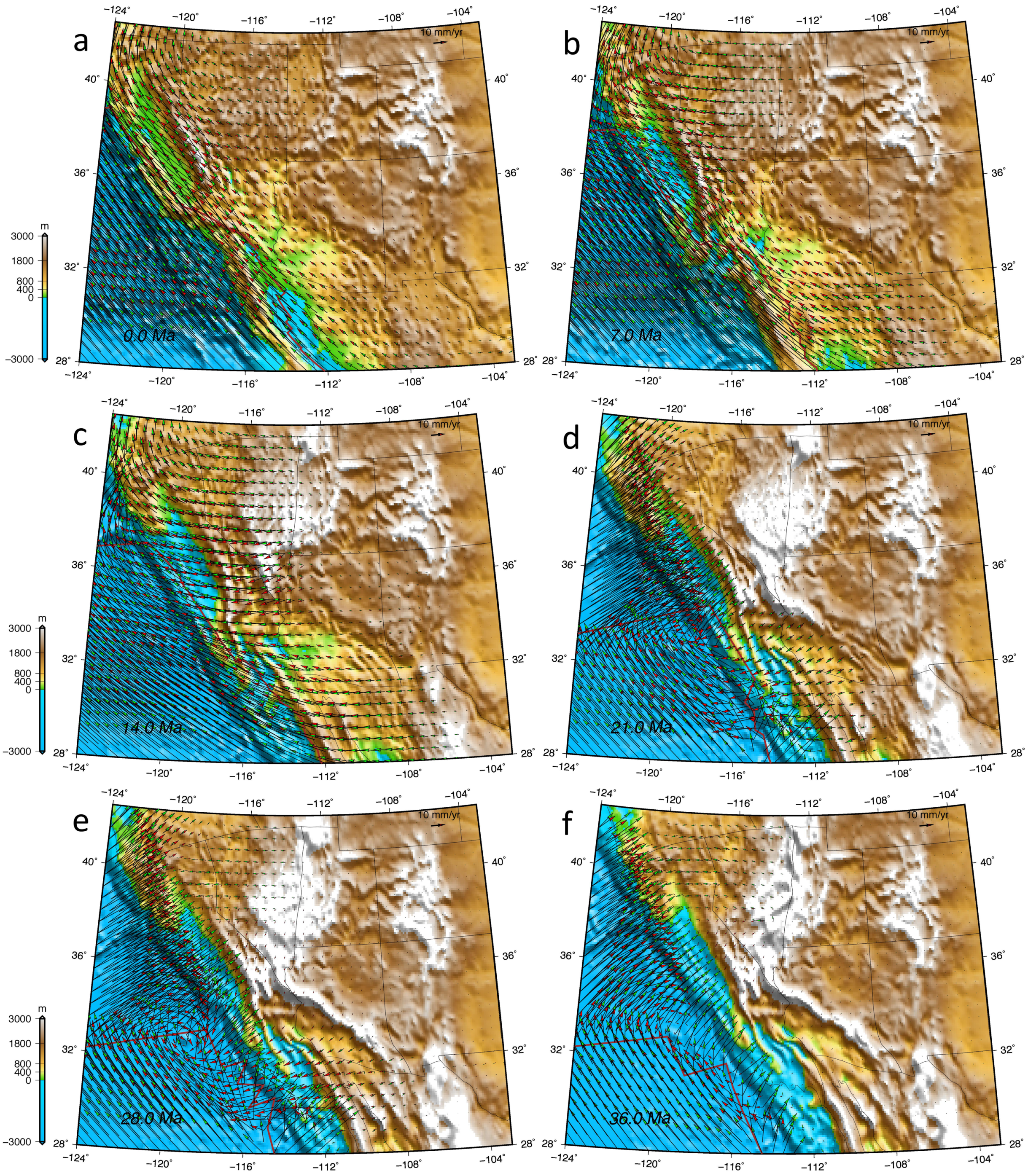

Supplementary Fig. 4 | a-f. Time-slice maps of southwestern North America velocity field estimate relative to the North American frame. Red vectors are predicted velocity field from iterative dynamic model and green vectors are model velocity field from kinematic solution. Brown line shows the evolution of Ridge and SAFS through time.

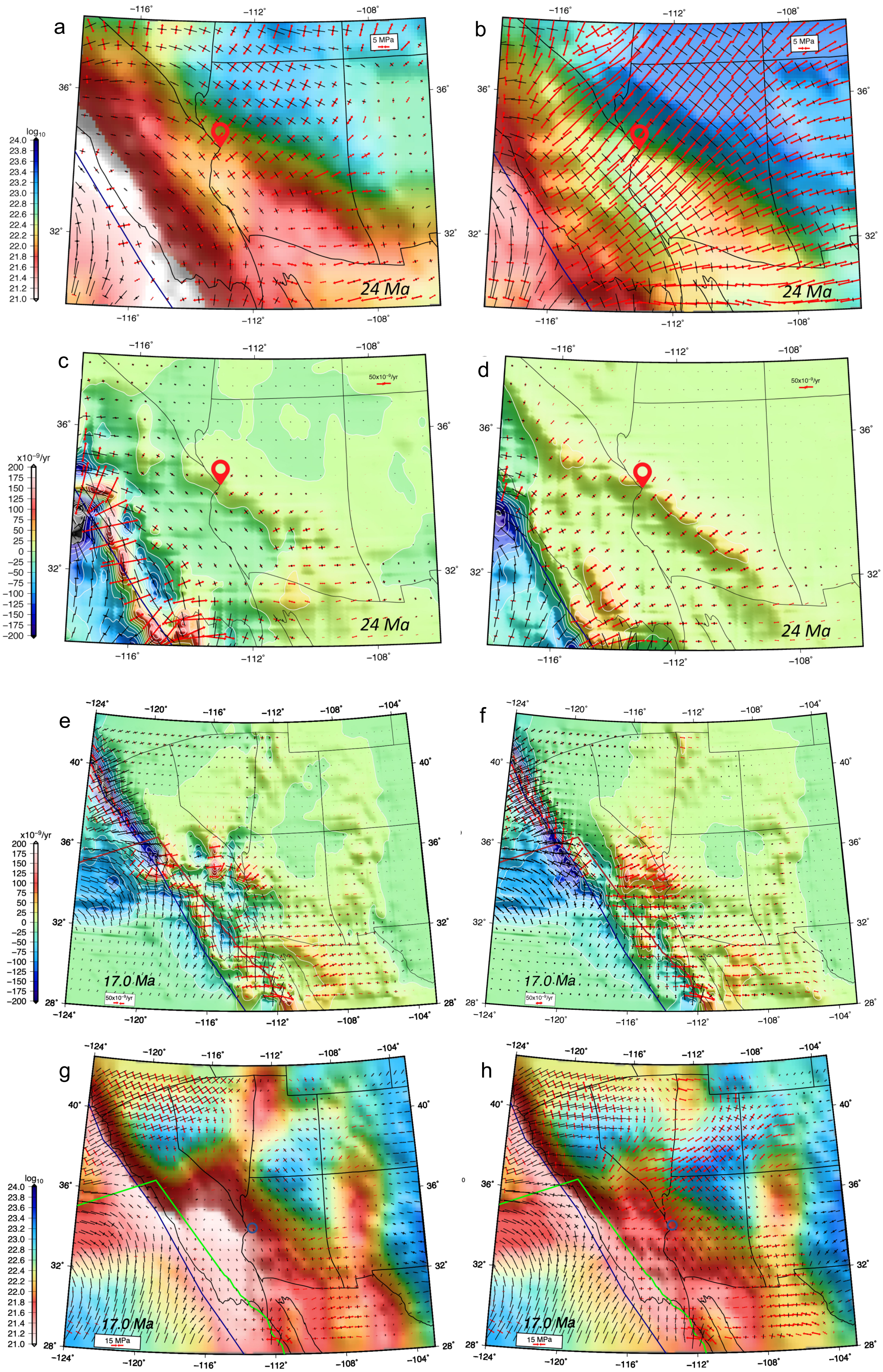
a
b
c
d
e
f
g
h
24 Ma
24 Ma
24 Ma
24 Ma
17.0 Ma
17.0 Ma
17.0 Ma
17.0 Ma
5 MPa
5 MPa
50x10⁻⁹/yr
15 MPa
log10
x10⁻⁹/yr

Supplementary Fig. 5 | a. Map of lithospheric effective viscosity obtained from deviatoric stresses of iterative dynamic model, kinematic strain rates and GPE estimates that does not include a Mogollon Highlands at 24 Ma. Red arrows represent tensional, and black arrows represent compressional principal axes of stress tensors; **b.** Map of lithospheric effective viscosity obtained from deviatoric stresses of iterative dynamic model, kinematic strain rates and GPE estimates that include a Mogollon Highlands at 24 Ma; **c.** Map of dilatational strain rates achieved from iterative dynamic model and GPE estimates that does not include a Mogollon Highlands at 24 Ma. Red arrows represent tensional, and black arrows represent compressional principal axes of strain tensors; **d.** Map of dilatational strain rates achieved from iterative dynamic model and GPE estimates that includes a Mogollon Highlands at 24 Ma**; e.** Map of dilatational strain rates achieved from iterative dynamic model and GPE estimates that are missing a Mogollon Highlands in northwestern Arizona and southernmost Nevada at 17 Ma. Red arrows represent tensional, and black arrows represent compressional principal axes of strain rates; **f.** Map of dilatational strain rates achieved from iterative dynamic model and GPE estimates that includes a Mogollon Highlands in northwestern Arizona and southernmost Nevada at 17 Ma; **g.** Map of lithospheric effective viscosity obtained from deviatoric stresses of iterative dynamic model, kinematic strain rates and GPE estimates that are missing the high topography of Mogollon Highlands in northwestern Arizona and southernmost Nevada at 17 Ma. Blue dot represents the reconstructed position of Whipple Mountain MCC; **h.** Map of lithospheric effective viscosity obtained from deviatoric stresses of iterative dynamic model, kinematic strain rates, and GPE estimates that include a high topography of Mogollon Highlands in northwestern Arizona and southernmost Nevada at 17 Ma.

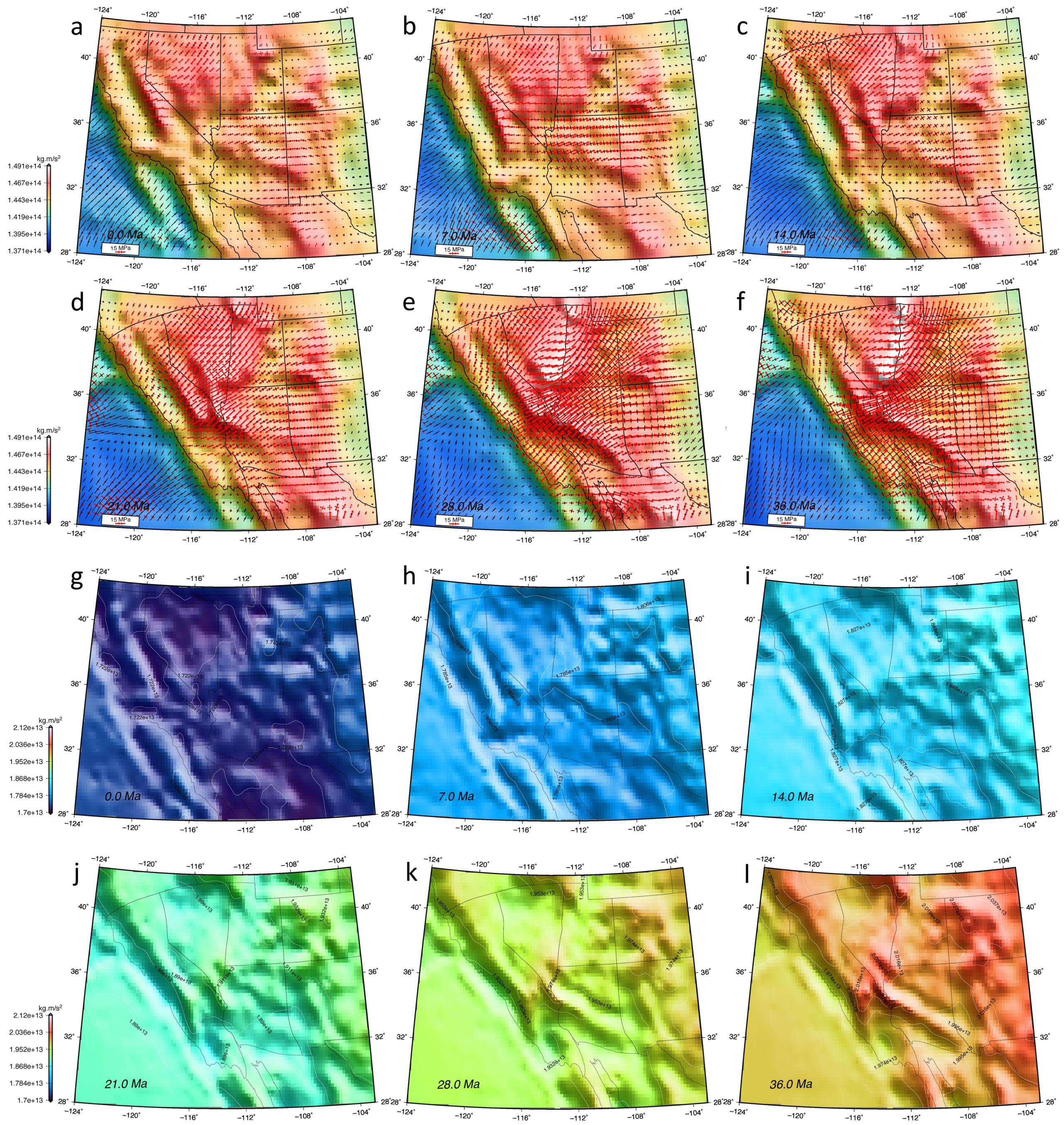

Supplementary Fig. 6 | a-f. Time-slice maps deviatoric stresses associated with gravitational potential energy (GPE) variations using laterally varying effective viscosity values for the lithosphere achieved from iterative dynamic model. The stress field associated with GPE differences is obtained by solving the force balance equations, assuming the lithosphere is in isostatic balance at 100 km depth. Red arrows represent tensional principal axes of deviatoric stress, and black arrows represent compressional principal axes of deviatoric stress. **g-l.** Time-slice maps of GPE standard errors in North American plate through time.

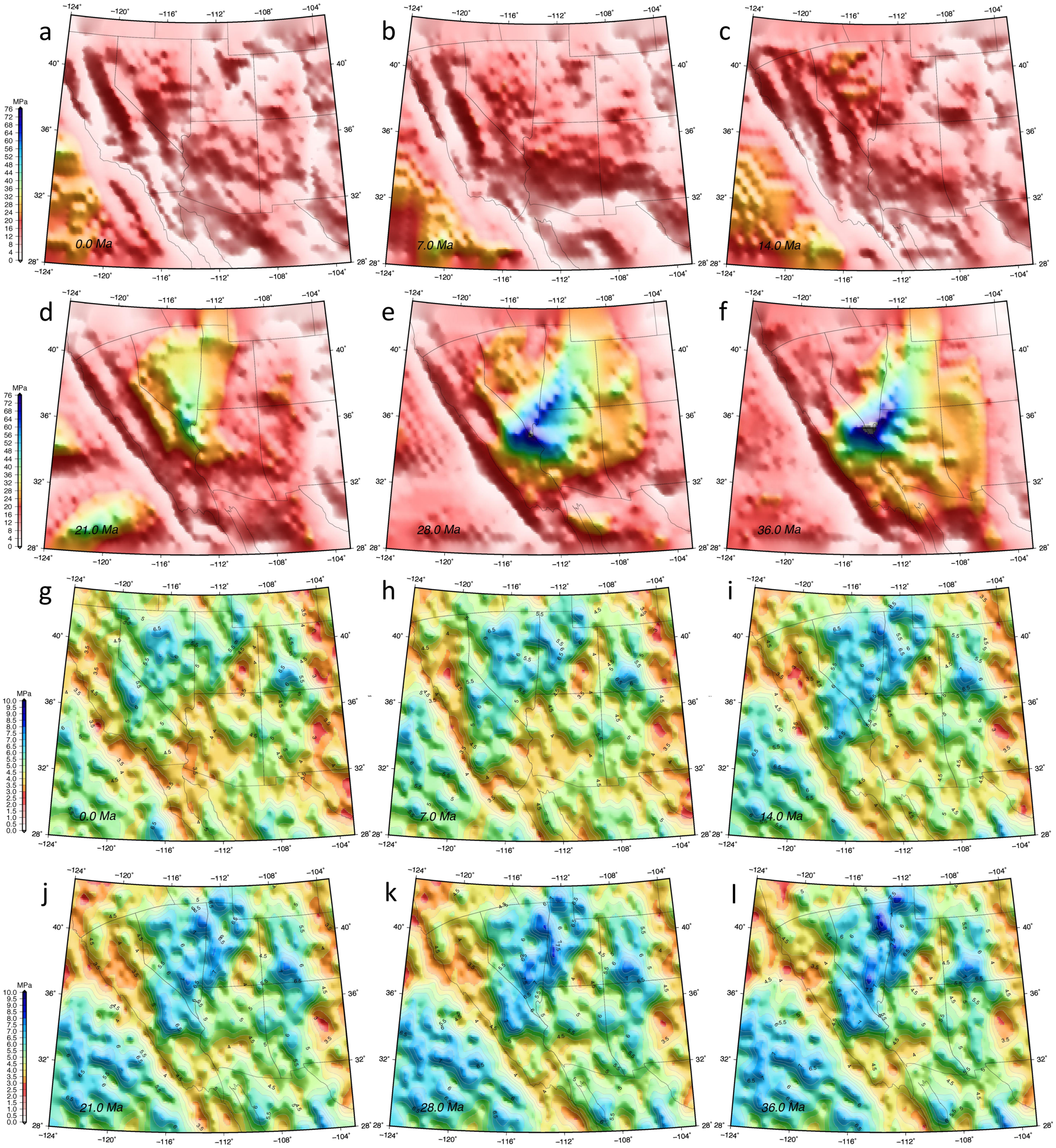

Supplementary Fig. 7 | a-f. Time-slice maps of the second invariant of vertically averaged deviatoric stresses from GPE differences (T_{GPE}) in southwestern North America. **g-l.** Time-slice maps of standard errors for T_{GPE} in North American plate.

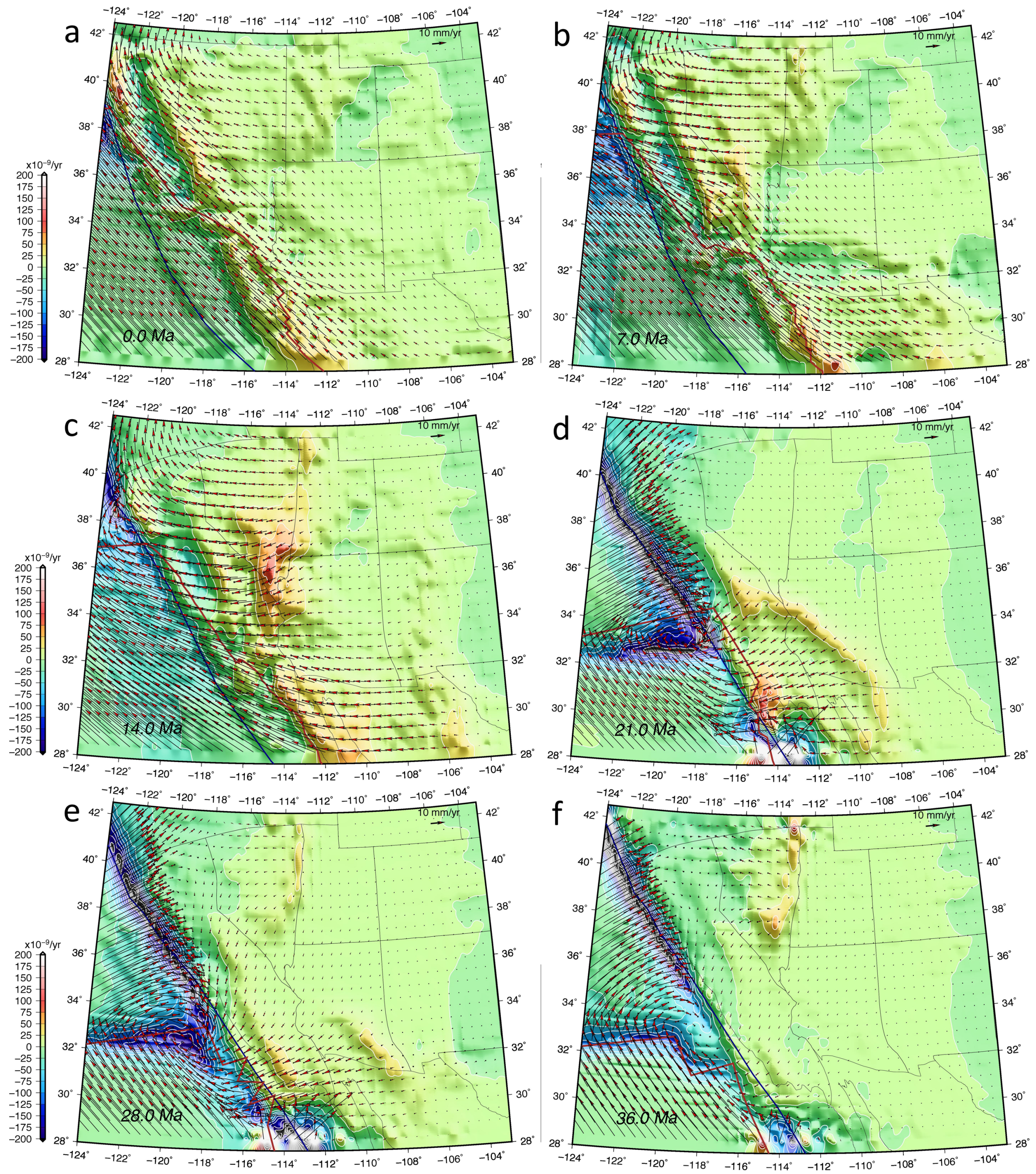

Supplementary Fig. 8 | a-f. Time-slice maps of contoured dilatation strain rates obtained from iterative dynamic model. Red vectors are predicted velocity fields from iterative dynamic model relative to the North American frame.

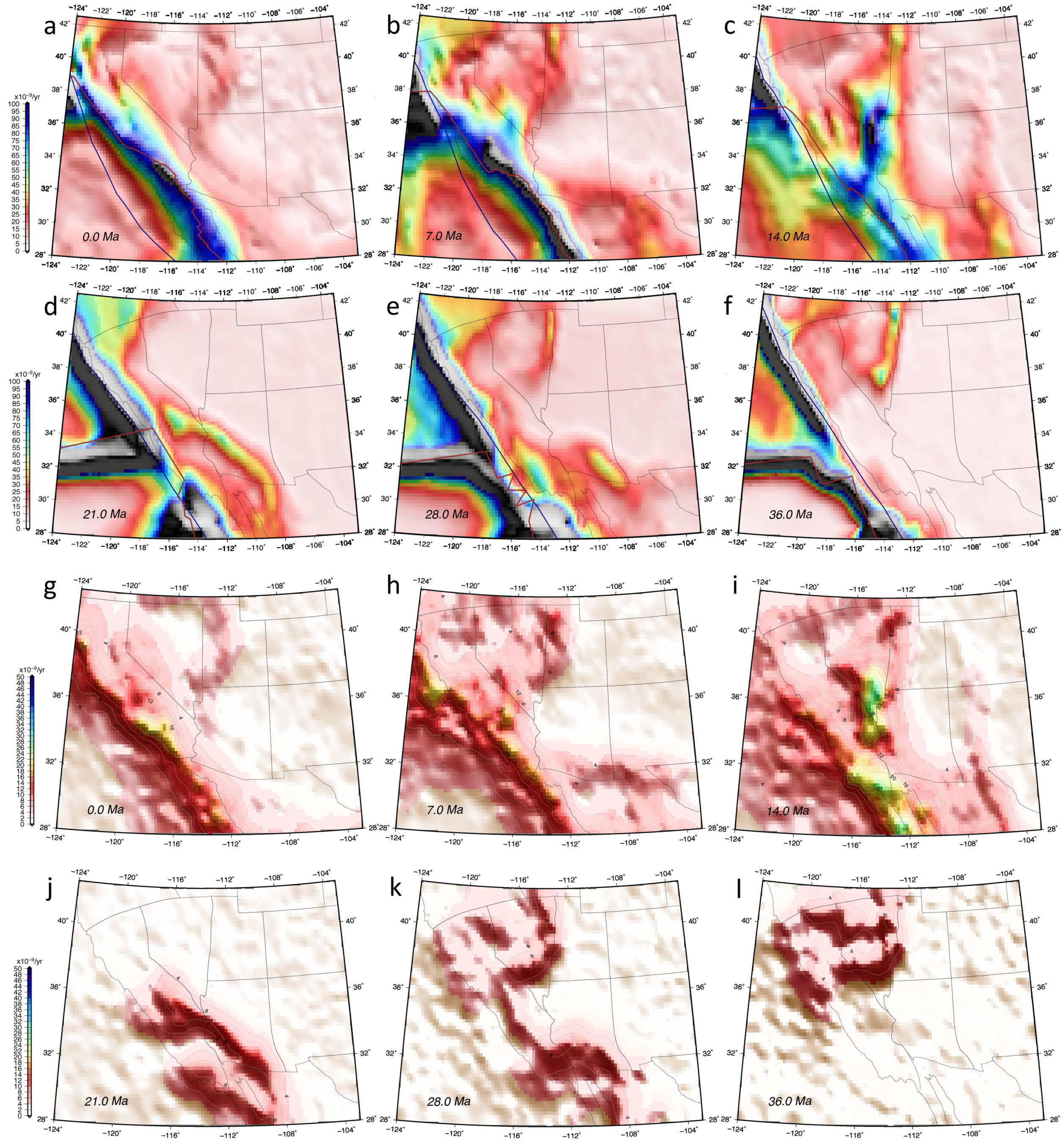

Supplementary Fig. 9 | a-f. Time-slice maps of the second invariant of strain rates (E) from iterative dynamic model in southwestern North America. **g-l.** Time-slice maps of standard errors for E in North American plate from iterative dynamic model.

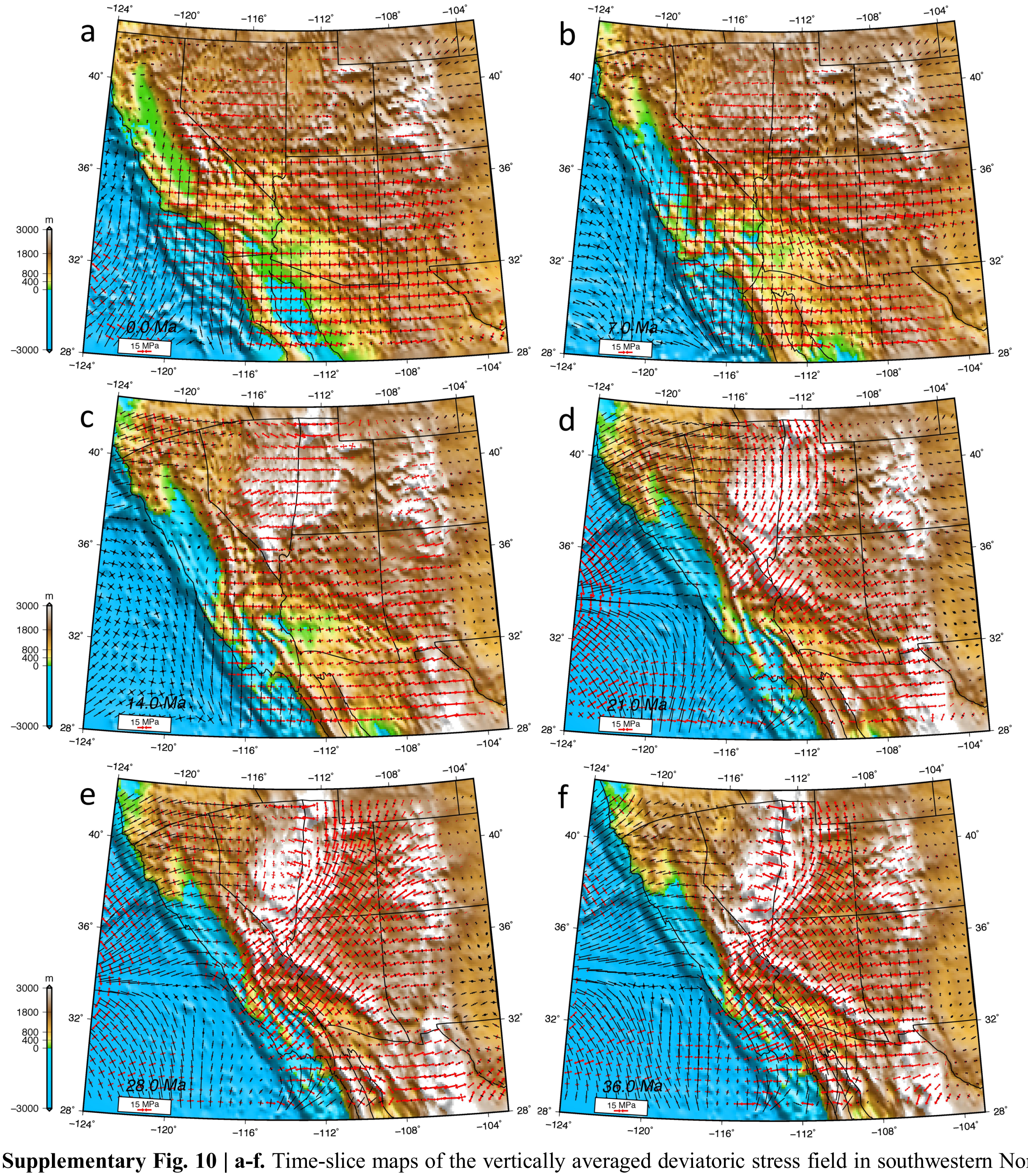

Supplementary Fig. 10 | a-f. Time-slice maps of the vertically averaged deviatoric stress field in southwestern North America obtained from iterative dynamic model. Red arrows represent tensional principal axes of deviatoric stress, and black arrows represent compressional principal axes of deviatoric stress.

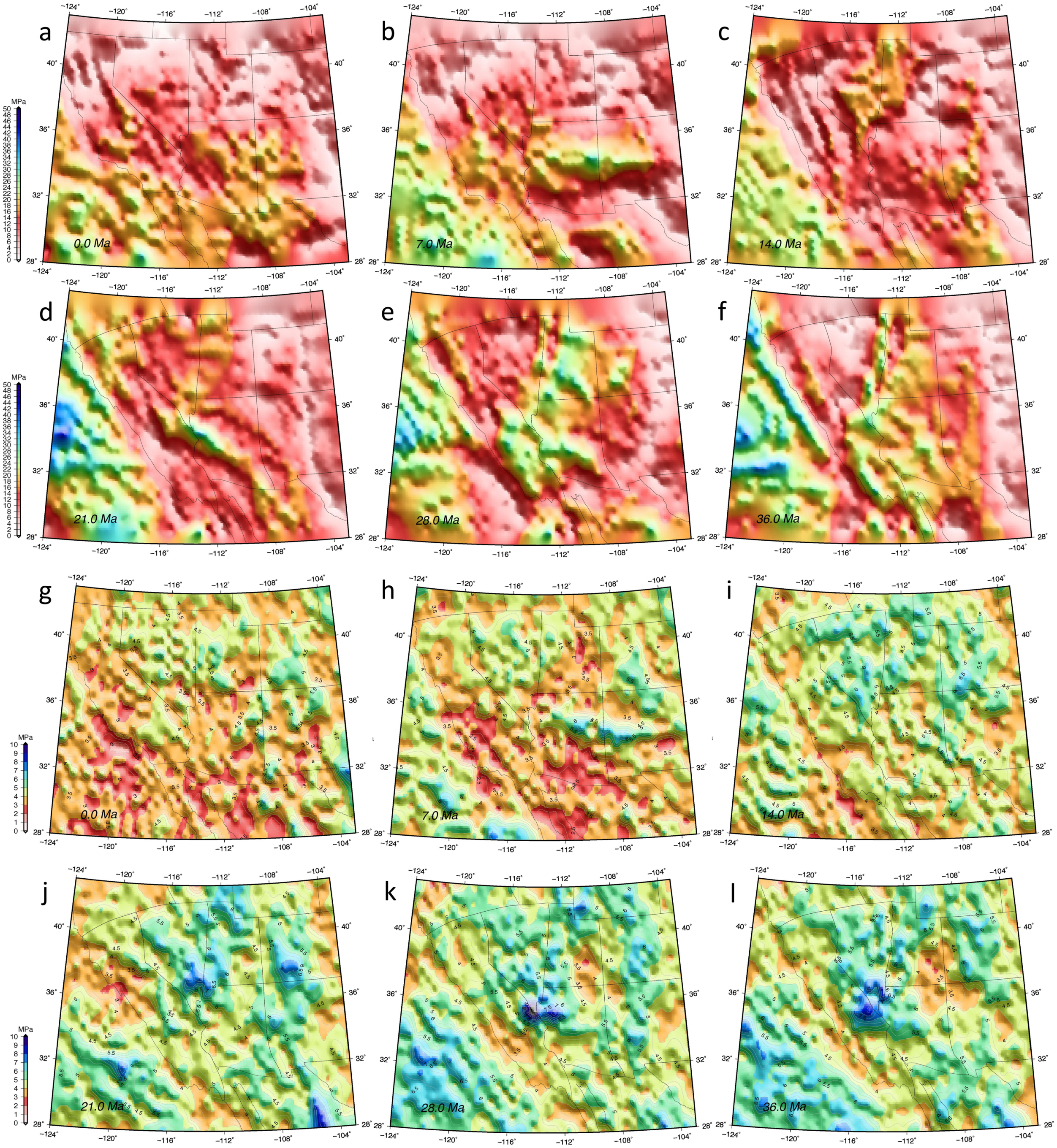

Supplementary Fig. 11 | a-f. Time-slice maps of the second invariant of vertically averaged deviatoric stress fields (T_{dyn}) from iterative dynamic model in southwestern North America. **g-l.** Time-slice maps of standard errors for T_{dyn} in North American plate from iterative dynamic model.

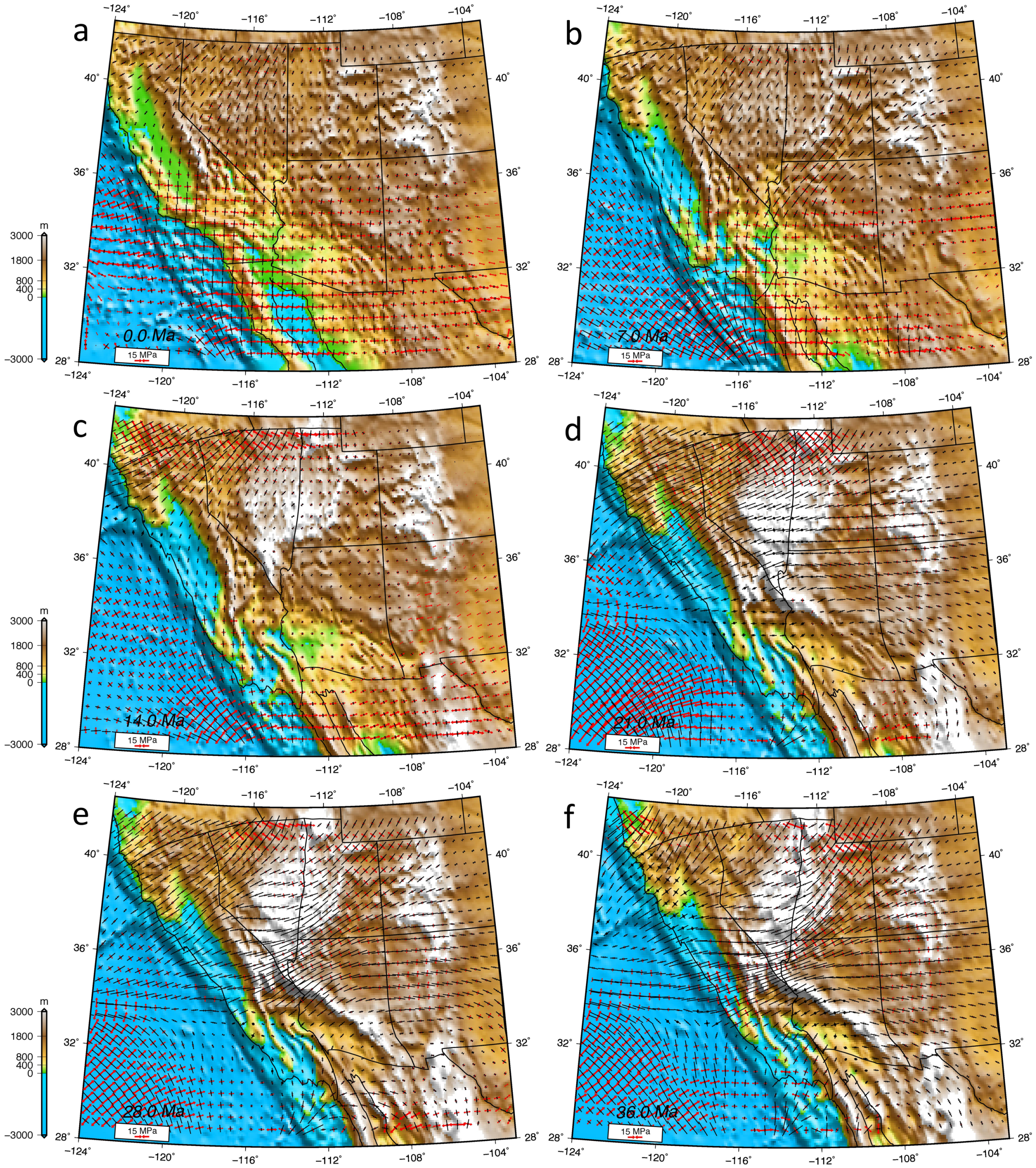

Supplementary Fig. 12 | a-f. Time-slice maps of the stress field boundary conditions in southwestern North America. Red arrows represent tensional principal axes of deviatoric stress, and black arrows represent compressional principal axes of deviatoric stress.

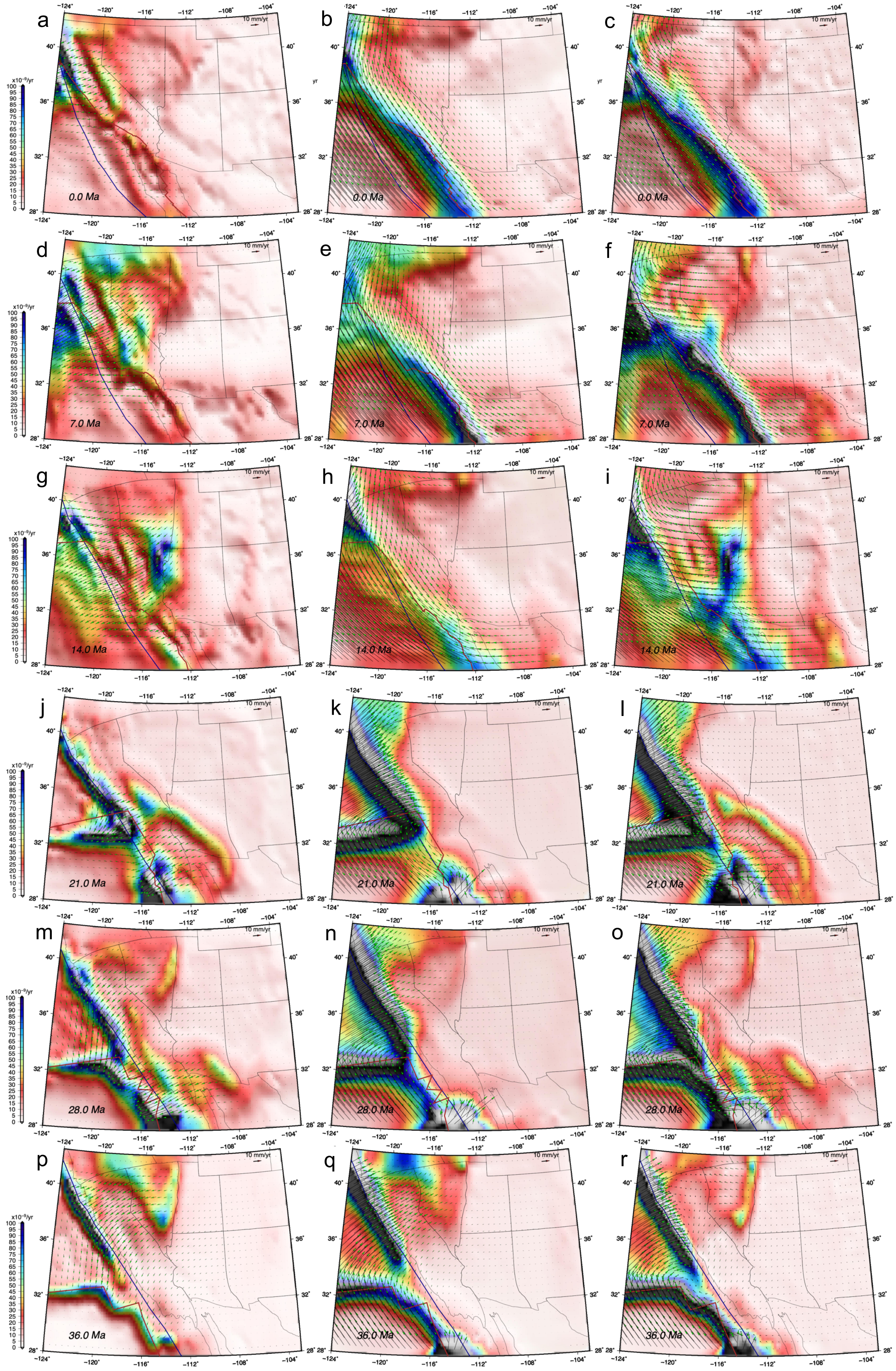
a
b
c
0.0 Ma
d
e
f
7.0 Ma
g
h
i
14.0 Ma
j
k
l
21.0 Ma
m
n
o
28.0 Ma
p
q
r
36.0 Ma
10 mm/yr
x10⁻⁹/yr

Supplementary Fig. 13 | a. Map of contoured second invariant of strain rates (*E*) and velocity field estimates relative to the North American frame associated with GPE gradients alone, achieved by subtracting boundary conditions solution from the complete solution obtained in iterative dynamic model, which includes the combination of velocities and strain tensors associated with GPE gradients and the relative plate motion at present-day; **b.** Map of contoured boundary conditions second invariant of strain rates (*E*) and boundary conditions velocity field estimates at present-day; **c.** Map of contoured second invariant of strain rates (*E*) and velocity field estimates relative to the North American frame from full iterative dynamic model at present-day; **d-f.** same as "a-c" but at 7 Ma; **g-i.** same as "a-c" but at 14 Ma; **j-l.** same as "a-c" but at 21 Ma; **m-o.** same as "a-c" but at 28 Ma; **p-r.** same as "a-c" but at 36 Ma.

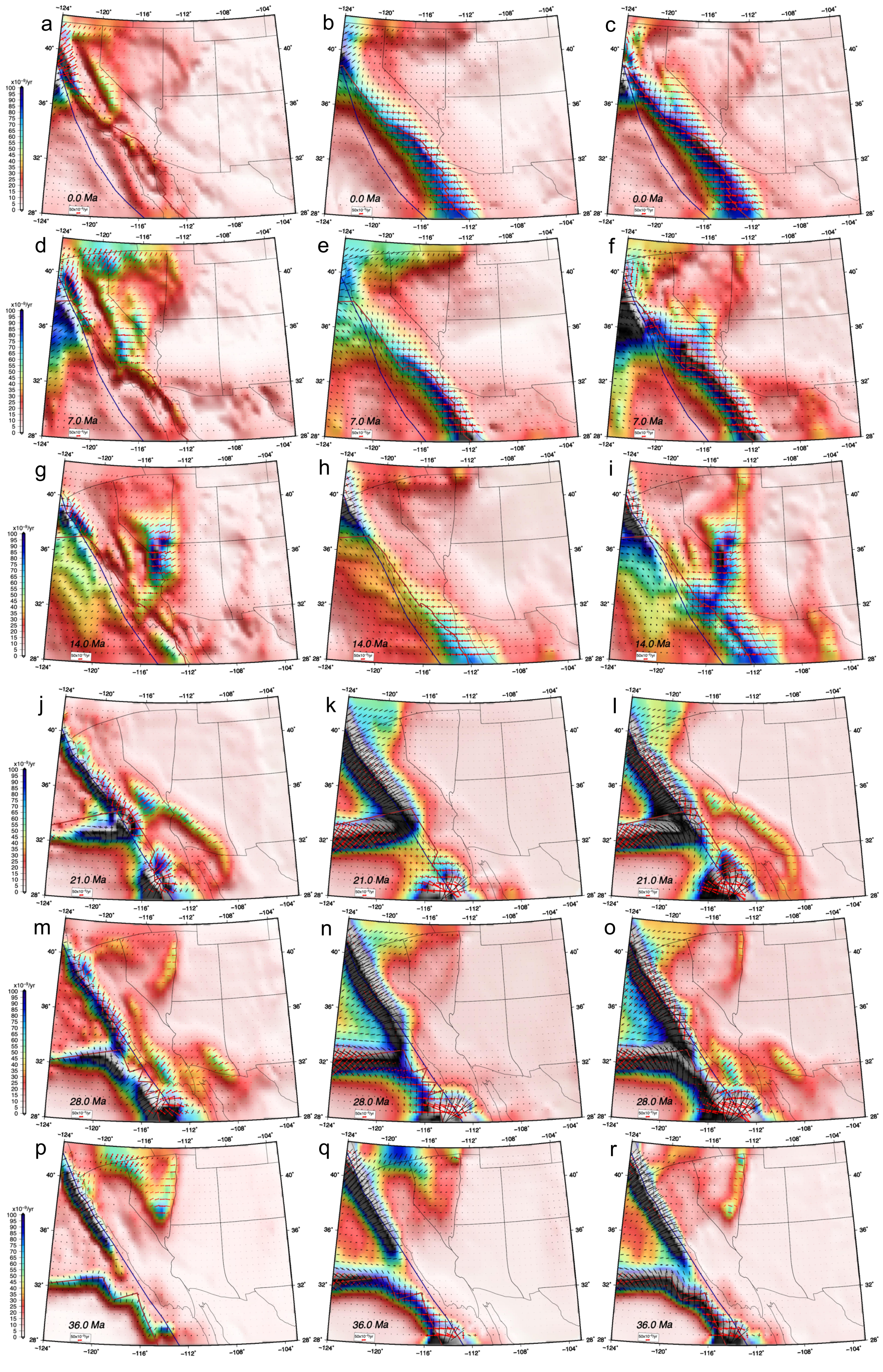

a
b
c
0.0 Ma
d
e
f
7.0 Ma
g
h
i
14.0 Ma
j
k
l
21.0 Ma
m
n
o
28.0 Ma
p
q
r
36.0 Ma
x10^{-9}/yr
-124°
-120°
-116°
-112°
-108°
-104°
28°
32°
36°
40°

Supplementary Fig. 14 | a. Map of contoured second invariant of strain rates (*E*) and principal axes of strain tensors associated with GPE gradients alone, obtained by subtracting boundary condition solution from complete solution obtained in iterative dynamic model, which includes the combination of *E* and strain tensors associated with GPE gradients and the relative plate motion at present-day; **b.** Map of *E* and principal axes of boundary conditions strain tensors at present-day; **c.** Map of contoured *E* and principal axes of strain tensors obtained in final iterative dynamic model at present-day; **d-f.** same as "a-c" but at 7 Ma; **g-i.** same as "a-c" but at 14 Ma; **j-l.** same as "a-c" but at 21 Ma; **m-o.** same as "a-c" but at 28 Ma; **p-r.** same as "a-c" but at 36 Ma.

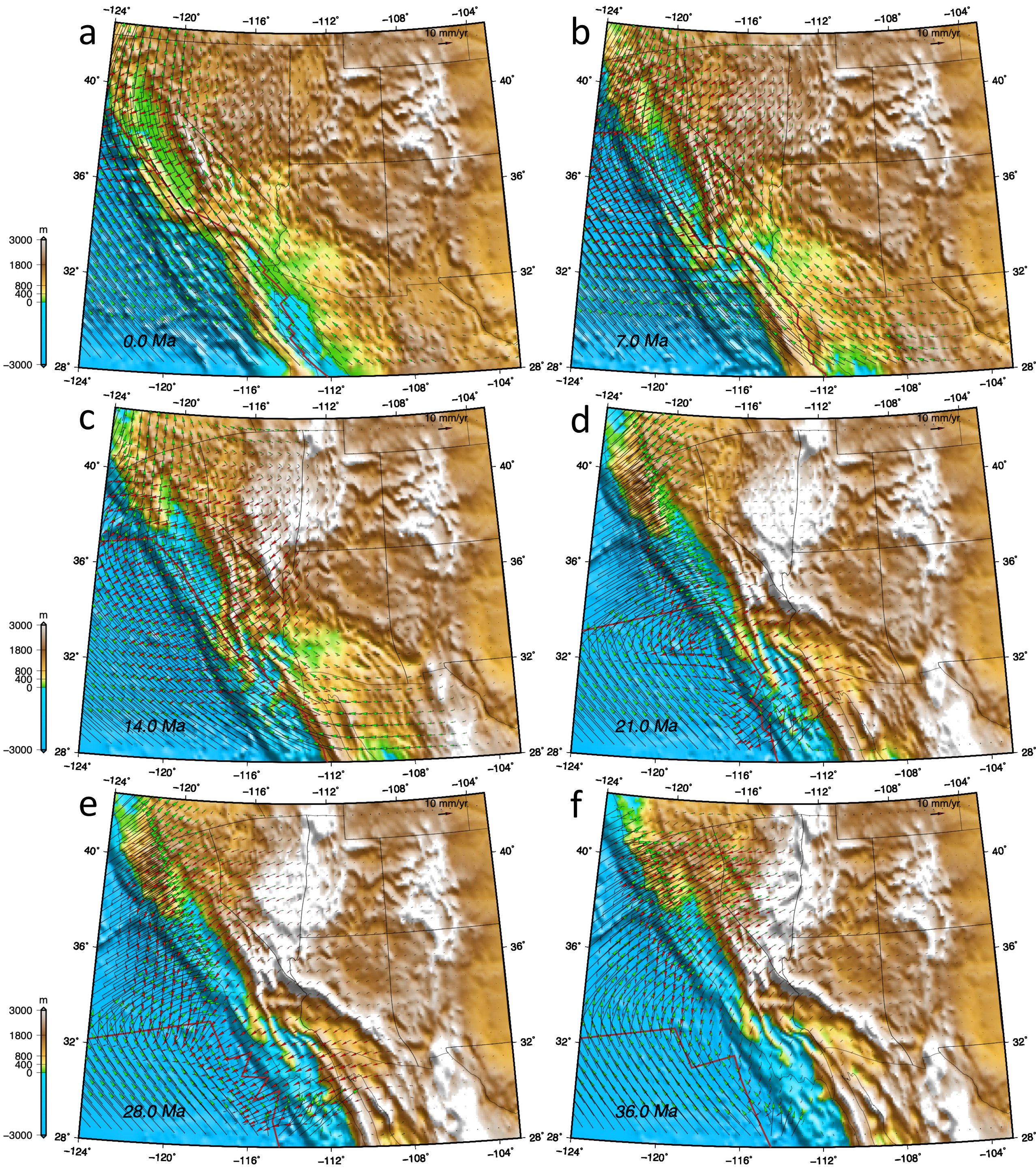

Supplementary Fig. 15 | a-f. Time-slice maps of southwestern North America velocity field estimate relative to the North American frame. Red vectors are velocity field achieved by subtracting boundary condition velocities from the final estimates of surface kinematics from iterative dynamic model. Green vectors are boundary condition velocity field estimates. Brown line shows the evolution of Ridge and SAFS through time.

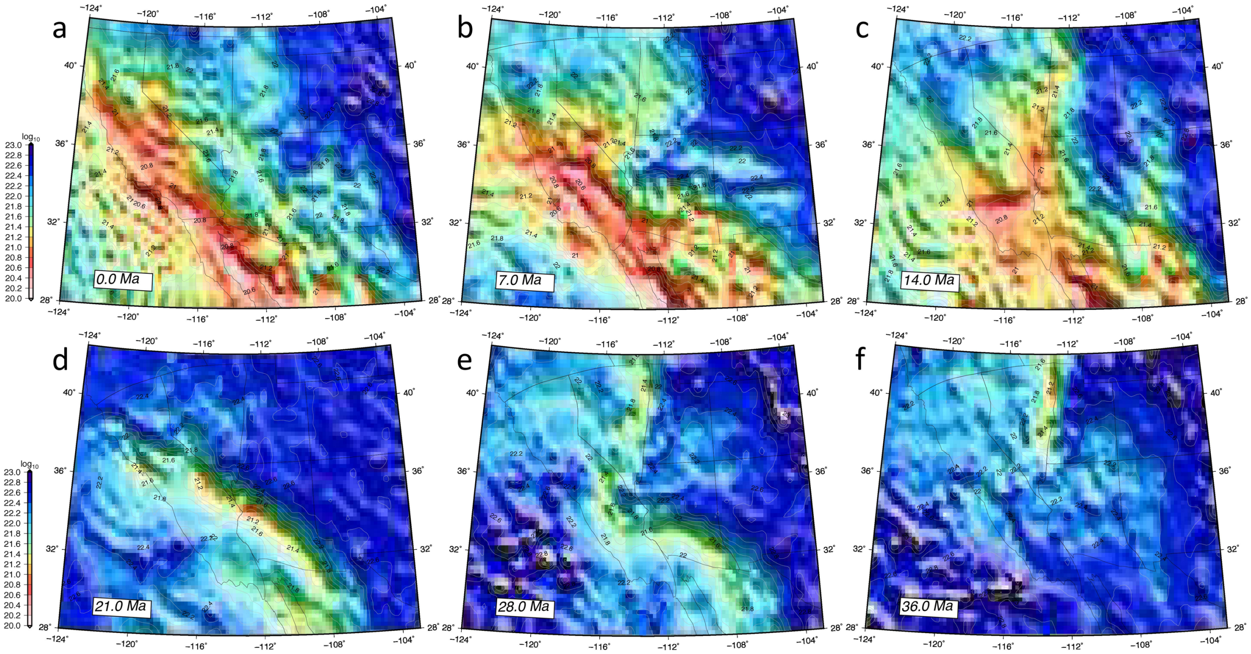

Supplementary Fig. 16 | a-f. Time-slice maps of standard errors for lithospheric effective viscosity in North American plate, obtained by running iterative dynamic model 100 times using 100 realized GPE fields in southwestern North America.

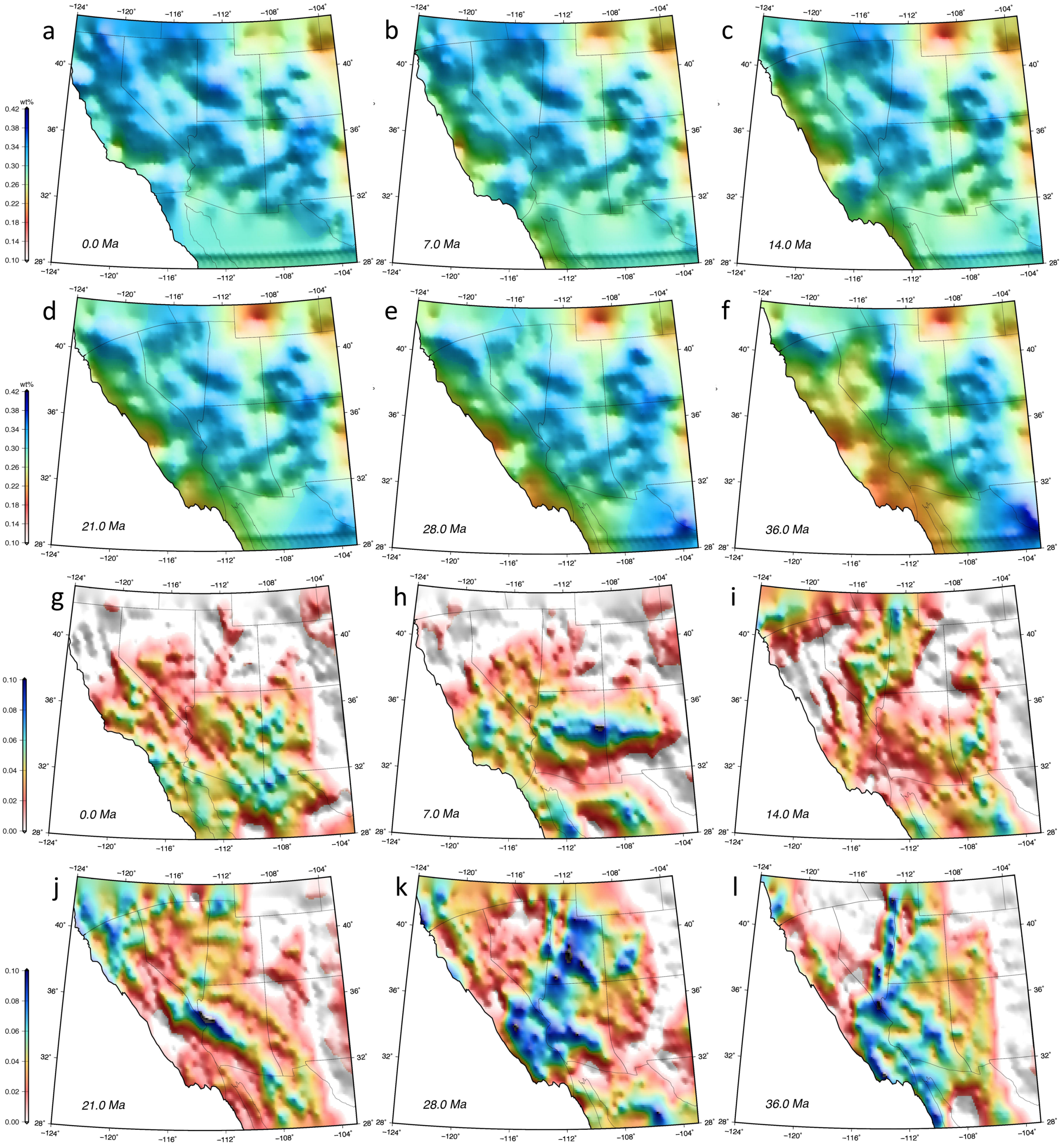

Supplementary Fig. 17 | a-f. Time-slice maps of volumetric degree of melting (M_0) of the lithosphere in southwestern North America; **g-l.** Time-slice maps of melt and fluid pressure factor (λ) for a partially molten wet upper mantle (wet olivine) using the method of Gerya and Meilick[18,19].

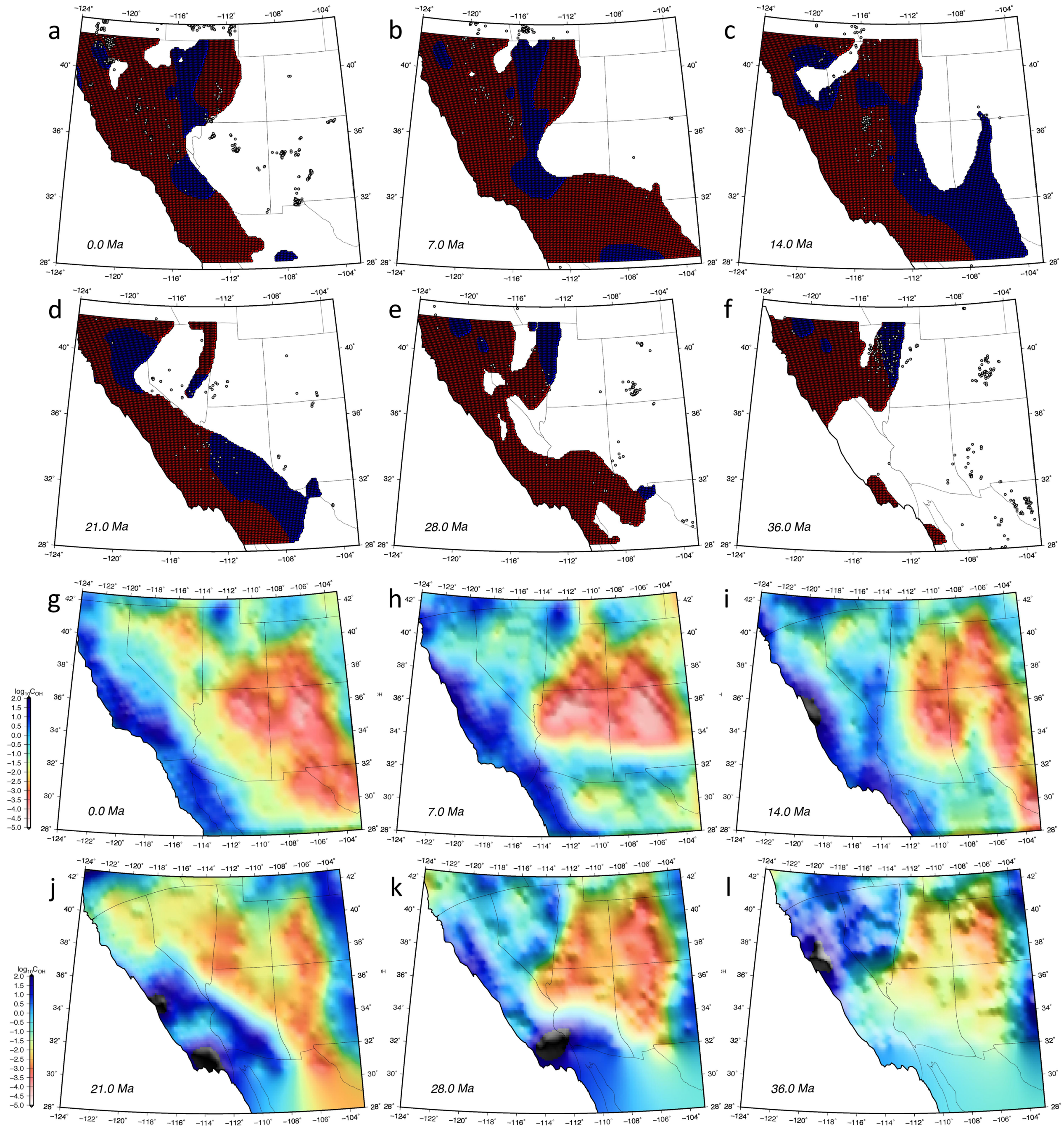

Supplementary Fig. 18 | a-f. Time-slice maps of molten (red areas) and non-molten (blue areas) phases of the lithosphere in southwestern North America within areas affected by active crustal deformation (second invariant of strain rates > 0). Grey dots represent the reconstructed potion of present-day coordinates of paleo-magmatism in Southwestern North America; **g-l.** Time-slice maps of C_{OH} achieved using a power law creep rheological model based on method of Dixon et al.[20] for distributed water in nominally anhydrous minerals like olivine. Note the close correlation of high C_{OH} zones and independently estimated non-molten phases (blue zone in panels a-f).

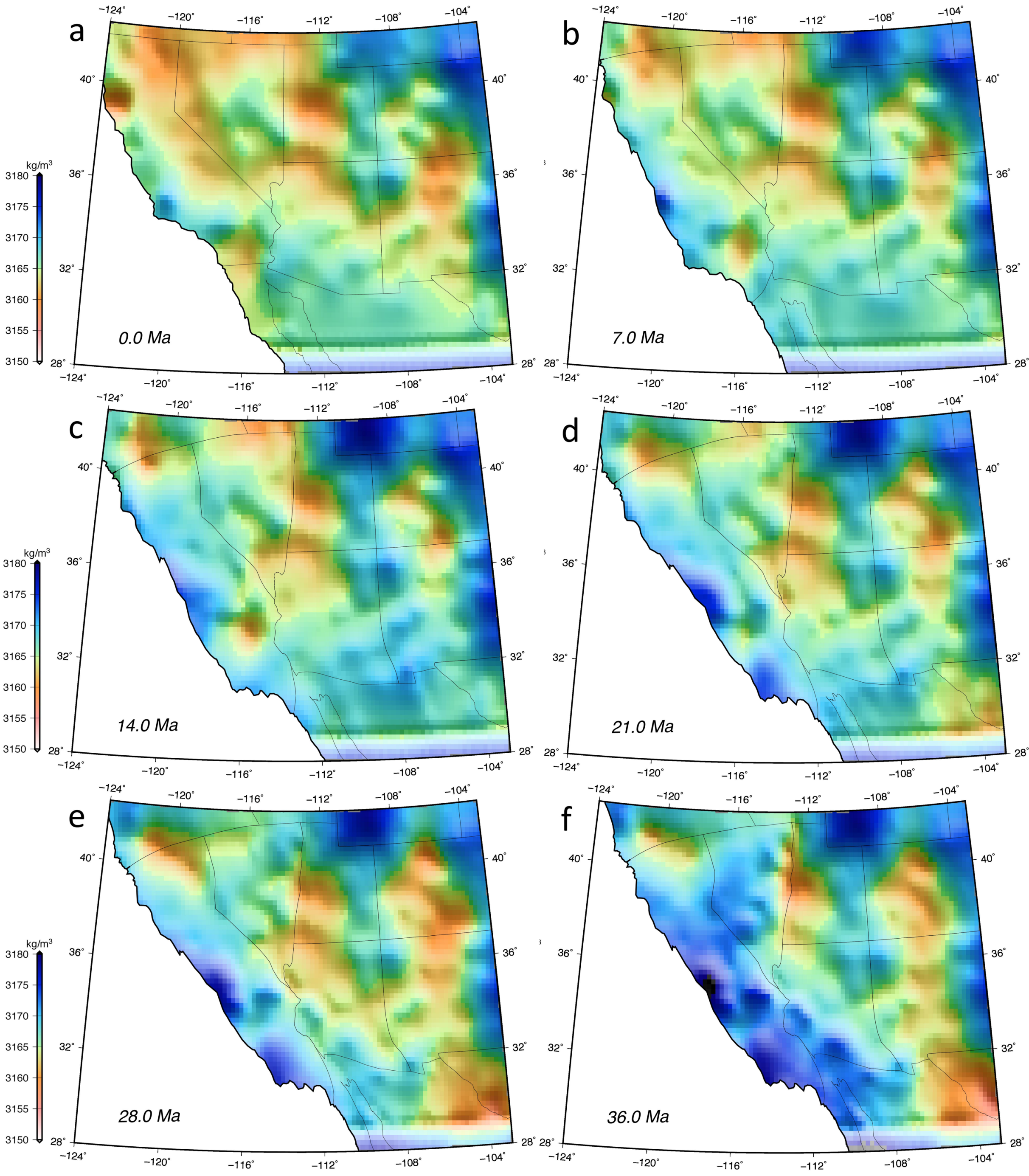

Supplementary Fig. 19 | a-f. Time-slice maps of upper mantle density changes associated with slab rollback in southwestern North America.

Supplementary Tables

Supplementary Table 1 | The average misfit estimates between model outputs from iteratively updated dynamic stress, strain rates and viscosities (blind test) and final complete solution (true solution).

Iteration	Number of areas	Total regional area	Average viscosity misfit (%)	Average strain misfit (%)	Average misfit to the functional	Average correlation between unit tensors	Average misfit between unit tensors	Vrms misfit (mm/yr)
1	703	4.42E-02	14.271	17.154	4.903E+10	0.831	0.227	1.453
2	703	4.42E-02	12.623	14.206	4.753E+10	0.856	0.194	1.281
3	703	4.42E-02	8.347	11.773	4.718E+10	0.871	0.185	0.904
4	703	4.42E-02	7.107	8.522	4.522E+10	0.883	0.180	0.822
5	703	4.42E-02	6.423	7.961	4.481E+10	0.912	0.162	0.781
6	703	4.42E-02	5.920	6.106	4.457E+10	0.937	0.147	0.615
7	703	4.42E-02	5.115	4.991	4.391E+10	0.951	0.130	0.505
8	703	4.42E-02	3.595	3.760	4.305E+10	0.966	0.126	0.383
9	703	4.42E-02	2.928	2.877	4.187E+10	0.979	0.113	0.340
10	703	4.42E-02	2.732	2.001	4.143E+10	0.986	0.108	0.325
11	703	4.42E-02	2.216	1.495	4.124E+10	0.992	0.096	0.315
12	703	4.42E-02	2.203	1.481	4.122E+10	0.997	0.096	0.313

Supplementary Table 2 | Average misfit between stretch directions of metamorphic core complexes, Miocene fault and dyke trends and the orientation of tensional deviatoric stresses achieved in iterative dynamic model through time in southwestern North America.

Time interval (Ma)	Name	Longitude at present-day	Latitude at present-day	Inferred Extension Direction for MCCS, Fault, Dyke	Stress Extensional Direction from iterative dynamic model	Average misfit
31 – 36 Ma	Albion[1]	-113.650002	42.5	289.9824423	283.1983337	6.78410867
	Grouse[1]	-114	41.75	302.1527913	280.819997	21.33279433
	Kern[1]	-114.199997	39.75	132.6844938	117.21833295	15.46616088
	Ruby[1]	-115.5	40.6500015	283.5383553	280.8000083	2.738347
	Raft[1]	-113.5	42	113.5261195	107.031662	6.4944575
	Snake[1]	-114.599998	39.2999992	120.1059047	114.1299947	5.97590997
19 – 30 Ma	Magdellena[1]	-111	30.6000004	214.6053951	252.6758333	38.07043825
	Mazatan[1]	-111	29.2000008	233.8182855	251.5208347	17.70254917
	Mezquital[1]	-111.300003	32	206.0065957	240.0291659	34.02257025
	Aconchi[1]	-110.300003	29.75	49.83706698	70.81083361	20.97376663
	Coyote[1]	-111.5	32	3.060871603	60.08916728	57.02829568
	Pozo[1]	-111.599998	31.5	196.155852	242.4866688	46.33081683
	Catalina[1]	-110.779999	32.4199982	230.9383203	238.0058316	7.067511333
	Comobabi[1]	-111.900002	32	2.985161821	60.49916586	57.51400404
	Tortolita[1]	-111	32.5	230.8099442	234.0775008	3.267556667
	Picacho[1]	-111.400002	32.7000008	224.4221915	231.8100001	7.387808583
	Pinaleno[1]	-109.900002	32.7000008	30.69258452	58.02750015	27.33491563
	White[1]	-112.260002	33.5699997	56.16617839	44.58499972	11.58117868
	South[1]	-111.82	33.2999992	53.02835432	46.36333371	6.665020608
	Central[1]	-117.160004	35.1800003	46.91509089	56.36666648	9.451575583
	Mojave[1]	-114.400002	34.6599998	49.86233362	42.58333333	7.279000283
	Rincon[1]	-110.529999	32.1800003	235.4020894	240.8675016	5.465412167
19 – 24 Ma	Monitor Range[2]	-116.330002	38.4799995	224.453926	249.0066657	24.55273967
	Kawich Range[2]	-116.279999	37.5800018	220.7616908	265.6483307	44.88663983
	Cactus Range[2]	-116.449997	37.3699989	226.8323745	265.6483307	38.81595617
	Cactus Range[2]	-116.440002	37.2999992	264.7923382	265.6483307	0.8559925
	Mellan Hills[2]	-116.580002	37.7000008	244.4486058	264.1433258	19.69472
	Kawich Range[2]	-116.279999	36.5800018	246.1613438	248.8916652	2.730321333
	Halfpint Range[2]	-115.550003	37.0699997	252.6225357	263.5033315	10.88079583
	Sheep Range[2]	-115.07	36.4799995	252.6189195	249.5983353	3.020584167
	Alamo[2]	-115.089996	37.2099991	259.034551	263.5033315	4.4687805
	Coyote Spring[2]	-114.900002	36.7999992	259.0467173	255.2616653	3.785052
	Eldorado Mountains[2]	-114.459999	35.4799995	252.7399875	222.7049993	30.03498817
	Calients[2]	-114.300003	37.3600006	233.9888227	256.3233388	22.33451617
	Bull Valley[2]	-113.540001	37.2799988	202.6365355	247.6766662	45.04013067
	Creek Range[2]	-114.199997	38.1100006	240.1957908	262.2833327	22.08754183
	Needle Mountains[2]	-107.370003	37.3699989	243.6461437	260.0299962	16.3838525
	Fortification Range[2]	-114.449997	38.5600014	252.6453477	268.449997	15.80464933
	Tunnel Spring Mounta[2]	-113.75	38.7700005	265.2178802	265.8283385	0.610458333
	Cowboy Pass[2]	-113.779999	38.8100014	233.7790985	265.8283385	32.04924
19 – 24 Ma	Oatman[3]	-114.370003	35.0699997	37.27213542	42.8849996	5.612864183
	Castaned Hills[3]	-113.919998	34.4199982	37.2640813	43.00666683	5.742585533
	Vulture Mountains[3]	-112.900002	33.7999992	78.23167038	50.89666683	27.33500355
	Castle Dome[3]	-114.099998	33.0999985	37.02306428	59.3599987	22.33693442
	Ajo[3]	-112.75	32.4700012	37.15955735	58.88666598	21.72710863
	Mineral Mountain[3]	-111.199997	33.0999985	55.98940787	50.37333425	5.616073617
	Klondyke[3]	-110.349998	32.9000015	65.57797495	58.54500008	7.032974867
	Silver Bell[3]	-111.599998	32.4000015	65.61956533	55.54999985	10.06956548
	Papago Indian[3]	-111.379997	32.2000008	59.4638888	59.14166767	0.322221133
	Oro Blanco[3]	-111.400002	31.5	47.04710703	60.65166602	13.60455898
	Knight Peak[3]	-108.330002	32.3699989	40.16378148	62.96166612	22.79788463
	Cedar Mountains[3]	-108.050003	31.9500008	27.94812457	63.43500135	35.48687678
	Truth[3]	-107.150002	33.0800018	43.25058365	75.5583331	32.30774945
	Magdalena[3]	-107.370003	34.3699989	80.74910737	87.12000022	6.37089285
	Socorro[3]	-108.080002	34.3300018	60.41447448	82.70333225	22.28885777
	Spanish Peaks[3]	-104.879997	37.6199989	5.988187153	94.45333222	88.46514506
	Telluride[3]	-107.889999	37.7099991	57.10023245	79.8366661	22.73643365
	Glenwood Springs[3]	-107.25	39.9000015	37.59435398	66.87000022	29.27564623
	Steamboat Springs[3]	-106.949997	40.2000008	38.70222727	68.85333443	30.15110717
	Routt[3]	-107.099998	40.7999992	40.20300802	62.54833412	22.3453261
13 – 18 Ma	Sacramentos[1]	-115	35	56.04884762	54.10388884	1.944958778
	Harcuvar[1]	-113.029999	33.9000015	53.04838921	53.40888871	0.360499506
	Harquahalla[1]	-112.699997	33.7999992	53.00890054	51.6572219	1.351678644
	Buckskin[1]	-113.370003	34.1599998	37.19368552	49.60333294	12.40964742
	Whipple[1]	-114.169998	34.2999992	43.4364711	54.59666676	11.16019566
	Chemehuevi[1]	-114.480003	34.5499992	49.91276613	51.89388932	1.981123189
13– 18 Ma	Monitor Range[2]	-116.330002	38.4799995	224.5335897	249.978335	25.44474533
	Kawich Range[2]	-116.279999	37.5800018	219.9328613	258.5799967	38.64713533
	Cactus Range[2]	-116.449997	37.3699989	225.8638942	257.8749948	32.01110067
	Cactus Range[2]	-116.440002	37.2999992	263.7628887	257.8749948	5.887893833
	Mellan Hills[2]	-116.580002	37.7000008	243.8051325	253.1833318	9.378199333
	Kawich Range[2]	-116.279999	36.5800018	245.8331705	261.3083267	15.47515617
	Halfpint Range[2]	-115.550003	37.0699997	252.159246	261.7266618	9.567415833
	Sheep Range[2]	-115.07	36.4799995	252.36526	259.7033312	7.338071167
	Alamo[2]	-115.089996	37.2099991	258.6275788	262.781662	4.154083167
	Coyote Spring[2]	-114.900002	36.7999992	258.7339733	261.5966698	2.8626965
	Eldorado Mountains[2]	-114.459999	35.4799995	252.4040452	248.1716615	4.232383667
	Calients[2]	-114.300003	37.3600006	233.7880327	259.1816763	25.39364367
	Bull Valley[2]	-113.540001	37.2799988	202.526558	253.0866648	50.56010683

	Creek Range[2]	-114.199997	38.1100006	240.0419922	265.693339	25.65134683
	Needle Mountains[2]	-107.370003	37.3699989	243.2317072	249.2333323	6.001625167
	Fortification Range[2]	-114.449997	38.5600014	252.5091248	265.9266562	13.41753133
	Tunnel Spring Mounta[2]	-113.75	38.7700005	265.111847	268.6899922	3.578145167
	Cowboy Pass[2]	-113.779999	38.8100014	233.6866887	268.6899922	35.0033035
13 – 18 Ma	Oatman[3]	-114.370003	35.0699997	37.18368658	67.02999942	29.84631283
	Castaned Hills[3]	-113.919998	34.4199982	36.6227951	64.92000007	28.29720497
	Vulture Mountains[3]	-112.900002	33.7999992	78.09731165	64.14666622	13.95064543
	Castle Dome[3]	-114.099998	33.0999985	37.42924307	70.05166752	32.62242445
	Ajo[3]	-112.75	32.4700012	36.40239778	65.80833308	29.4059353
	Mineral Mountain[3]	-111.199997	33.0999985	55.85166487	65.94500033	10.09333547
	Klondyke[3]	-110.349998	32.9000015	66.8672956	65.7033348	1.1639608
	Silver Bell[3]	-111.599998	32.4000015	65.7495753	63.87166597	1.877909333
	Papago Indian[3]	-111.379997	32.2000008	60.68721833	62.32999867	1.642780333
	Oro Blanco[3]	-111.400002	31.5	47.62966157	64.6516679	17.02200633
	Knight Peak[3]	-108.330002	32.3699989	39.62526322	68.8916664	29.26640318
	Cedar Mountains[3]	-108.050003	31.9500008	27.71710205	69.58833315	41.8712311
	Truth[3]	-107.150002	33.0800018	42.92704963	75.5766678	32.64961817
	Magdalena[3]	-107.370003	34.3699989	80.4533043	84.16666667	3.713362367
	Socorro[3]	-108.080002	34.3300018	59.70257123	78.24333317	18.54076193
	Spanish Peaks[3]	-104.879997	37.6199989	5.954289118	80.41000053	74.45571142
	Telluride[3]	-107.889999	37.7099991	57.80673027	66.32833355	8.521603283
	Glenwood Springs[3]	-107.25	39.9000015	38.37958653	58.25333278	19.87374625
	Steamboat Springs[3]	-106.949997	40.2000008	39.06927808	57.96833355	18.89905547
	Routt[3]	-107.099998	40.7999992	39.95105108	51.28999965	11.33894857
5 – 12 Ma	Badwater[1]	-116.75	36.2999992	295.8283805	274.9275018	20.90087875
	Bullfrog[1]	-116.75	37	283.151703	271.4200021	11.73170088
	Funeral[1]	-116.599998	36.4000015	314.723507	272.1575014	42.56600563
	Mineral[1]	-117.599998	37.75	301.0836488	273.0774956	28.00615313
5 – 12 Ma	Monitor Range[2]	-116.330002	38.4799995	226.4475073	269.2833252	42.83581783
	Kawich Range[2]	-116.279999	37.5800018	220.4702683	272.2983297	51.82806133
	Cactus Range[2]	-116.449997	37.3699989	226.7169877	271.7016602	44.9846725
	Cactus Range[2]	-116.440002	37.2999992	264.4241943	271.7016602	7.277465833
	Mellan Hills[2]	-116.580002	37.7000008	245.3884708	270.3483328	24.959862
	Kawich Range[2]	-116.279999	36.5800018	245.7819137	270.3366648	24.55475117
	Halfpint Range[2]	-115.550003	37.0699997	252.224121	273.7733355	21.5492145
	Sheep Range[2]	-115.07	36.4799995	252.3755137	264.9350027	12.559489
	Alamo[2]	-115.089996	37.2099991	258.4108532	274.2816722	15.870819
	Coyote Spring[2]	-114.900002	36.7999992	257.3133317	269.8849945	12.57166283
	Eldorado Mountains[2]	-114.459999	35.4799995	249.9554545	264.0750072	14.11955267
	Calients[2]	-114.300003	37.3600006	233.9654592	273.1383362	39.172877
	Bull Valley[2]	-113.540001	37.2799988	202.9083378	273.9933318	71.084994
	Creek Range[2]	-114.199997	38.1100006	239.8471017	271.8500008	32.00289917
	Needle Mountains[2]	-107.370003	37.3699989	243.7825062	257.1650035	13.38249733
	Fortification Range[2]	-114.449997	38.5600014	252.505656	267.999995	15.494339
	Tunnel Spring Mounta[2]	-113.75	38.7700005	264.933899	270.2650045	5.3311055
	Cowboy Pass[2]	-113.779999	38.8100014	233.5501965	270.0516715	36.501475
5 – 12 Ma	Oatman[3]	-114.370003	35.0699997	36.2862428	84.61666488	48.33042208
	Castaned Hills[3]	-113.919998	34.4199982	35.25890283	81.97499975	46.71609692
	Vulture Mountains[3]	-112.900002	33.7999992	80.09201812	71.28666815	8.805349967
	Castle Dome[3]	-114.099998	33.0999985	43.98930232	71.62166722	27.6323649
	Ajo[3]	-112.75	32.4700012	41.85564423	67.8466676	25.99102337
	Mineral Mountain[3]	-111.199997	33.0999985	59.5740242	68.19166627	8.617642067
	Klondyke[3]	-110.349998	32.9000015	71.07621637	70.42833328	0.647883083
	Silver Bell[3]	-111.599998	32.4000015	70.3623352	66.84166717	3.520668033
	Papago Indian[3]	-111.379997	32.2000008	66.0099144	67.64833323	1.638418833
	Oro Blanco[3]	-111.400002	31.5	51.9355615	77.21166738	25.27610588
	Knight Peak[3]	-108.330002	32.3699989	40.68180148	72.13166745	31.44986597
	Cedar Mountains[3]	-108.050003	31.9500008	29.09909567	70.39833323	41.29923757
	Truth[3]	-107.150002	33.0800018	41.95743878	90.56666437	48.60922558
	Magdalena[3]	-107.370003	34.3699989	79.46215057	92.69832865	13.23617808
	Socorro[3]	-108.080002	34.3300018	59.83588283	89.76666512	29.93078228
	Spanish Peaks[3]	-104.879997	37.6199989	6.492570242	73.32000098	66.82743074
	Telluride[3]	-107.889999	37.7099991	58.72798477	79.4800021	20.75201733
	Glenwood Springs[3]	-107.25	39.9000015	39.20228577	65.3699995	26.16771374
	Steamboat Springs[3]	-106.949997	40.2000008	39.504776	68.00666738	28.50189138
	Routt[3]	-107.099998	40.7999992	40.08700752	56.17499927	16.08799175

[1] : Metamorphic Core Complex

[2] : Miocene Fault

[3] : Miocene Dyke

Supplementary References

1. Bahadori, A., Holt, W. E. & Rasbury, E. T. Reconstruction modeling of crustal thickness and paleotopography of western North America since 36 Ma. *Geosphere* **14**, 1207–1231 (2018).
2. Flesch, L. M. Dynamics of the Pacific-North American Plate Boundary in the Western United States. *Science* **287**, 834–836 (2000).
3. Flesch, L. M., Holt, W. E., Haines, A. J., Wen, L. & Shen-Tu, B. The dynamics of western North America: stress magnitudes and the relative role of gravitational potential energy, plate interaction at the boundary and basal tractions. *Geophys. J. Int.* **169**, 866–896 (2007).
4. Flesch, L. M., Haines, A. J. & Holt, W. E. Dynamics of the India-Eurasia collision zone. *J. Geophys. Res. Solid Earth* **106**, 16435–16460 (2001).
5. Bird, R. B. New Variational Principle for Incompressible Non-Newtonian Flow. *Phys. Fluids* **3**, 539 (1960).
6. England, P. & McKenzie, D. A thin viscous sheet model for continental deformation. *Geophys. J. Int.* **70**, 295–321 (1982).
7. Sonder, L. J. & England, P. Vertical averages of rheology of the continental lithosphere: relation to thin sheet parameters. *Earth Planet. Sci. Lett.* **77**, 81–90 (1986).
8. Behr, W. M. & Platt, J. P. A naturally constrained stress profile through the middle crust in an extensional terrane. *Earth Planet. Sci. Lett.* **303**, 181–192 (2011).
9. Atwater, T. & Stock, J. Pacific-North America Plate Tectonics of the Neogene Southwestern United States: An Update. *Int. Geol. Rev.* **40**, 375–402 (1998).
10. DeMets, C. & Dixon, T. H. New kinematic models for Pacific-North America motion from 3 Ma to present, I: Evidence for steady motion and biases in the NUVEL-1A Model. *Geophys. Res. Lett.* **26**, 1921–1924 (1999).
11. Haines, A. J. & Holt, W. E. A procedure for obtaining the complete horizontal motions within zones of distributed deformation from the inversion of strain rate data. *J. Geophys. Res. Solid Earth* **98**, 12057–12082 (1993).
12. McKenzie, D. & Jackson, J. Conditions for flow in the continental crust: CONDITIONS FOR FLOW IN THE CONTINENTAL CRUST. *Tectonics* **21**, 5-1-5–7 (2002).
13. Bomberger, C., Bendick, R., Flesch, L. & Ehlers, T. A. Spatial Scales in Topography and Strain Rate Magnitude in the Western United States. *J. Geophys. Res. Solid Earth* **123**, 6086–6097 (2018).
14. Shen, W. & Ritzwoller, M. H. Crustal and uppermost mantle structure beneath the United States: CRUSTAL AND UPPERMOST MANTLE MODEL OF U.S. *J. Geophys. Res. Solid Earth* **121**, 4306–4342 (2016).
15. The Western North American Volcanic and Intrusive Rock Database. Available at: http://www.navdat.org/. (Accessed: 20th December 2017)
16. Ghosh, A., Holt, W. E. & Wen, L. Predicting the lithospheric stress field and plate motions by joint modeling of lithosphere and mantle dynamics. *J. Geophys. Res. Solid Earth* **118**, 346–368 (2013).
17. Wang, X., Holt, W. E. & Ghosh, A. Joint modeling of lithosphere and mantle dynamics: Evaluation of constraints from global tomography models: EVALUATION OF GLOBAL TOMOGRAPHY MODELS. *J. Geophys. Res. Solid Earth* **120**, 8633–8655 (2015).
18. Gerya, T. V., Stern, R. J., Baes, M., Sobolev, S. V. & Whattam, S. A. Plate tectonics on the Earth triggered by plume-induced subduction initiation. *Nature* **527**, 221–225 (2015).
19. Gerya, T. V. & Meilick, F. I. Geodynamic regimes of subduction under an active margin: effects of rheological weakening by fluids and melts: GEODYNAMIC REGIMES OF SUBDUCTION. *J. Metamorph. Geol.* **29**, 7–31 (2011).
20. Dixon, J. E., Dixon, T. H., Bell, D. R. & Malservisi, R. Lateral variation in upper mantle viscosity: role of water. *Earth Planet. Sci. Lett.* **222**, 451–467 (2004).
