# Supplementary material for: Geodynamic evolution of southwestern North America since the Late Eocene
Source: Nat Commun. 2019 Nov 18;10:5213. doi: 10.1038/s41467-019-12950-8 (PMC6861285; doi:10.1038/s41467-019-12950-8)
Supplement: Supplementary file 1 — Peer Review File [file 41467_2019_12950_MOESM1_ESM.pdf]

Editorial Note: This manuscript has been previously reviewed at another journal that is not operating a transparent peer review scheme. The manuscript was considered suitable for publication without further review at Nature Communications.
